# Functional divergence and origin of the *DAG-like* gene family in plants

**DOI:** 10.1038/s41598-017-05961-2

**Published:** 2017-07-18

**Authors:** Meijie Luo, Manjun Cai, Jianhua Zhang, Yurong Li, Ruyang Zhang, Wei Song, Ke Zhang, Hailin Xiao, Bing Yue, Yonglian Zheng, Yanxin Zhao, Jiuran Zhao, Fazhan Qiu

**Affiliations:** 1 Beijing Key Laboratory of Maize DNA Fingerprinting and Molecular Breeding, Maize Research Center, Beijing Academy of Agriculture and Forestry Sciences, Beijing, 100097 China; 2 National Key Laboratory of Crop Genetic Improvement, Huazhong Agricultural University, Wuhan, 430070 China; 3 Life Science and Technology Center, China National Seed Group Co., Ltd., Wuhan, 430075 China

## Abstract

The nuclear-encoded *DAG-like* (*DAL*) gene family plays critical roles in organelle C-to-U RNA editing in *Arabidopsis thaliana*. However, the origin, diversification and functional divergence of *DAL* genes remain unclear. Here, we analyzed the genomes of diverse plant species and found that: *DAL* genes are specific to spermatophytes, all *DAL* genes share a conserved gene structure and protein similarity with the inhibitor I9 domain of subtilisin genes found in ferns and mosses, suggesting that *DAL* genes likely arose from I9-containing proproteases via exon shuffling. Based on phylogenetic inference, *DAL* genes can be divided into five subfamilies, each composed of putatively orthologous and paralogous genes from different species, suggesting that all *DAL* genes originated from a common ancestor in early seed plants. Significant type I functional divergence was observed in 6 of 10 pairwise comparisons, indicating that shifting functional constraints have contributed to the evolution of *DAL* genes. This inference is supported by the finding that functionally divergent amino acids between subfamilies are predominantly located in the DAL domain, a critical part of the RNA editosome. Overall, these findings shed light on the origin of *DAL* genes in spermatophytes and outline functionally important residues involved in the complexity of the RNA editosome.

## Introduction

C-to-U RNA editing (deamination of cytidine to uridine) is an essential step of RNA maturation in chloroplasts and mitochondria of land plants from bryophytes to angiosperms^1, 2^. U-to-C RNA editing is also observed in ferns and mosses^3, 4^. More than 400 editing sites in mitochondria and 30–40 editing events in chloroplasts are typically found in flowering plants^5–7^. RNA editing occurring in plant organelle mRNAs can restore functionally conserved amino acids at the post-transcriptional level, create functional proteins and play important roles in efficient splicing of introns and processing of precursor tRNA molecules^7–9^. In plants, some mutants with impaired RNA editing at specific nucleotide sites cause deleterious phenotypes and even lethality. The site specificity of the cytidines to be edited in a plant organelle is determined by a crucial *cis*-acting regulatory sequence^10–14^ and the RNA editosome that will bind to it. The RNA editosome is composed of nuclear-encoded *trans*-acting factors that recognize the *cis*-element and perform RNA editing^15^. Recent extensive genetics studies have revealed that these *trans*-factors enlisted in the RNA editosome include DYW-type pentatricopeptide repeat (PPR) proteins^1^, RNA-Editing Factor Interacting Protein (RIP) family or Multiple Organelle RNA Editing Factor (MORF) family proteins^16, 17^, RNA-recognition motif (RRM)-containing proteins^18–20^, protoporphyrinogen IX oxidase 1 (PPO1)^21^ and organelle zinc-finger 1 (OZ1)^22^. PPR proteins are characterized by tandem 35-amino acid PPR motifs^23^. The DYW-PPR proteins each recognize one or a few editing sites that have similar *cis*-elements and thereby bind directly to the *cis*-acting sequences. The DYW domain of DYW-PPR proteins has a sequence similar to the active sites of known cytidine deaminases and editing enzymes^24^, and may be responsible for deamination of cytidine to uridine.

The *RIP*/*MORF* gene family, which controls multiple organelle RNA editing sites, was identified in *Arabidopsis thaliana* and designated the *RIP* gene family and the *MORF* gene family by two research groups^16, 17^. Here, we adopt the name, the *DAG-like* (*DAL*) gene family, based on the first identified member (*DAG*) of the gene family in *Antirrhinum majus* ^25, 26^. *Arabidopsis* DAL proteins are all targeted to mitochondria or chloroplasts and required for RNA editing at all sites in both organelles^16, 17^. The mutation of *DAL* genes in plants results in abnormal development of plants, even lethality^16, 17, 25–27^. Yeast two-hybrid analysis confirmed that DAL/RIP/MORF proteins can interact selectively with diverse PPR proteins by the binding of the DAL domain to PPR motifs^17^, and moreover, DAL proteins can connect to form hetero- and homodimers^16^. A variation of the *DAL* gene (*ORRM1*) was identified and functionally analyzed; it harbors a pair of truncated RIP domains (RIP-RIP) at its N terminus and an RRM domain at its C terminus^28^. ORRM1 is an essential plastid editing factor that can interact selectively with PPR proteins via its RIP-RIP domain, and the ORRM1 protein can also bind to sequences near at least some of its RNA targets *in vitro* ^28^. Furthermore, the RRM domain can rescue the editing defect in *orrm1* protoplasts independent of RIP domains, and three other RRM-containing proteins were identified because of their roles in organelle RNA editing, suggesting that the RRM domain participates in the RNA editosome^18–20^. Together, DAL proteins may be connectors between the site-specific PPRs and the as-yet-unknown deaminase or other components in the RNA editosome, such as RRM-containing proteins, PPO1^21^ and OZ1^22^.

Compared with the RNA editosomes responsible for C-to-U or A-to-I (deamination of adenosine to inosine) RNA editing in mammals, the plant organelle RNA editosomes have more diverse components^15^. In addition to the interpretation that more RNA-edited sites in plant organelles require more *trans*-acting editing factors, the diverse composition of the organelle RNA editosome in plants probably overcomes the deficiency in RNA editing caused by the mutation of PPR protein or changes in the *cis*-acting sequences of edited sites^15^, especially for those edited sites in plant mitochondrial genomes which evolve much more quickly. Thus, the origin, classification and evolution analysis of *trans*-acting factors is important for understanding the evolution and molecular mechanism of the RNA editosome in plants. In the RNA editosome, *DYW-PPR* genes undergo purifying selection at sites targeted for RNA editing because they are important for recognizing *cis*-element sequences^1, 29, 30^. However, the functional evolution and origin of the *DAL* gene family is unknown.

In this study, we identified the DAL proteins in various plant lineages, including green algae, moss, ferns, gymnosperms and flowering plants, to investigate functional divergence and origin of the *DAL* gene family in plants. The result indicated *DAL* genes are specific to spermatophytes other than to lower plants. Plant *DAL* genes shared a strong conserved gene structure and appear to have evolved from the I9-containing proprotease via exon shuffling. Functional divergence analysis revealed that there was significant functional divergence between different DAL clades which may be associated with differences in the roles different *DAL* genes play in RNA editing and RNA metabolism. The evolutionary and functional divergence analysis of the *DAL* genes in plants presented here provides useful information for further probing the molecular mechanism by which DAL proteins contribute to the RNA editosome.

## Results

### Identification and sequence analysis of *DAL* genes in maize

**Figure 1:**
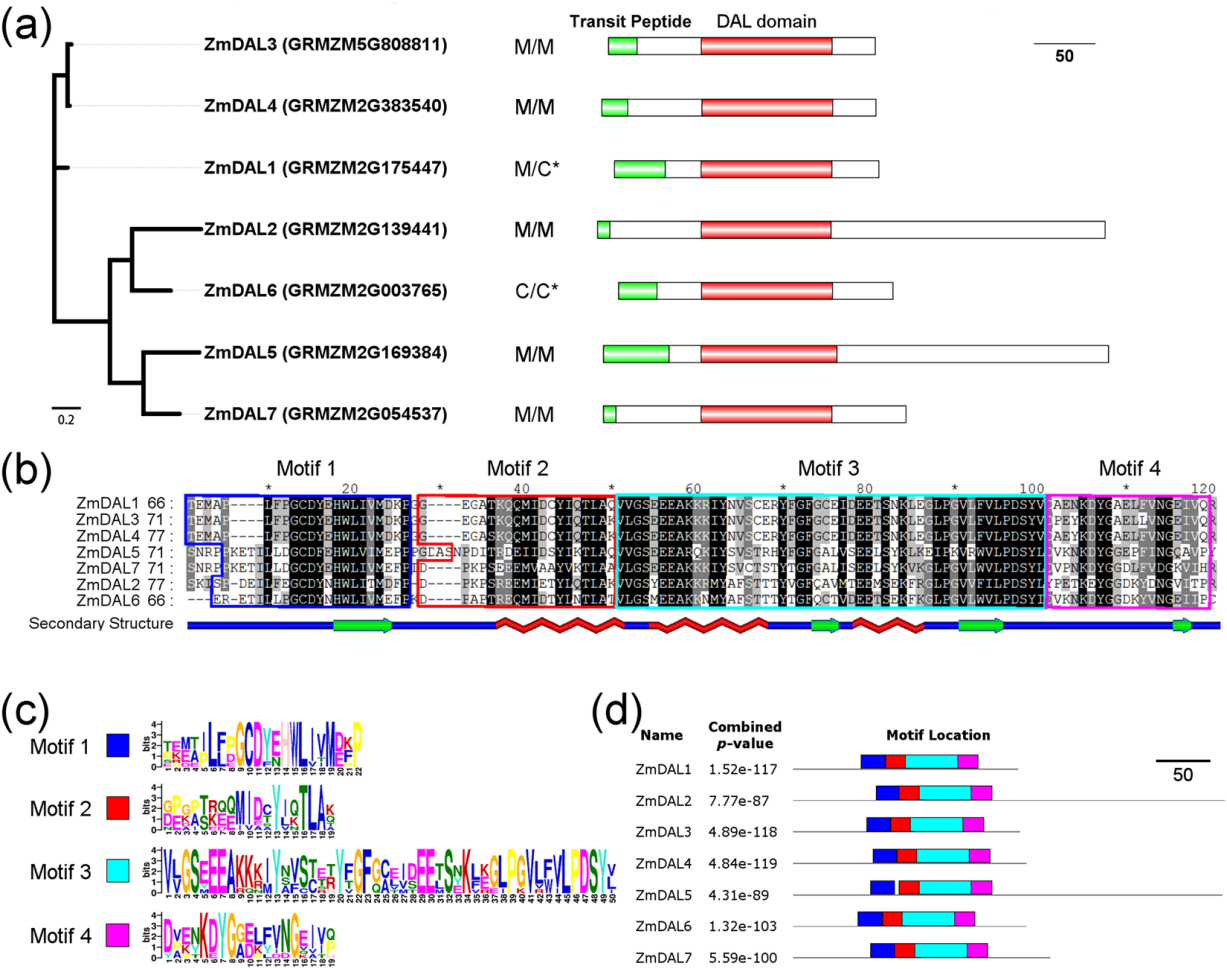
Maize DAL proteins and their conserved motifs. (**a**) Multiple sequence alignment of maize DAL proteins was carried out using ClustalW2.0, and the NJ tree was built using MEGA v5.0. The chloroplast and mitochondrial transit peptides of maize DAL proteins were predicted using Predotar (http://urgi.versailles.inra.fr/predotar/predotar.html) and (/) TargetP (http://www.cbs.dtu.dk/services/TargetP/). M, mitochondria; C, chloroplast. The DAL proteins marked by an asterisk were observed in the plastid proteome. (**b**) Alignment of conserved DAL domains in maize DAL proteins was conducted using ClustalW2.0 and displayed with GeneDoc. The secondary structure of the DAL domain was inferred using MINNOU (http://minnou.cchmc.org/). (**c**,**d**) Putative motifs were explored using the MEME server with the parameters of between 10 and 50 aa in length and sharing of each motif among all ZmDAL proteins.

To identify putative *DAL* genes in the maize genome, we searched the maize genome annotation data with known plant DAL proteins as a query. In total, we obtained 7 putative *DAL* genes in maize named *ZmDAL1*—*ZmDAL7* based on their order on the chromosomes (Fig. 1a and Supplementary Table S1). *ZmDAL*s were distributed on 5 of 10 maize chromosomes, and chromosomes 9 and 10 both had two *ZmDAL* genes (Supplementary Fig. S1). The gene model of *ZmDAL1* was reannotated correctly by analyzing the similarity between *ZmDAL* genes and their orthologs (Supplementary Fig. S2). The veracity of each gene model of *ZmDAL* genes was assessed using reverse transcription polymerase chain reaction (RT-PCR) assays with the gene-specific primers listed in Supplementary Table S2, as 4 of 7 *ZmDAL* genes had more than one transcript for each *ZmDAL* gene in the MaizeGDB database (http://www.maizegdb.org/). The RT-PCR results indicated that seven *ZmDAL* genes were expressed in maize seedlings and only a single transcript was found for each *ZmDAL* gene (Supplementary Fig. S3). All identified maize *DAL* genes encoded proteins ranging from 215 (ZmDAL1) to 412 amino acids (aa) (ZmDAL2), and their isoelectric points (Ip) were similar (>8.0).

No known motif was found in the maize DAL proteins by screening the PFAM and INTERPRO databases, except the MORF box (called the DAL domain in this study) which had been identified previously^16, 17^. Novel putative motifs were explored using the MEME server with different motif lengths. By selecting a motif length between 10 and 50 aa, we identified 4 conserved motifs, and all 4 motifs were located in the DAL domain (Fig. 1b,c,d), suggesting that the DAL domain is a conserved sequence among *Arabidopsis* and maize DAL proteins. To obtain an intact motif containing the DAL domain, we enlarged the MEME motif length and identified one motif containing 114 aa (Supplementary Fig. S4). Like their homologs in *Arabidopsis* ^16^, maize DAL proteins were predicted using TargetP and Predotar to enter mitochondria or chloroplasts. Of them ZmDAL1 and ZmDAL6 were also detected in the plastid nucleoid proteome by searching the maize organelle proteomics database (PPDB, http://ppdb.tc.cornell.edu/) (Fig. 1a).

**Figure 2:**
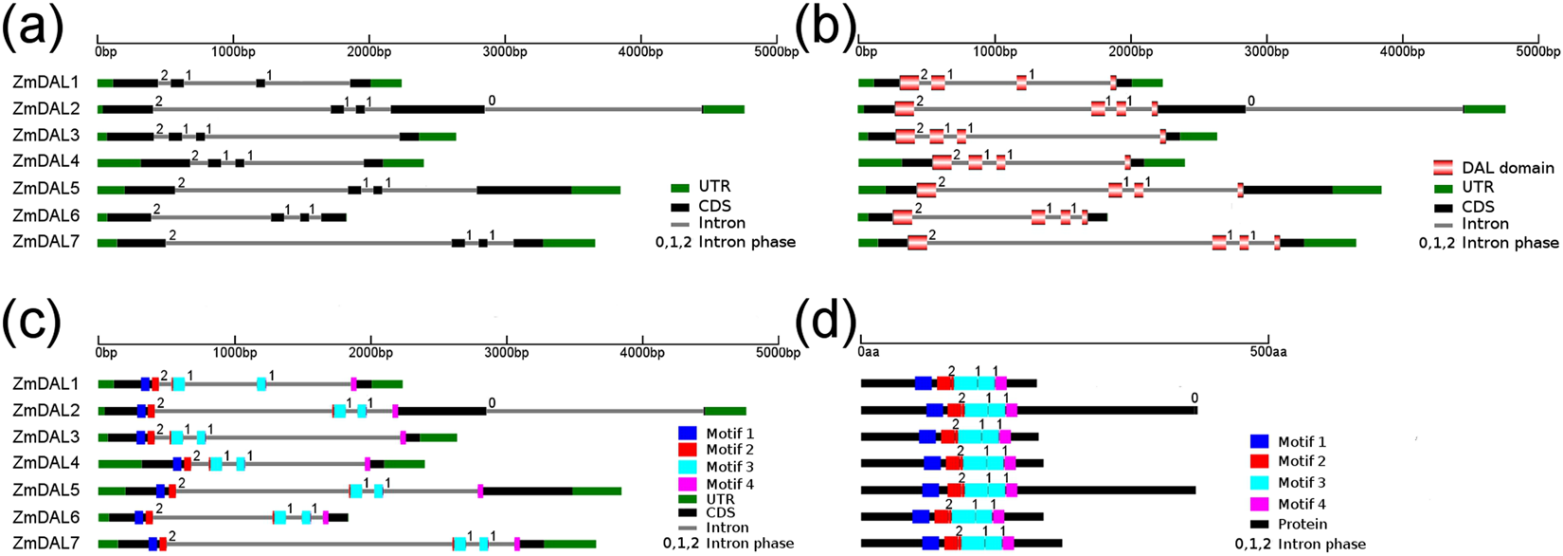
Conserved gene structures of maize *DAL* genes. The gene structures of *ZmDAL* genes were built using GSDraw (http://wheat.pw.usda.gov/piece/GSDraw.php) through both alignment of DNA obtained from MaizeGDB (http://www.maizegdb.org/) and coding sequences (CDS) of *ZmDAL* genes.

The gene structures of the *ZmDAL* genes were constructed by aligning the extracted genomic sequences to predicted cDNA sequences of maize *DAL* genes. This showed that *ZmDAL* genes have a conserved gene structure (Fig. 2); each of the *ZmDAL* genes has 3 introns with the intron phases 2, 1 and 1 separating DAL domain-encoding exons 1, 2, 3 and 4 (Fig. 2b). Motifs 1 and 2 are encoded by exon 1; motif 3 is encoded by exons 2 and 3; and motif 4 is located in exon 4 (Fig. 2c and d). Furthermore, the length of exons 2 (98 basepairs, bp) and 3 (66 bp) is conserved among all five *ZmDAL* genes, even though the size of the introns between the exons varies between different *ZmDAL* genes.

### Identification and phylogenetic analysis of plant *DAL* genes

**Figure 3:**
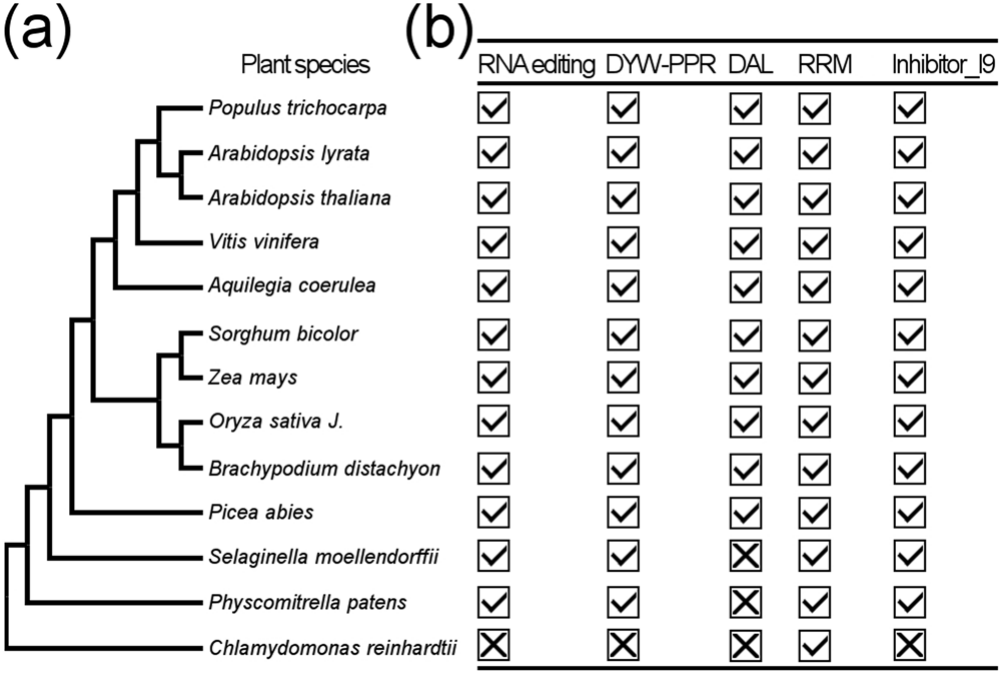
Distribution of *DAL* genes in the plant kingdom. (**a**) A schematic diagram of plant evolution tree was constructed according to the plant tree shown in PGDD (http://chibba.agtec.uga.edu/duplication/). (**b**) RNA editing sites (organelle C-to-U RNA editing), DYW-type *PPR* genes, *DAG-like* genes, *ORRM* genes, and *Inhibitor I9* genes were identified in the plants listed on the left. The checkmark in the box denotes that the above genes can be found in the corresponding genomes, and the cross in the box indicates none of above genes are found in these genomes.

To mine more DAL domain-encoding genes in plants, we used the HMMER 3.0 package^31^ to build a hidden Markov model (HMM) file (dal.hmm, Supplementary Data File S1) with 17 DAL domain sequences of those DAL proteins from *A*. *majus*, maize and *Arabidopsis* (Supplemental Data File S2). We then used the dal.hmm algorithm to query the genomes of a variety of plants representing the major evolutionary lineages, including *Chlamydomonas reinhardtii*, *Physcomitrella patens*, *Selaginella moellendorffii*, *Picea abies*, *Brachypodium distachyon*, *Oryza sativa* Japonica, *Zea mays*, *Sorghum bicolor*, *Aquilegia coerulea*, *Vitis vinifera*, *A*. *thaliana*, *Arabidopsis lyrata* and *Populus trichocarpa*. The result showed that putative *DAL* genes were only identified in seed plants but not in lower plants (*C*. *reinhardtii*, *P*. *patens* and *S*. *moellendorffii*) (Fig. 3). The numbers of *DAL* genes of higher plants used here are comparable, ranging from 6 (in *A*. *coerulea*) to 11 (in *A*. *lyrata*). In total, 79 *DAL* genes were identified in 10 plant genomes (Supplementary Table S3). In addition, we identified *ORRM1-like* genes in this study that were also specific to seed plants, and these genes encoded two tandem truncated DAL domains at the N terminus and one RNA recognition motif (RRM) at the C terminus, except the MA_10436715g0010 protein found in *P*. *abies*, which had no C-terminal RRM domain (Supplementary Fig. S5).

**Figure 4:**
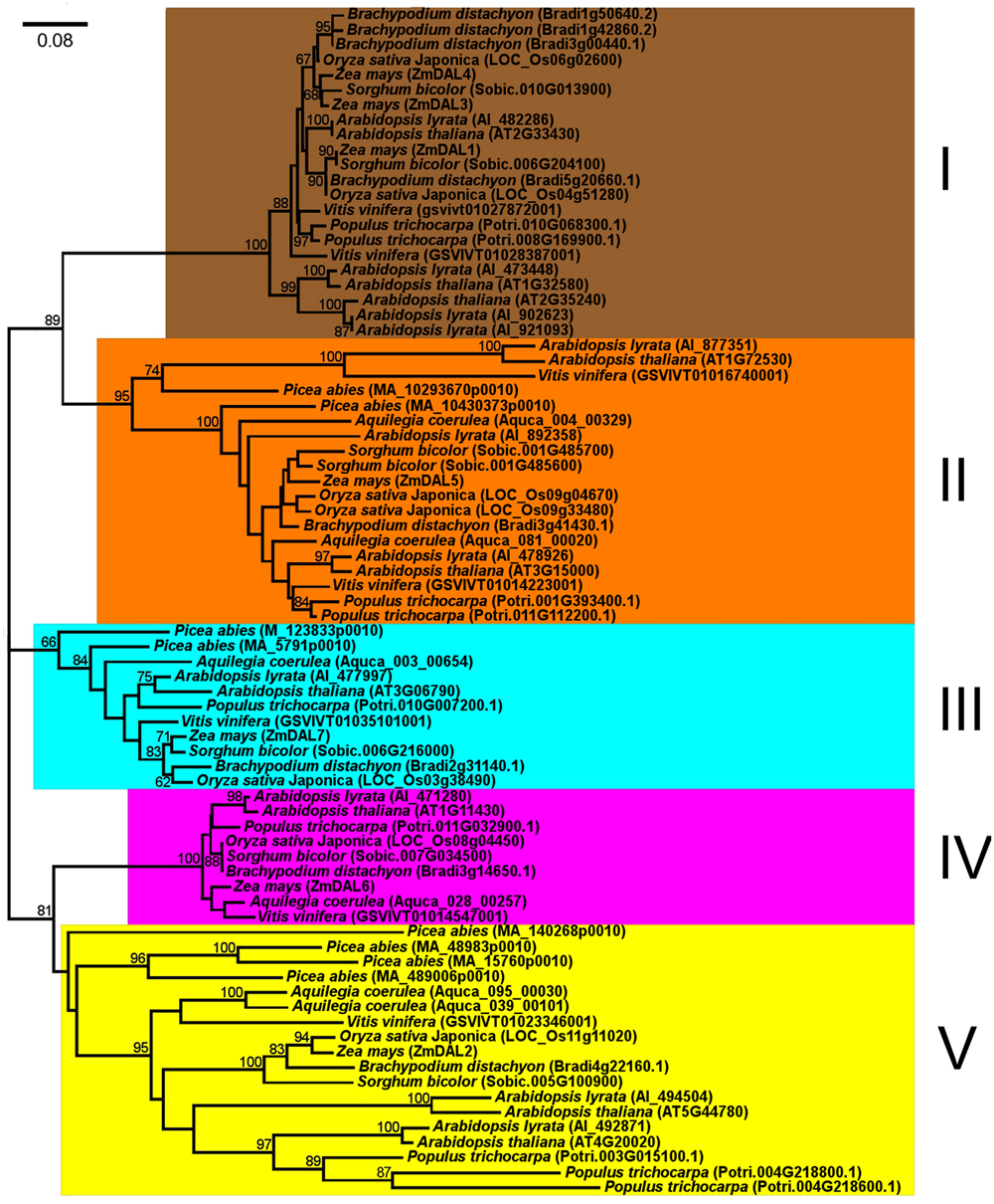
Phylogenetic relationship of plant DAL proteins. The neighbor-joining (NJ) phylogenetic tree of 79 plant *DAL* genes was constructed based on the multiple protein sequence alignment of the conserved regions (Supplemental Data File S3) according to the Poisson model. Bootstrap values >60% are indicated at each node, and the scale bar denotes the substitute rate per site. The species names are displayed before plant *DAL* genes.

To investigate the phylogenetic relationship among plant *DAL* genes, an unrooted neighbor-joining (NJ) tree containing all 79 DAL proteins was generated based on the conserved DAL domain alignment (Figs 4 and S5). On the basis of the phylogeny, the *DAL* gene family in plants was subdivided into five groups, named group I to group V (Fig. 4). In the NJ tree shown in Fig. 4, *DAL* genes of each group were all from diverse plant species. In groups I, II and V, species-specific gene duplication events occurred after the lineages diverged, resulting in the inclusion of more than one *DAL* gene per species (Fig. 4). Since the *DAL* genes were found to be specific to spermatophytes, we can infer that the ancestral *DAL* gene appeared after the divergence of seed plants and ferns.

The exon-intron organization analysis of 79 plant *DAL* genes indicated that plant *DAL* genes all share a conserved gene structure, with the 2-1-1 intron phase pattern separating DAL domain-encoding exons, as observed in maize *DAL* genes, except *Al_477997* and *MA_489006g0010*, which have intron phase patterns 0-1-1 and 0-2-2, respectively (Supplementary Fig. S6).

### The source of plant *DAL* genes

**Figure 5:**
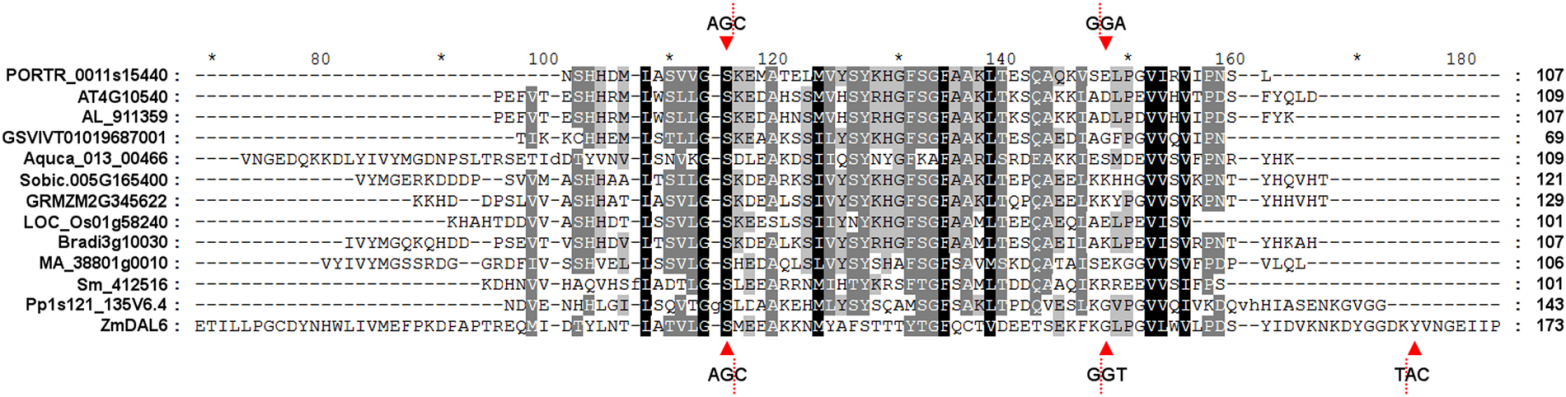
Alignment of inhibitor I9 domains and the DAL domain. A multiple protein sequence alignment was performed using hmmalign in the HMMER 3.0 package^28^ and was displayed using GeneDoc (http://www.nrbsc.org/gfx/genedoc/ebinet.htm). The DAL domain of the ZmDAL6 protein was used to represent plant DAL domains. The codons harboring intron splicing sites denoted by red dashed lines were from *Pp1s121_135V6*.*4* (the upper ones) and *ZmDAL6* (the basal ones).

Putative genes or gene fragments homologous to *DAL* genes were identified in lower plants to identify the origin of *DAL* genes in higher plants by lowering the HMMER search threshold (E-value of full sequence <0.01). Peptidase S8 propeptide/proteinase inhibitor I9 domain of subtilisins were identified as putative homologs of DAL proteins in *P*. *patens* and *S*. *moellendorffii* but not in *C*. *reinhardtii* (Supplementary Table S4). The proteinase inhibitor I9 domain is the propeptide of the serine peptidase family S8A (subtilisin family) and is responsible for the modulation of folding and activity of these proenzymes^32^. In addition to the protein similarity of the inhibitor I9 domain and the DAL domain, inhibitor I9 domain-encoding genes or gene fragments have conserved gene structure with *DAL* genes, including the 2-1-1 intron phase pattern and the 98-bp exon (Figs 5 and S7), which suggests that *DAL* genes probably originated from inhibitor I9 domain-encoding DNA sequences. The combination of inhibitor I9 domain-encoded exons and other exons, such as RRM-encoding exons, could be responsible for the appearance of *DAL* genes and *ORRM1-like* genes in higher plants.

### Functional divergence evaluation between plant *DAL* subfamilies

As reported previously, *Arabidopsis DAL* genes play inequable roles in RNA editing for different RNA sites by binding diverse DYW-PPR proteins^27^. To investigate the different functional constraints between these members, we conducted a maximum likelihood test of functional divergence using DIVERGE v3.0^33^. The unrooted NJ tree was generated with complete amino acid sequences of plant DAL proteins excluding those of *P*. *abies* and *V*. *vinifera* (Supplementary Fig. S8). Two types of functional divergence (type I and type II) between gene clusters of the *DAL* gene family in flowering plants were examined. The theta (*θ*) ML values were calculated, and the results demonstrated that the coefficients of type I (*θ*_I_) for 6 of 10 pairwise comparisons between DAL subfamilies were significantly greater than zero (Bonferroni corrected *P* < 0.05), and only one pairwise subfamily comparisons showed significant divergence with the coefficients of type II (*θ*_II_) test (Bonferroni corrected *P* < 0.05) (Table 1). Functional divergence-related sites were identified based on the posterior probabilities with a cut-off value of 0.85, and most were located in the DAL domain (Supplementary Fig. S9). These observations indicate that there were significant site-specific shifted selective constraints on most members of the *DAL* gene family. Furthermore, we also observed that the values of *θ*_I_ were much larger than the estimates of *θ*_II_ in each pairwise comparison (Table 1), indicating that type I functional divergence predominantly contributed to the diversified evolution of plant *DAL* genes. In addition, we checked the functional divergence of intragroup members of the *DAL* genes, such as Sub.Ia vs. Sub.Ib (Supplementary Fig. S8), but there was no significant functional divergence in any intragroup comparison (Supplementary Table S5), suggesting that intragroup *DAL* genes might play similar conserved roles in different plant lineages.

**Table 1:** Functional divergence between the subfamilies of *DAL* genes in the NJ tree based on complete protein alignment.

Comparison	*θ*	*θ*_*SE*_	z-Score^a^	*P*-value^b^
Type I (Gu99)	*θ*_I_			
Sub.I vs. Sub.II	0.4310	0.1568	2.7487	0.0060
Sub.I vs. Sub.III	0.6736	0.2139	3.1491	0.0016*
Sub.I vs. Sub.IV	0.4384	0.1494	2.9344	0.0033
Sub.I vs. Sub.V	0.7440	0.2355	3.1592	0.0016*
Sub.II vs. Sub.III	0.6224	0.1525	4.0813	<0.0001*
Sub.II vs. Sub.IV	0.5178	0.1380	3.7521	0.0002*
Sub.II vs. Sub.V	0.6480	0.1467	4.4172	<0.0001*
Sub.III vs. Sub.IV	0.7984	0.1773	4.5031	<0.0001*
Sub.III vs. Sub.V	0.3728	0.1894	1.9683	0.0490
Sub.IV vs. Sub.V	0.1832	0.1271	1.4414	0.1495
Type II	*θ*_II_			
Sub.I vs. Sub.II	0.1571	0.1500	1.0473	0.2950
Sub.I vs. Sub.III	0.2367	0.1063	2.2267	0.0260
Sub.I vs. Sub.IV	0.3228	0.1010	3.1960	0.0014*
Sub.I vs. Sub.V	0.4335	0.1465	2.9590	0.0031
Sub.II vs. Sub.III	0.1600	0.1537	1.0410	0.2979
Sub.II vs. Sub.IV	0.2349	0.1484	1.5829	0.1134
Sub.II vs. Sub.V	0.3862	0.1841	2.0978	0.0359
Sub.III vs. Sub.IV	0.1085	0.1189	0.9125	0.3615
Sub.III vs. Sub.V	0.0954	0.1931	0.4940	0.6213
Sub.IV vs. Sub.V	−0.1484	0.1937	−0.7661	0.4436

^a^z-Score is the ratio of ThetaML (*θ*) to SE Theta (*θ*_*SE*_).

^b^ *P*-value is evaluated based on the normal distribution test of the z-score.

*Bonferroni corrected *P* < 0.05.

### GC content of *DAL* genes in monocots and dicots

**Figure 6:**
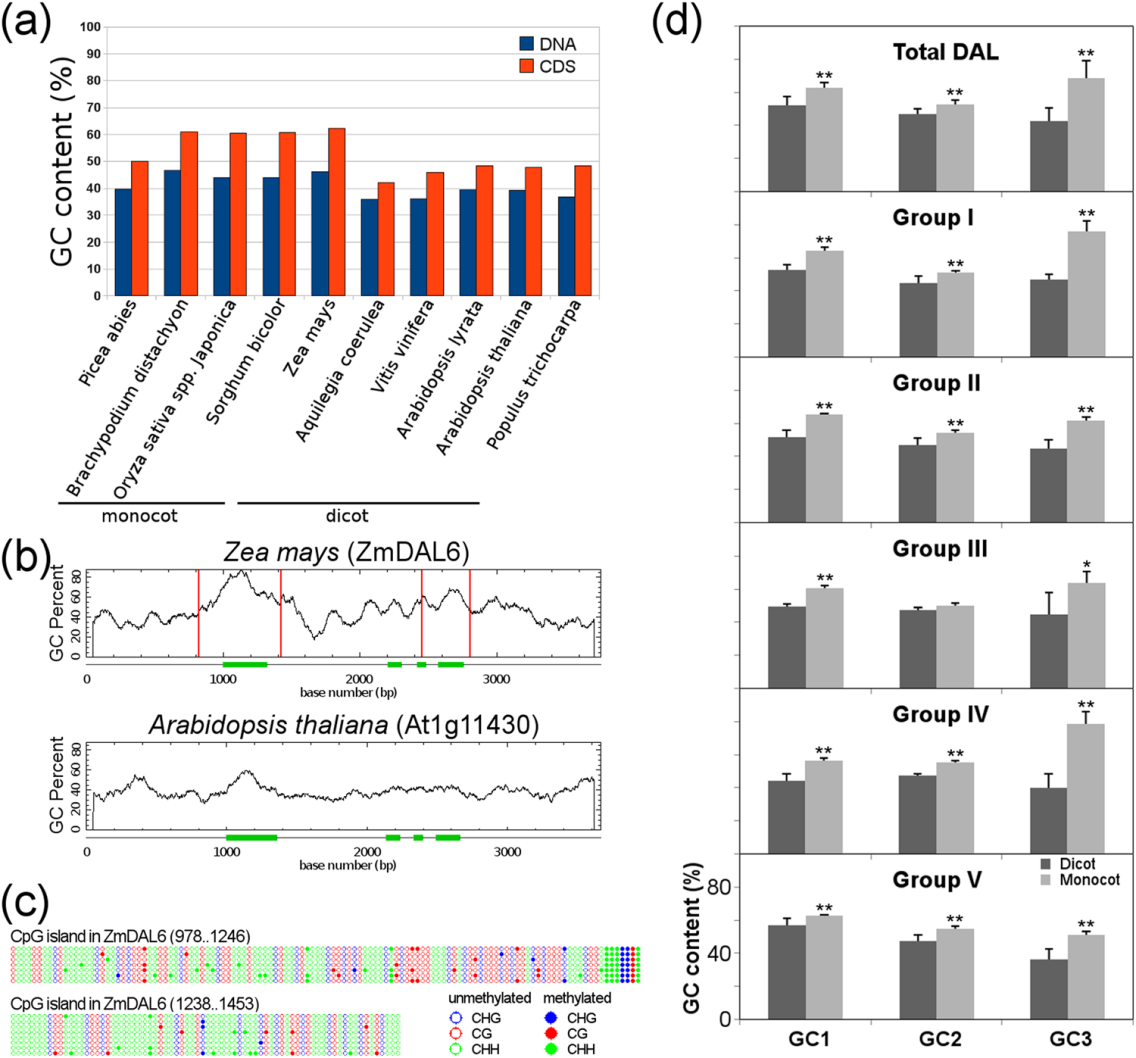
GC content and DNA methylation analysis of *DAL* genes in higher plants. (**a**,**b**) GC content and CpG islands of plant *DAL* genes were identified and displayed using CpGplot of EMBOSS (http://www.ebi.ac.uk/Tools/emboss/) with a window size of 100 bp and the following set options: Observed/Expected ratio >0.60, Percent C+ Percent G >50.00 and Length >200. The DNA sequences of the *DAL* genes used here contain 1000 bp before the start codon and 1000 bp after the stop codon. Two CpG islands within the 607-bp (nucleotides 817–1423) and 359-bp (nucleotides 817–1423) regions (817–1423) were observed and delimited by two close red lines, respectively. (**c**) The DNA methylation of the 607-bp CpG island was analyzed using bisulfite sequencing with the primers listed in Supplementary Table S2, and the data were displayed using Kismeth software (http://katahdin.mssm.edu/kismeth/revpage.pl). (**d**) Codon base composition of *DAL* genes in flowering plants. The significant differences of codon base composition between dicot and monocot *DAL* genes were statistically analyzed according to Student’s t-test. *P < 0.05; **P < 0.01. GC1, the GC contents at the first base of one codon; GC2, the GC contents at the second base; GC3, the GC contents at the third base.

The GC content, an important genomic feature, plays a critical role in determining the physical properties of DNA molecules and genome regulation by providing substrates for DNA methylation^34^. The base composition analysis of plant *DAL* genes revealed that the *DAL* genes of monocots (grass) have a higher GC content than those of dicots (Fig. 6a). To investigate DNA methylation of the GC-rich *DAL* genes, CpG islands of *ZmDAL6* and *At1g11430* as representatives were predicted, and there were two CpG island regions identified in *ZmDAL6* but not in *At1g11430* (Fig. 6b). We analyzed the DNA methylation of the first CpG island of *ZmDAL6*, which was located at the exon 1-harboring region, using bisulfite sequencing and observed that 7 cytosines within a 19-bp region (nucleotides 1,222–1,240 of the *ZmDAL6* DNA sequence in Fig. 6c) were methylated, including three CHH sites, two CHG sites and one CG site (Fig. 6c). In the 607-bp CpG island, however, most cytosines were not substantially modified by DNA methylation. Moreover, the codon base composition of the *DAL* genes was analyzed, and the result showed that the GC content of each base of one codon in monocot *DAL* genes was higher than that of dicot *DAL* genes (Student t-test, *P* < 0.01). For each group, only the codon second base (GC2) of group III *DAL* genes showed no significant difference in GC content between dicots and monocots. The largest difference of GC content was found in the codon third base (GC3) of groups I and IV between dicots and monocots (Fig. 6d), and in these two groups, more than 75% of the monocot *DAL* GC3 bases were guanine or cytosine nucleotides. Given that no significant functional divergence was observed between intragroup *DAL* genes, we inferred that the higher GC content of *DAL* genes in monocots could be caused by GC-biased gene conversion because the base composition of *DAL* genes was consistent with the high GC content of monocot genomes^34–36^.

### Expression analysis of *ZmDAL* genes by RT-PCR

Given the important roles of *DAL* genes in plant organelle RNA editing, the preferential expression patterns of *ZmDAL* genes were analyzed. To analyze the expression pattern of *ZmDAL* genes, a NimbleGen maize microarray data (ZM37)^37^ was performed for 60 tissues representing 11 major organ systems and various developmental stages of the B73 maize inbred line using *ZmDAL* probes. The median log signal values for all 7 *ZmDAL* genes were extracted. Four *ZmDAL* genes (*ZmDAL2*, *ZmDAL3*, *ZmDAL4*, and *ZmDAL5*) showed a constitutive expression pattern in 60 different tissues, with a CV value <5% (Supplementary Fig. S10). *ZmDAL1* had a much higher expression level in the leaves than in other tissues, while *ZmDAL6* showed a lower expression level in the roots and first internode compared with that in other tissues or organs. The predominant expression levels of *ZmDAL7* were observed in the developing seed, embryo and endosperm. Of these *DAL* genes, *ZmDAL1* and *ZmDAL6* were predicted to localize to chloroplasts and were preferentially expressed in the leaves, despite different expression patterns of the two genes (Supplementary Fig. S10). The expression pattern was similar for paralogous genes (*ZmDAL3* and *ZmDAL4*), indicating they were formed by segmental duplication and retained their function (Supplementary Fig. S1).

**Figure 7:**
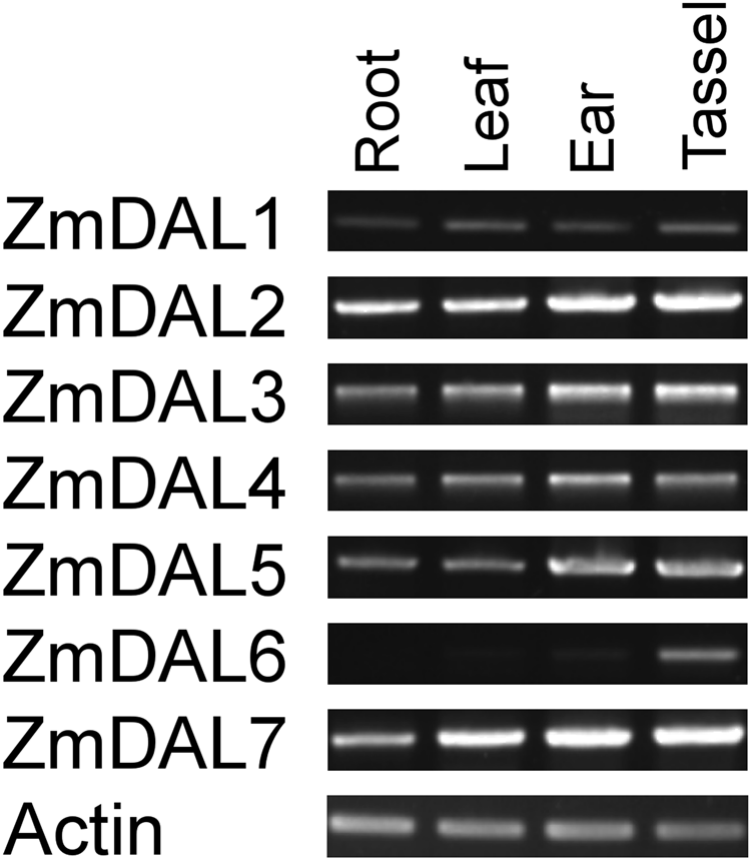
RT-PCR analysis of maize *DAL* genes in the four different tissues of maize. The total RNA of four tissues including seedling roots, seedling leaves, 5-cm immature ears and non-emerged immature tassels was isolated and used to perform the semi-quantitative RT-PCR of *ZmDAL* genes. *Actin1* was used for internal controls to normalize the RNA contents in each sample. Primers used are shown in Supplementary Table S2.

To confirm the organ-specific expression of *ZmDAL* genes shown by microarray data, RT-PCR was performed with total RNA isolated from the roots, leaves, ears, and immature tassels. The RT-PCR analysis revealed that the results for *ZmDAL2*, *ZmDAL3*, *ZmDAL4*, and *ZmDAL5* match with the DNA chip data but that the other *ZmDAL* genes do not (Fig. 7). However, the expression levels of *ZmDAL2*, *ZmDAL3*, *ZmDAL4*, and *ZmDAL5* were abundant in the ears and tassels, where more biological energy from mitochondria is required^38^. *ZmDAL1* was expressed little in the four tissues. *ZmDAL7* showed higher expression levels in the leaves, ears, and tassels in comparison to the roots. *ZmDAL6* was predominately expressed in tassels but showed little or no expression in roots, leaves and ears, according to the RT-PCR analysis (Fig. 7).

## Discussion

RNA editing of a single nucleotide, such as C-to-U and A-to-I substitution, requires *trans*-factors to recognize the nucleotide to be edited and remove the amino group. In mammals, A-to-I RNA editing is catalyzed by a family of enzymes called adenosine deaminases that act on RNA (ADARs)^39^, while C-to-U editing of apolipoprotein B mRNAs is performed by the zinc-dependent RNA-editing enzyme apolipoprotein B editing catalytic subunit 1 (APOBEC-1), which interacts with APOBEC-1 complementation factor (ACF) for site-specific editing^40^. In plant organelles, C-to-U RNA editing is mediated by site-specific DYW-PPR proteins and by several other nuclear-encoded factors, including DAL proteins, PPO1^21^ and OZ1^22^. However, the precise roles of each component of the plant RNA editosome in the complicated editing machinery are not yet well known. In this study, we performed a comprehensive analysis of *DAL* genes in plants and uncovered their seed plant-specific distribution, origin and the potential role of functional divergence.

Using a custom-built HMM file derived from multiple DAL domain alignments, we screened a plant annotation database for putative *DAL* genes and found that *DAL* genes were specific to seed plants. The absence of *DAL* genes in lower plants, in which there are thousands of organelle RNA-edited sites^41, 42^, indicates that the RNA editosome differs between higher and lower plants^2, 16, 17^. Given that the presence of *DYW-PPR* genes in plants is associated with C-to-U RNA editing events^42^ and that DAL proteins interact with PPR proteins^15^, *PPR* genes may not be necessary for the emergence of *DAL* genes in plants (Fig. 3), although DAL proteins can interact with *P*. *patens* PPR proteins^43^. Furthermore, the homologs of OZ1, ORRM and PPO1 proteins, which have been proven to interact with DAL proteins, were found in *P*. *patens* and *S*. *moellendorffii* ^15^ (Fig. 3), and in particular, putative PPO1 proteins in *P*. *patens* (*Pp1s28_304V6*.*1*) and *S*. *moellendorffii* (*Sm_82264*) all have the 22-aa regions that are essential for their interaction with DAL proteins^21^, suggesting that these additional RNA editing factors are also not required for the evolution/function of *DAL* genes, although there is no evidence for the roles of these proteins in RNA editing in lower plants. However, we cannot exclude the possibility that unidentified alternative genes aside from those of the *DAL* gene family may play similar roles in RNA editing in lower plants. In addition to the regulation of RNA editing via bridging multiple subunits in the RNA editosome, DAL proteins probably have other functions required in higher plants.

When we searched DAL proteins with the dal.hmm information as a query, propeptide inhibitor I9 domains of plant subtilisins were found to show high similarity to part of the DAL domain. In addition, the inhibitor I9 domain-encoding genes and *DAL* genes have a conserved gene structure, including the intron phases 2, 1, and 1 and the 98-bp exon (Supplementary Fig. S7), and *inhibitor I9* genes were present prior to the divergence of seed plants from ferns (Fig. 3). Therefore, it is inferred that the *DAL* genes originated from *inhibitor I9* genes by combining I9 domain-encoding exons with another unidentified sequence via exon shuffling. Additional evidence for this hypothesis stems from the fact that the *ORRM1-like* genes encode two tandem DAL domains followed by an RRM domain at the C terminus^28^, which could have arisen from the combination of two I9 domain-encoding exons and an extra RRM domain-encoding sequence. In addition to the protein sequence similarity and conserved gene structure between the *DAL* genes and I9 domain-encoding genes, they also have similar molecular function, as the DAL domain mediates the protein-protein interaction of DAL proteins with other RNA editing factors^15^, while the I9 domain inhibits proenzymes by hiding substrate-binding domains^32^.

Plant *DAL* genes were assigned to five distinct subfamilies based on their phylogenetic relationships (Fig. 4). In *Arabidopsis*, *DAL*/*RIP* genes play nonequivalent roles in RNA editing. The major factor is *RIP1* (*At3g15000*), which belongs to group II and controls 75% of RNA-edited sites in mitochondria and 20% of RNA-edited sites in chloroplasts^27^. *RIP2* (*At2g33430*), *RIP3* (*At3g06790*), *RIP9* (*At1g11430*) and *RIP8* (*At4g20020*) have moderate effects on RNA editing and are in groups I, III, IV and V, respectively (Fig. 4). As minor genes, *RIP4* (*At5g44780*), *RIP5* (*At1g32580*), *RIP6* (*At2g35240*) and *RIP7* (*At1g72530*) each shares clades with the above major or moderate factors, suggesting that these *DAL* genes have recently duplicated and have evolved to only control few RNA editing sites. We analyzed the functional divergence between DAL groups to identify the sites distinguishing the different DAL members. Five groups resulted in 10 pairwise comparisons; of these comparisons, 6 showed significant type I functional divergence, and one showed significant type II functional divergence (Table 1). In addition, we analyzed the functional divergence between intragroup members. These intragroup *DAL* genes were divided into two subgroups: a dicot subgroup (a) and a monocot subgroup (b) (Supplementary Fig. S8). No significant functional divergence was observed between intragroup members of *DAL* genes, although the GC contents of *DAL* genes in monocots and dicots were different. The sites involved in functional divergence between DAL groups were predominantly localized to the DAL domains, and relaxed selection on these sites would serve to increase the complexity for determination of RNA editing because DAL proteins act on RNA editing in the form of heterodimers in addition to homodimers. Also, these sites probably account for the interaction of each DAL with different PPR proteins in the RNA editosome. Therefore, it is understandable that different dimers formed with homogenous or heterogeneous DAL proteins, which confer RNA editing to corresponding sites, have increased the diversity of RNA editing regulation in higher plants. However, further studies on the biochemical character of DAL proteins and the crystal structure of the RNA editosome are required to parse the roles of DAL proteins with the functionally diverged sites. In addition, the putative effects of DAL proteins on other RNA processing events in addition to RNA editing should be further investigated.

## Materials and Methods

### Identification and sequence analysis of putative *DAG-like* genes in plants

Known *MORF*/*RIP* genes *At4g20020*, *At2g33430*, *At3g06790*, *At5g44780*, *At1g32580*, *At2g35240*, *At1g72530*, *At3g15000*, and *At1g11430* from *A*. *thaliana* ^16, 17, 26, 44^ and *DAG* (NCBI Protein ID: Q38732) from *A*. *majus* ^25^ were used to query the maize filtered gene set (ZmB73_5b_FGS_translations.fasta downloaded from www.maizesequence.org) using a local BLASTP program with an E-value <1e-10 and a bit score >100. ZmDAL protein sequences were analyzed using ExPASy tools available at http://us.expasy.org/tools/. Multiple sequence alignments of ZmDAL proteins and the above known DAL proteins were performed using ClustalW (http://www.ebi.ac.uk/Tools/msa/clustalw2/)^45^. To mine the conserved domain, the alignment results (Supplementary Data File S1) were used to build a protein HMM file, dubbed dal.hmm (Supplementary Data File S2) by the hmmbuild program in HMMER 3.0 package^31^.

To investigate the evolution of *DAL* genes in the plant kingdom, the dal.hmm information was used as a query to search the genome annotation data of the following representative species from Phytozome v8.0 (http://www.phytozome.net/), except those of *P*. *abies*, which were from ConGenIE (http://congenie.org/), in HMMER 3.0 package: *C*. *reinhardtii*, *P*. *patens*, *S*. *moellendorffii*, *P*. *abies*, *B*. *distachyon*, *O*. *sativa* Japonica, *S*. *bicolor*, *A*. *formosa*, *V*. *vinifera*, *A*. *lyrata* and *P*. *trichocarpa*. Protein hits with an e-value <1e-10 and sequence score of “best 1 domain” >100 were collected. The homologs of the *OZ1*, *PPO1* and *ORRM* genes in the above plants were identified using a local BLASTP program with the protein sequences of known *Arabidopsis* OZ1, PPO1, ORRM2, ORRM3 and ORRM4 proteins as queries^15^.

The PFAM (http://pfam.sanger.ac.uk/) and INTERPRO (http://www.ebi.ac.uk/interpro/) databases were screened to detect known motifs in ZmDAL proteins and the DAL proteins of other plants. The MEME program (http://meme.nbcr.net/meme/cgi-bin/meme.cgi) was used to investigate the putative conserved motifs among these ZmDAL proteins with the following parameters: length between 10 and 50 aa, maximum number of motifs to find = 5, and one per sequence. To obtain the intact conserved DAL domain, different limits for length of each motif were taken that were between 100 and 120 aa.

### Gene structures of plant *DAL* genes

The DNA and transcript sequences of *ZmDAL* genes obtained from the maize sequence annotation database MaizeGDB (http://www.maizegdb.org/) were used to analyze the gene structures of *ZmDAL* genes. Several *ZmDAL* genes had more than one gene model annotated in MaizeGDB. To confirm the putative alternative splicing transcripts, transcript-specific primers (Supplementary Table S2) were designed to amplify corresponding DNA isolated from B73 seedlings and cDNA derived from B73 seedling RNA. Gene structures producing validated transcripts were drawn and displayed using the online GSDraw program of the PIECE server (http://wheat.pw.usda.gov/piece/GSDraw.php)^46^. Conserved DAL domains were also displayed using the GSDraw program. The gene structures of *DAL* genes from other plant species were obtained from the Phytozome v8.0 annotation database (http://www.phytozome.net/) and displayed using the GSDraw program^46^.

### Subcellular localization prediction of ZmDAL proteins

Two *in silico* programs, Predotar^47^ and TargetP^48^, were used to predict the putative organelle localization of ZmDAL proteins. The maize organelle proteomics database (PPDB, http://ppdb.tc.cornell.edu/)^49^ was screened to detect the accumulation of ZmDAL proteins.

### Phylogenetic dendrogram

The multiple sequence alignment analysis of conserved DAL domains collected from 79 DAL proteins identified in maize and in other higher plants was carried out using MUSCLE v3.8.31^50^, and the resulting alignment was used to build the NJ distance phylogenetic tree using MEGA v5.0^51^ by applying the Poisson substitution model, 1000 bootstrap samples, and pairwise deletion for gaps/missing data. The tree was displayed using FigTree v1.4.0 (http://tree.bio.ed.ac.uk/software/figtree/).

### Functional divergence analysis

To investigate the functional alteration of duplicated *DAL* genes in plants, the GU99 method within DIVERGR v3.0^33^ was used to calculate the coefficients of type I and type II functional divergence (*θ*_I_ and *θ*_II_, respectively) between two groups after gene duplication and to predict functionally divergent amino acids based on their different evolutionary rates. Within two duplicated groups of a gene family, type I functional divergence helps identify the relaxation of functional constraint in one group relative to that of another, while type II identifies shifting patterns of functional constraint.

### GC content and DNA methylation analysis with bisulfite sequencing

The entire DNA sequences of plant *DAL* genes together with 1 kb of upstream and downstream flanking sequences were used for calculation of GC content and prediction of CpG islands in the EMBOSS CpGplot online server (http://www.ebi.ac.uk/Tools/seqstats/emboss_cpgplot/). To confirm the DNA methylation of *ZmDAL* DNA sequences, leaf DNA of B73 seedlings was isolated and treated with bisulfate using the EpiTect^®^ bisulfite kit (QIAGEN, USA) according to the manufacturer’s instructions. The primers for detection of DNA methylation were designed using MethPrimer (http://www.urogene.org/methprimer/)^52^ and modified using Primer3web (http://primer3.ut.ee/). PCR products were cloned into a pGEM-T vector (Promega, USA) and subsequently sequenced using an ABI3730 DNA sequencer (Shanghai Sunny Bio., China). DNA methylation states via bisulfite sequencing were analyzed and displayed using Kismeth software (http://katahdin.mssm.edu/kismeth/revpage.pl)^53^.

### Expression analysis of *ZmDAL* genes in different tissues

To investigate the spatiotemporal expression patterns of *ZmDAL* genes, the log2-transformed and RMA-normalized data for *ZmDAL* genes were downloaded from PLEXdb (http://www.plexdb.org/)^54^, and cluster analysis of these expression data were performed using Cluster v3.0^55^ with the hierarchic method. A heat map was produced using Java TreeView v1.1.5^56^. The coefficient of variation (CV) was calculated according to the following equation to estimate the expression variation of each *ZmDAL* gene in different tissues: CV = sd/mean, where sd indicates the standard deviation of a gene in different tissues and the mean represents the average expression level.

### Semi-quantitative reverse transcription PCR (semiq-RT-PCR)

Total RNA was isolated from different tissues of the B73 inbred lines, including seedling roots, leaves, 5-cm ears and immature tassels, using the Trizol^®^ reagent (Invitrogen, USA) according to the manufacturer’s protocol. First-strand cDNA was produced from 1 μg of total RNA (25 μl reaction volume) using M-MLV reverse transcriptase (Invitrogen, USA) at 37 °C for 1 h. All gene-specific primers were designed as shown in Supplementary Data Table S2. Specific primers for the maize *Actin1* gene (GenBank ID: NM_001155179) were used as an internal control. Reactions were performed with Taq Polymerase (Dalian Takara Biotechnology, China) on a Bio-Rad Thermal Cycler (Bio-Rad, USA) using the following procedure: 5 min at 94 °C to start; 32 cycles of 30 s at 94 °C, 30 s at 58 °C and 1 min at 72 °C; and a final extension step of 72 °C for 10 min to complete the reaction, and the *Actin1* transcript was amplified with 28 PCR cycles. Each PCR pattern was performed in triplicate, mixtures without a template were employed as negative controls, and the maize *Actin1* amplicon served as an internal control for each gene investigated.

## Electronic supplementary material

Supplementary Information
