# Supplementary material for: Functional divergence and origin of the DAG-like gene family in plants
Source: Sci Rep. 2017 Jul 18;7:5688. doi: 10.1038/s41598-017-05961-2 (PMC5515838; doi:10.1038/s41598-017-05961-2)
Supplement: Supplementary file 1 — Supplementary Information [file 41598_2017_5961_MOESM1_ESM.pdf]

**Functional divergence and origin of the *DAG-like* gene family in plants**

Meijie Luo[1,4], Manjun Cai[2,4], Jianhua Zhang[2], Yurong Li[2], Ruyang Zhang[1], Wei Song[1], Ke Zhang[2], Hailin Xiao[2,3], Bing Yue[2], Yonglian Zheng[2], Yanxin Zhao[1,2,]*, Jiuran Zhao[1,]* & Fazhan Qiu[2,]*

[1] Beijing Key Laboratory of Maize DNA Fingerprinting and Molecular Breeding, Maize Research Center, Beijing Academy of Agricultural and Forestry Sciences, Beijing 100097, China.

[2] National Key Laboratory of Crop Genetic Improvement, Huazhong Agricultural University, Wuhan 430070, China.

[3] Present address: Life Science and Technology Center, China National Seed Group Co., Ltd., Wuhan 430075, China.

[4] These authors contributed equally to this work.

*Correspondence and requests for materials should be addressed to Jr. Z (email: maizezhao@126.com), Yx.Z. (email: rentlang2003@163.com) or F.Q. (email: qiufazhan@mail.hzau.edu.cn)

**Supplementary figure, table and data file legends**

**Supplementary Figures**

**Figure S1.** Chromosomal location and gene duplication of maize *DAL* genes.

(a) All of 7 maize *DAL* genes mapped onto maize chromosomes based on their annotation data from MaizeGDB (http://www.maizegdb.org/). The *ZmDAL3* and *ZmDAL4* linked by red line were involved in chromosomal segment duplication identified in PLAZA v2.5 (http://bioinformatics.psb.ugent.be/plaza/-versions/plaza_v2_5/). (b) Inter-chromosomal syntenic blocks were analyzed with SyMAP v4.0 (http://symapdb.org/) and *ZmDAL* genes were marked out of maize genome circle based on their relative positions on maize chromosomes.

**Figure S2**. Correction of *ZmDAL1* gene model.

(a) Multiple sequence alignment of ZmDAL1, GRMZM2G175447, Sobic.006G204100 and LOC_Os04g51280 proteins was carried out in Clustal Omega program (http://www.ebi.ac.uk/Tools/msa/-clustalo/). The latter two genes are the orthologs of *ZmDAL1* in sorghum and rice, respectively. (b) Multiple sequence alignment of the cDNA sequences of *ZmDAL1*, *GRMZM2G175447* and 5'-UTR of *GRMZM2G156227*. (c) BAC-contigs (AC191302.2-Contig72 and AC191302.2-Contig70) harboring separated fractions of *ZmDAL1* caused by genomic gaps were rearranged to produce the intact *ZmDAL1* gene.

**Figure S3.** Confirmation gene structures of *ZmDAL* genes by RT-PCR.

DNA and cDNA of B73 seedling were used as templates to amplify the *ZmDAL* genes with the primers listed in Supplementary Table S2. The PCR products were analyzed in 1% agarose gel. *ZmDAL1** was the putative intact gene annotation of *ZmDAL1*. There were no specific PCR products obtained for DNA sequences of *ZmDAL5* and *ZmDAL6* with the given primers (Supplementary Table S2). The red star

denotes the PCR product of maize *Actin1*.

**Figure S4.** MEME motifs of ZmDAL proteins.

(a-b) Two MEME motifs were obtained with 10 aa < the length of motif < 100 aa as a set. (c-d) The 114-aa MEME motif was found with 100 aa < the length of motif < 150, and matched the DAL domain.

**Figure S5.** Gene structures and conserved motifs of *ORRM1-like* genes.

GSDraw (http://wheat.pw.usda.gov/piece/GSDraw.php)[46] was used to construct the gene structures of plant *ORRM1-like* genes. Conserved MEME motifs were shown in Figure1. TargetP (http://www.cbs.dtu.dk/-services/TargetP/)[48] and (/) Predotar (https://urgi.versailles.inra.fr/predotar/predotar.html)[47] programs were used to predict subcellular localization of ORRM1-like proteins. C, chloroplast; M, mitochondria; -, none.

**Figure S6.** Gene structures and DAL domains of plant *DAL* genes.

Gene structures of plant *DAL* genes were generated using GSDS 2.0 (http://gsds.cbi.pku.edu.cn/) by aligning CDS and DNA sequences obtained from Phytozome v9.0 (https://phytozome.jgi.doe.gov/pz/-portal.html). DAL domains in plant DAL proteins were displayed by DOG 2.0 (http://ibs.biocuckoo.org/).

**Figure S7.** Gene structures and inhibitor I9 domains of plant peptidase S8A genes.

(a) Gene structures of plant peptidase S8A genes were generated using GSDraw (http://wheat.pw.-usda.gov/piece/GSDraw.php)[46] with their DNA and CDS sequences download from Phytozome v9.0 (https://phytozome.jgi.doe.gov/pz/portal.html). The TargetP (http://www.cbs.dtu.dk/services/TargetP/)[48] was used to predict the subcellular location of peptidase S8A proteins. S, secretory pathway signal peptide in proteins of interest. The exons encoding Inhibitor I9 regions homologous to DAL domains were labeled in red. (b) Known domains of peptidase S8A proteins were identified by searching the Pfam database (http://pfam.xfam.org/) and they were displayed with DOG2.0 (http://ibs.biocuckoo.org/). The regions of the Inhibitor I9 domain homologous to DAL domains were marked by the red bars below them.

**Figure S8.** The NJ phylogenetic tree for functional divergence analysis of plant DAL proteins.

Complete protein sequences of plant *DAL* genes were aligned using MUSCLE v3.8.31[50] and the alignment was used to generate the NJ tree of plant *DAL* genes with MEGA v5.0[51]. Roman numbers on the right stand for subfamilies or groups of plant *DAL* genes classified in Figure 4. The lower letters, a and b, at the nodes of the NJ tree represent the subclades of each group.

**Figure S9.** The posterior probabilities of the residues under functional divergence.

The posterior probabilities of the residues under type I and type II functional divergence were labeled in black with the cut-off of 0.85. The posterior probabilities of type II diverged sites shown here had been transformed with the formula, $P_{\text{type II}} = P_{\text{type II}} / (P_{\text{type II}} + 1)$. The DAL domain residues were marked by blue bar under each figure.

**Figure S10.** Gene expression profile of *ZmDAL* genes in different tissues.

The expression data of maize *DAL* genes download from PLEXdb (http://www.plexdb.org/)[54] was clustered using Cluster v3.0[55] with the hierarchic method. The heat map of *DAL* gene expression was generated by Java TreeView v1.1.5[56]. The coefficient of variation (CV) was defined as the ratio of the standard deviation to the mean a gene across all the tissues.

**Supplementary Tables**

**Table S1**. Detailed information of maize *DAL* genes and proteins.xls

**Table S2**. Primers used in this study.doc

**Table S3**. *DAL* genes in higher plants identified in this study.doc

**Table S4**. Peptidase inhibitor I9-containing subtilase proteins homologous to DAL proteins in plants.doc

**Table S5**. Functional divergence between intragroup DAL pairwise comparisons.doc

**Supplementary Data Files**

**Data File S1.** dal.hmm

**Data File S2.** The DAL domain alignment of 17 plant DAL proteins.sto

**Data File S3.** Alignment of 79 plant DAL proteins for NJ tree construction.fas

**Data File S4.** Alignment of complete protein sequences of plant *DAL* genes.fas

**Supplementary Figures**

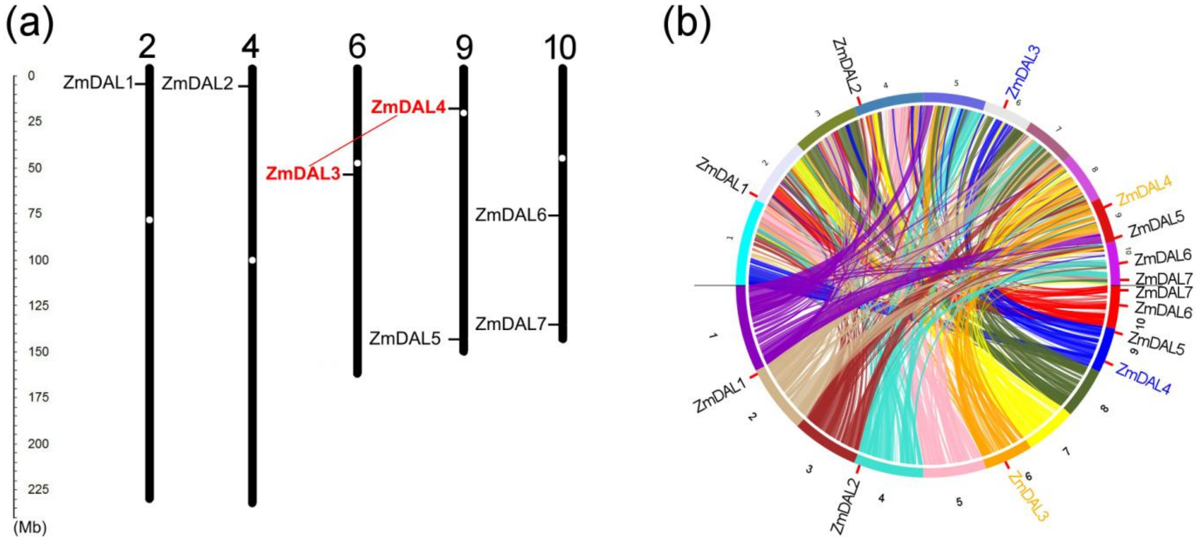


**Supplementary Figure S1**

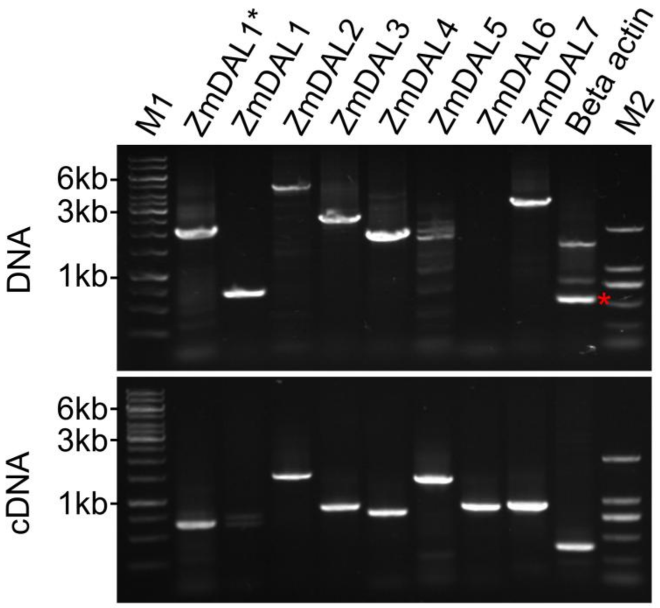


**Supplementary Figure S2**

(a)

```
LOC_Os04g51280      MATAAAAARAVAAAGRPAQGVPLSRRLTTASSSSARPLRPRGGRAAGSVRCMARRPESSYSPLRSGQGGDRAPTEMAPLFPGCDYEHWLIVMDKPGGEGATKQQMIDCYIQTLAKVVGSE
GRMZM2G175447       MATA---ARAL-VAARPARPLLPSRRL--PSSSSIRPARQR--AGVGCVRCMARRPDSTYSPLRSGQGGDRAPTEMAPLFPGCDYEHWLIVMDKPGGEGATKQQMIDCYIQTLAQVVGSE
ZmDAL1              MATA---ARAL-VAARPARPLLPSRRL--PSSSSIRPARQR--AGVGCVRCMARRPDSTYSPLRSGQGGDRAPTEMAPLFPGCDYEHWLIVMDKPGGEGATKQQMIDCYIQTLAQVVGSE
Sobic.006G204100    MATA---ARAL-VAARPARPLLPSRRL--PSSSSIRPPRQRG-GGVGSVRCMARRPDSSYSPLRSGQGGDRAPTEMAPLFPGCDYEHWLIVMDKPGGEGATKQQMIDCYIQTLAQVVGSE
                    ****   ***: .*.***: :  ****   **** ** * *   ..*.********:*:*******************************************************:*****

LOC_Os04g51280      EEAKKKIYNVSCERYFGFGCEIDEETSNKLEGLP-GVLFVLPDSYVDAENKDYGAELFVNGEIVQRSPERQRRVEPVPQRAQDRPRYSDRTRYVKRRENQAYQR
GRMZM2G175447       EEAKKRIYNVSCERYFGFGCEIDEETSNKLEGDLFGSLFK--SKYVQCDF------IFCFS------------------------------------------
ZmDAL1              EEAKKRIYNVSCERYFGFGCEIDEETSNKLEGLP-GVLFVLPDSYVDAENKDYGAELFVNGEIVQRSPERQRRVEPVPQRAQDRPRYSDRTRYVKRRENQSYQR
Sobic.006G204100    EEAKKRIYNVSCERYFGFGCEIDEETSNKLEGLP-GVLFVLPDSYVDAENKDYGAELFVNGEIVQRSPERQRRVEPVPQRAQDRPRYSDRTRYVKRRENQSYQR
                    *****:**************************   * **   ..**:.:       :*  .
```

(b)

```
GRMZM2G175447_T01         ATCTCCGATCGTCCGTGTCCCTCACCAAAAGTTCAAAACCCCATCCAATTCCAAATCCCACTTCTCGCCAGTTCACCTCCGCCTCCGCTCCTCCCCTCCCCCTCCCGTCCTGCCATGGCC
ZmDAL1                    ------------------------------------------------------ATCCCACTTCTCGCCAGTTCACCTCCGCCTCCGCTCCTCCCCTCCCCCTCCCGTCCTGCCATGGCC
GRMZM2G156227_T01(5'-UTR) ------------------------------------------------------------------------------------------------------------------------

GRMZM2G175447_T01         ACCGCAGCACGCGCCCTCGTCGCCGCACGCCCGGCACGGCCGCTGCTGCCGTCTCGGCGCCTCCCGTCCTCCTCTTCCATCCGTCCCGCGCGGCAACGTGCTGGCGTCGGGTGCGTCCGC
ZmDAL1                    ACCGCAGCACGCGCCCTCGTCGCCGCACGCCCGGCACGGCCGCTGCTGCCGTCTCGGCGCCTCCCGTCCTCCTCTTCCATCCGTCCCGCGCGGCAACGTGCTGGCGTCGGGTGCGTCCGC
GRMZM2G156227_T01(5'-UTR) ------------------------------------------------------------------------------------------------------------------------

GRMZM2G175447_T01         TGCATGGCTCGGCGGCCGGATTCCACCTATTCCCCGCTGCGTTCGGGCCAGGGCGGTGACCGTGCACCGACTGAAATGGCGCCTCTGTTCCCTGGCTGCGACTACGAGCACTGGCTCATC
ZmDAL1                    TGCATGGCTCGGCGGCCGGATTCCACCTATTCCCCGCTGCGTTCGGGCCAGGGCGGTGACCGTGCACCGACTGAAATGGCGCCTCTGTTCCCTGGCTGCGACTACGAGCACTGGCTCATC
GRMZM2G156227_T01(5'-UTR) ------------------------------------------------------------------------------------------------------------------------

GRMZM2G175447_T01         GTCATGGACAAGCCCGGGGGCGAGGGCGCCACCAAGCAGCAAATGATAGATTGTTACATCCAGACCCTTGCCCAGGTGGTAGGGAGTGAAGAGGAGGCGAAGAAGAGGATATATAATGTG
ZmDAL1                    GTCATGGACAAGCCCGGGGGCGAGGGCGCCACCAAGCAGCAAATGATAGATTGTTACATCCAGACCCTTGCCCAGGTGGTAGGGAGTGAAGAGGAGGCGAAGAAGAGGATATATAATGTG
GRMZM2G156227_T01(5'-UTR) ------------------------------------------------------------------------------------------------------------------------

GRMZM2G175447_T01         TCCTGCGAGCGCTATTTTGGATTCGGATGCGAAATTGACGAGGAGACCTCCAACAAACTCGAAGGTGACTTGTTTGGTTCCTTGTTCAAG-----------AGTAAATACGTGCAATGTG
ZmDAL1                    TCCTGCGAGCGCTATTTTGGATTCGGATGCGAAATTGACGAGGAGACCTCCAACAAACTCGAAGGCCTTCCAGGGGTTCTTTTTGTGCTTCCTGACTCTTACGTTGATGCTGAGAACAAG
GRMZM2G156227_T01(5'-UTR) ------------------------------TGCGCTAACTGTGGTTTGTGTTTGGATAAAACAGGCCTTCCAGGGGTTCTTTTTGTGCTTCCTGACTCTTACGTTGATGCTGAGAACAAG

GRMZM2G175447_T01         ATTTCATCTTCTGTTTCAGTTAATGTATTTTGCGTTGCGCTATAATGTTGATAGTTGCATAGTGTGATGGATGGCATGG-----AAAATTAAG-ATTTCTGCTTGTGCA---GAATGCCT
ZmDAL1                    GATTATGGTGCTGAGTTATTTGTGAACGGTGAAATCGTTCAGCGATCTCCAGAAAGGCAGAGAAGGGTGGAGCCAGTGCCTCAGAGAGCTCAGGATCGACCACGGTACAGTGACCGGACC
GRMZM2G156227_T01(5'-UTR) GATTATGGTGCTGAGTTATTTGTGAACGGTGAAATCGTTCAGCGATCTCCAGAAAGGCAGAGAAGGGTGGAGCCAGTGCCTCAGAGAGCTCAGGATCGACCACGGTACAGTGACCGGACC

GRMZM2G175447_T01         AGTTATGTTAAACTGTTA-----CGGTTTTGCAGTCTATTTATGATTCGGT---------------------------------------------------------------------
ZmDAL1                    CGCTATGTGAAGCGGAGGGAGAACCAATCTTACCAGAGATGATGTTTCCGTCTCTGAAGATGTAAAATGTCACGGTTTCCACAAGTGTCCAAATGCGGTACTGAAGATGCAGCAGCAACT
GRMZM2G156227_T01(5'-UTR) CGCTATGTGAAGCGGAGGGAGAACCAATCTTACCAGAGATGATGTTTCCGTCTCTGAAGATGTAAAATGTCACGGTTTCCACAAGTGTCCAAATGCGGTACTGAAGATGCAGCAGCAACT

GRMZM2G175447_T01         ------------------------------------------------------------
ZmDAL1                    CCCCACTACTCA------------------------------------------------
GRMZM2G156227_T01(5'-UTR) CCCCACTACTCATATGAGAATGGCATCCGTTCTGTTTGCCGCGCATTTTAAGCTGTATAT
```

(c)

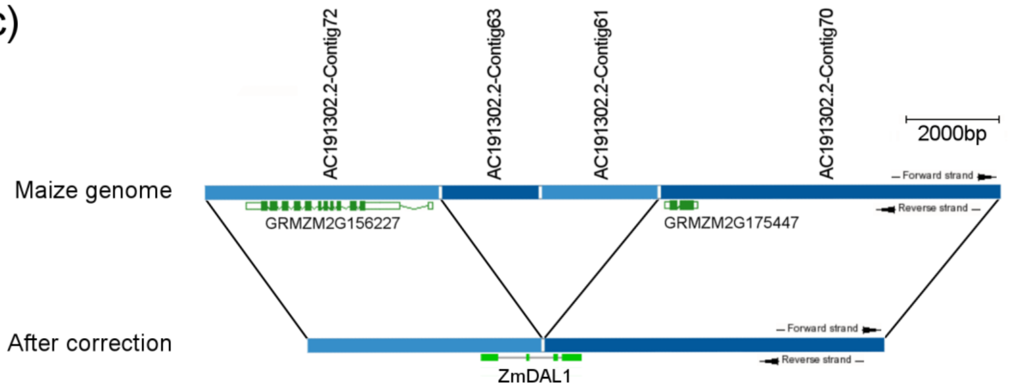


**Supplementary Figure S3**

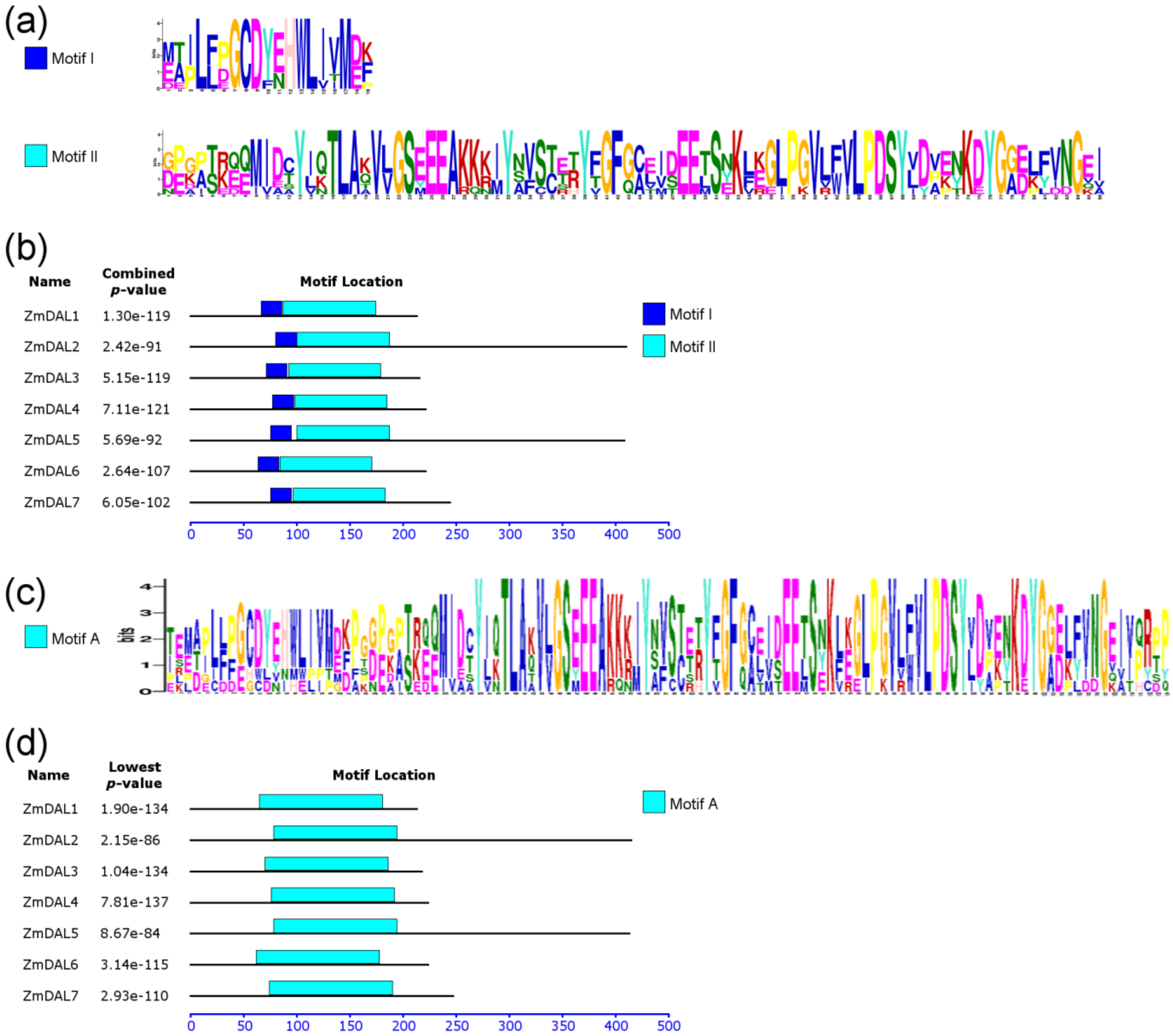


**Supplementary Figure S4**

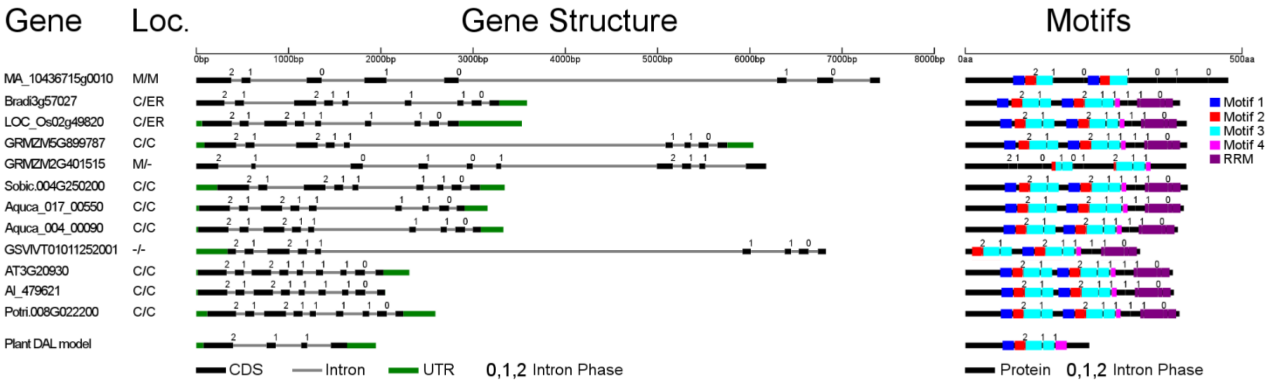


**Supplementary Figure S5**

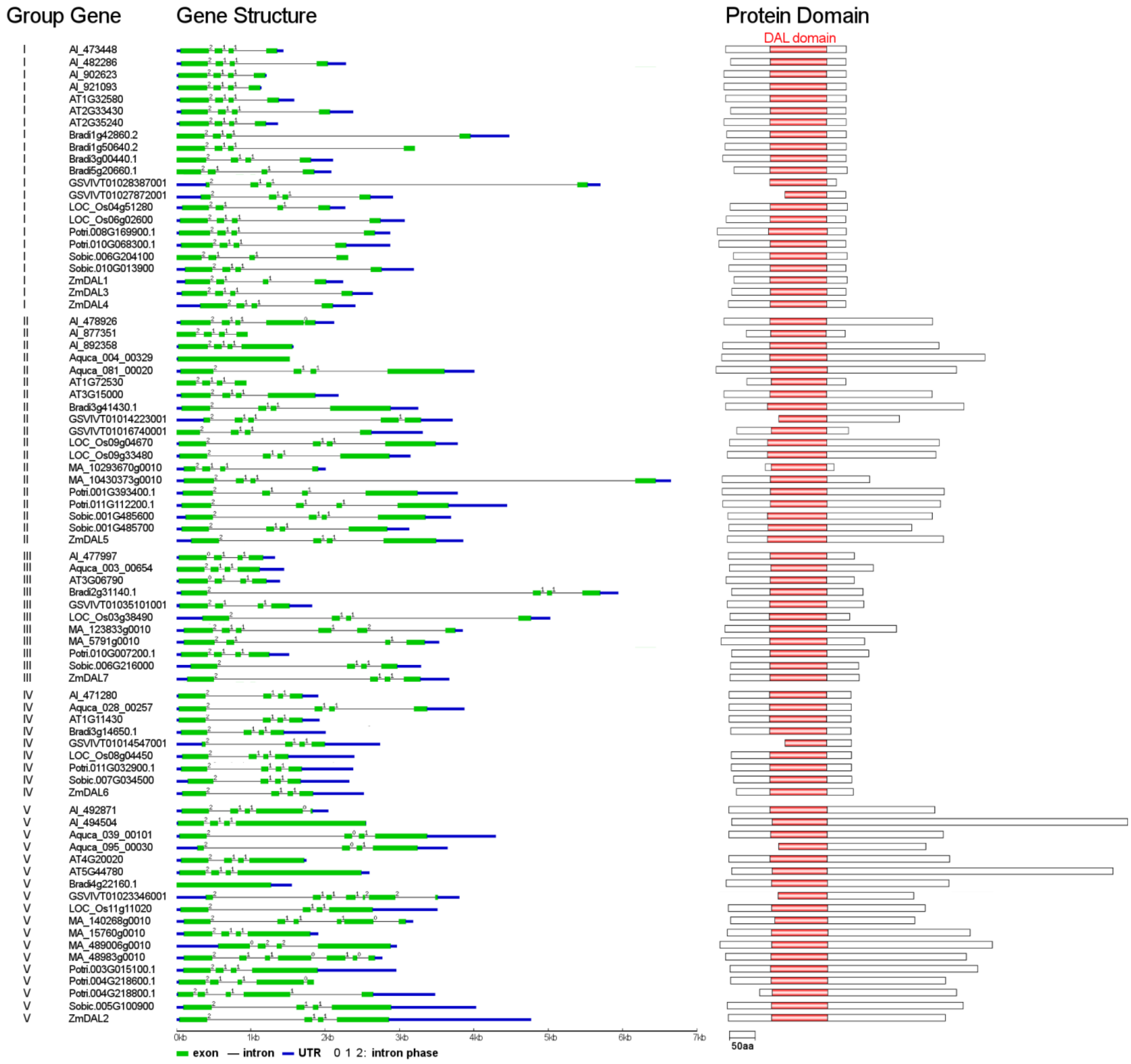


**Supplementary Figure S6**

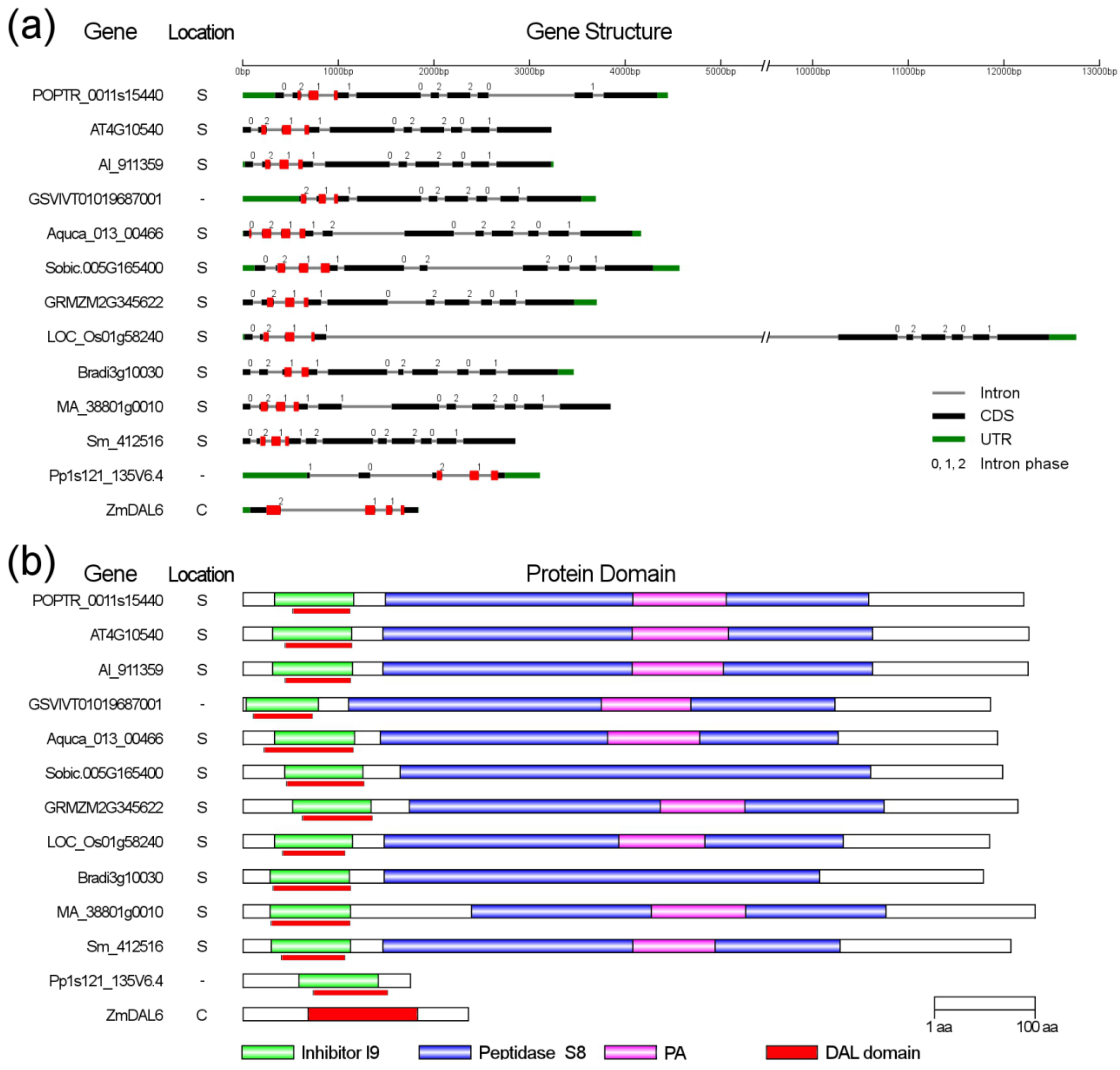


**Supplementary Figure S7**

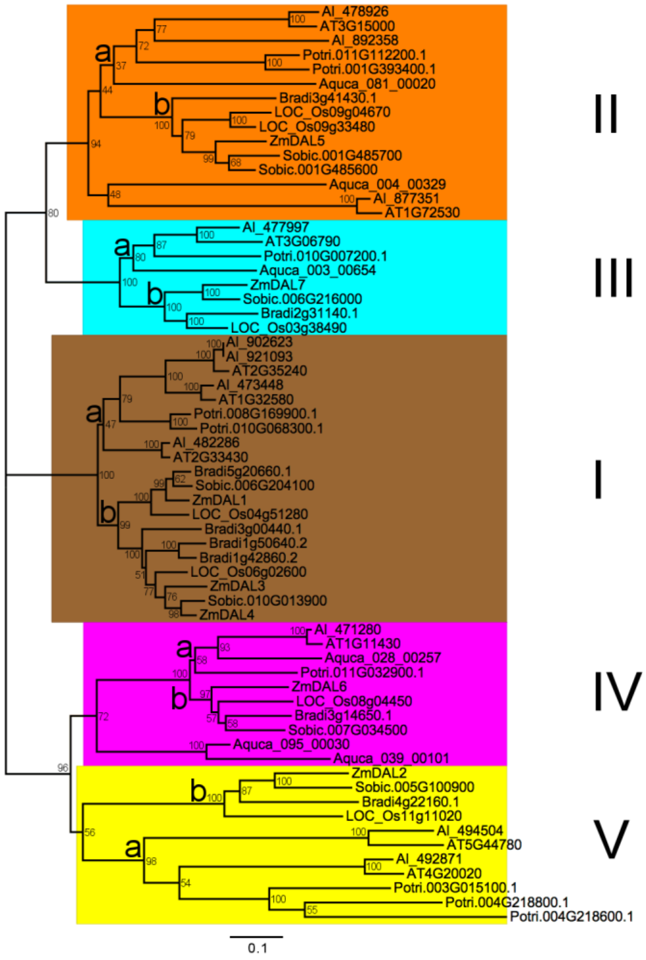


**Supplementary Figure S8**

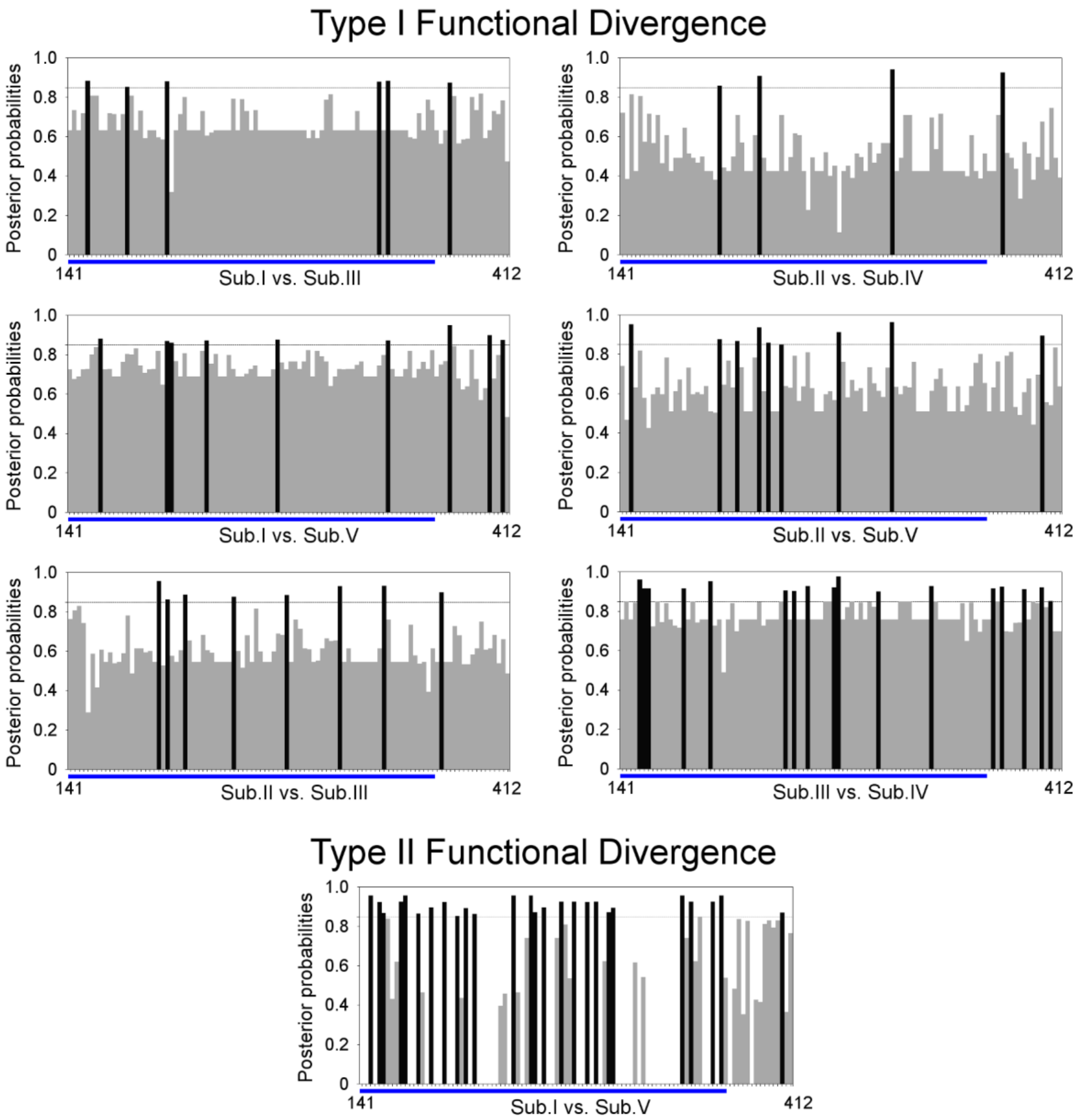


**Supplementary Figure S9**

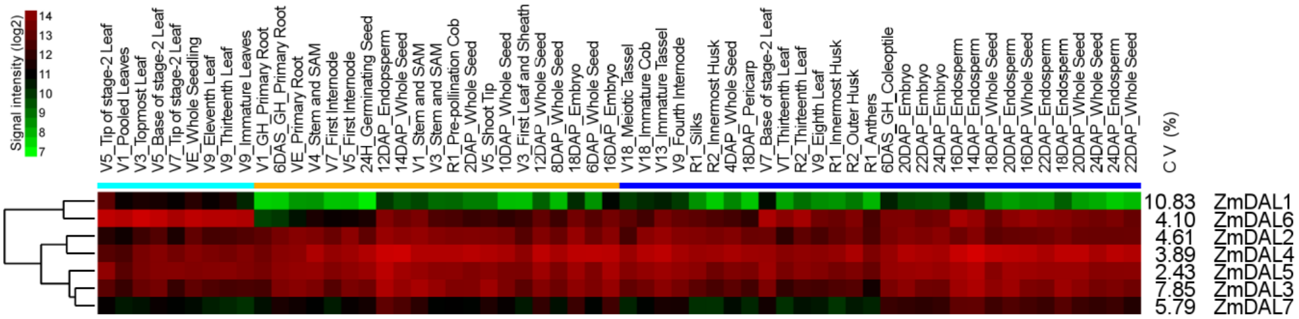


**Supplementary Figure S10**

## Supplementary Tables

## Supplemental Table S1. Detailed information of maize *DAL* genes and proteins.

| | | | | | Protein | | | *In silico* prediction | | | Best Hits in BLASTP | Colinear Orthologs in | | | |
|---|---|---|---|---|---|---|---|---|---|---|---|---|---|---|---|
| Gene Name | Gene Model | AS | Chromosomal Position | Genebank Accession (NCBI) | Length (aa) | MW (kDa) | pI | Predator | TargetP. | PPDB | Arabidopsis | *Sorghum bicolor* | *Setaria italica* | *Oryza sativa J.* | *Brachypodium distachyon* |
| *ZmDAL1* | GRMZM2G175447 | 1 | 2:12,111,695-12,112,532 | BT064978 | 215 | 24.27 | 9.06 | M | C | C | At2g33430 | Sb06g027510 | Si011034m.g | LOC_Os04g51280 | Bradi5g20660 |
| *ZmDAL2* | GRMZM2G139441 | 4 | 4:12,818,434-12,823,201 | BT042458 | 412 | 44.58 | 8.81 | M | M | | At4g20020 | Sb05g008690 | Si026454m.g | LOC_Os11g11020 | Bradi4g22160 |
| *ZmDAL3* | GRMZM5G808811 | 1 | 6:60,978,927-60,981,565 | BT018304.1 | 217 | 24.21 | 9.21 | M | M | | At2g33430 | Sb10g001390 | Si007230m.g | LOC_Os06g02600 | Bradi1g50640 |
| *ZmDAL4* | GRMZM2G383540 | 2 | 9:25,522,193-25,524,594 | EU966136.1 | 223 | 24.8 | 9.21 | M | M | | At1g32580 | Sb10g001390 | Si007230m.g | LOC_Os06g02600 | Bradi1g50640 |
| *ZmDAL5* | GRMZM2G169384 | 3 | 9:150,809,669-150,813,518 | AY104285.2 | 410 | 43.96 | 9.22 | M | M | | At3g15000 | Sb01g045460 | N.A. | N.A. | N.A. |
| *ZmDAL6* | GRMZM2G003765 | 4 | 10:83,417,551-83,420,067 | BT063172.1 | 223 | 24.61 | 9.2 | C | C | C | At1g11430 | Sb07g002970 | no gene model | LOC_Os08g04450 | Bradi3g14650 |
| *ZmDAL7* | GRMZM2G054537 | 1 | 10:142,505,503-142,509,165 | EU973085.1 | 246 | 27.27 | 9.15 | M | M | | At3g06790 | Sb06g028620 | Si010903m.g | N.A. | N.A. |

**Supplemental Table S2. Primers used in this study.**

| Primer Pairs | Sequence (5'->3') | Gene | Usage | Size of PCR products | | |
|---|---|---|---|---|---|---|
| | | | | DNA (bp) | cDNA (bp) | PCR cycles |
| 55MF | GTGGTAAAATTTTGTGTGTAATAATGGTTG | *ZmDAL6* | DNA methylation detection | 269 | - | |
| 323MR | CTCCATGACGATCAGCCAGTGGTTGTA | | | | | |
| 315MF | GTTATGGAGTTTTTTAAGGATTT | *ZmDAL6* | DNA methylation detection | 216 | - | |
| 530MR | TACCCTTACAATTTAAAACCTATTC | | | | | |
| 55F | ATCCCACTTCTCGCCAGTTCACCT | *ZmDAL1* | Determination of *ZmDAL* gene | 707 | 615 | 32 |
| 669R | TCCATGCCATCCATCACACTATGCAAC | | structure | | | |
| 186F | GTCCTCCTCTTCCATCCGTC | *ZmDAL1** | Determination of *ZmDAL* gene | -[b] | 667 | 32 |
| 852R | TGAGTAGTGGGGAGTTGCTG | | structure, and sqRT-PCR | | | |
| 22F | TCCAAAACCTTCAGCCCCACGCTA | *ZmDAL2* | Determination of *ZmDAL* gene | 4661 | 1479 | 32 |
| 1500R | GGGAGTTCCACATTCTGCGCCATT | | structure, and sqRT-PCR | | | |
| 55F | AGGGTTTTGCCACCATGGCCG | *ZmDAL3* | Determination of *ZmDAL* gene | 2469 | 825 | 32 |
| 879R | TGTGCAAACGGGCGCTAGTAACA | | structure, and sqRT-PCR | | | |
| 13F | TTCCGAATCCGTTCCACCATTGCC | *ZmDAL4* | Determination of *ZmDAL* gene | 1895 | 781 | 32 |
| 1039R | ACCACCTGGGACTTGTTGCTGT | | structure, and sqRT-PCR | | | |
| 106F | TGCGGCTTAAACCCTTATCGCCAC | *ZmDAL5* | Determination of *ZmDAL* gene | 3468 | 1411 | 32 |

| | | | | | | |
|---|---|---|---|---|---|---|
| 1516R | CGATTTATGCTAGGCTACGGCGCA | | structure, and sqRT-PCR | | | |
| 47F | ATCCCCTTGCGGCAAAACCTTG | *ZmDAL6* | Determination of *ZmDAL* gene | 2085 | 891 | 32 |
| 937R | ACACACACACACAAGCTGCCCA | | structure, and sqRT-PCR | | | |
| 28F | TTCACACTCCGGCTACCACCAT | *ZmDAL7* | Determination of *ZmDAL* gene | 3323 | 928 | 32 |
| 955R | AGCAACTGCCGGCAAAAACCA | | structure, and sqRT-PCR | | | |
| ACTIN_F | GGCCCAACTGCCGAAGCCAT | *Actin1*[a] | Control | - | 389 | 28 |
| ACTIN_R | GAGAGGGGCCTCGGTCAGCA | | | | | |

* The intact *ZmDAL1* gene model is derived from segmental sequences of AC191302.2-Contig70 and AC191302.2-Contig72 (See Supplementary Fig. S1).

[a] The maize *actin1* gene (Genebank #NM_001155179; Gene model #GRMZM2G126010) was used as an internal control for RT-PCR analysis.

**Supplementary Table S3.** ***DAL*** **genes in higher plants identified in this study.**

| Plant species | Group | Gene model | Length of protein (aa) | Subcellular location[a] |
|---|---|---|---|---|
| ***Picea abies*** **(8)** | III | *MA_123833g0010* [b] | 326 | C/C |
| | III | *MA_5791g0010* | 273 | M/M |
| | V | *MA_489006g0010* | 517 | C/- |
| | II | *MA_10430373g0010* | 280 | M/M |
| | II | *MA_10293670g0010* | 131 | -/- |
| | V | *MA_48983g0010* | 457 | C/M |
| | V | *MA_15760g0010* | 461 | C/M |
| | V | *MA_140268g0010* | 350 | M/M |
| ***Brachypodium distachyon*** **(8)** | IV | *Bradi3g14650.1* | 229 | C/C |
| | I | *Bradi5g20660.1* | 215 | C/M |
| | I | *Bradi1g50640.2* | 230 | M/M |
| | I | *Bradi1g42860.2* | 227 | M/M |
| | I | *Bradi3g00440.1* | 234 | C/M |
| | III | *Bradi2g31140.1* | 250 | M/M |
| | II | *Bradi3g41430.1* | 452 | M/M |
| | V | *Bradi4g22160.1* | 423 | M/M |
| ***Oryza sativa spp. Japonica*** **(7)** | IV | *LOC_Os08g04450* | 229 | C/C |
| | I | *LOC_Os04g51280* | 223 | C/C |
| | I | *LOC_Os06g02600* | 227 | M/M |
| | III | *LOC_Os03g38490* | 228 | M/M |
| | V | *LOC_Os11g11020* | 374 | M/M |
| | II | *LOC_Os09g04670* | 398 | M/M |
| | II | *LOC_Os09g33480* | 396 | C/M |
| ***Sorghum bicolor*** **(7)** | IV | *Sobic.007G034500* | 225 | C/C |
| | I | *Sobic.006G204100* | 216 | C/M |
| | I | *Sobic.010G013900* | 222 | M/M |
| | III | *Sobic.006G216000* | 244 | M/M |
| | II | *Sobic.001G485600* | 388 | M/M |
| | V | *Sobic.005G100900* | 448 | M/M |
| | II | *Sobic.001G485700* | 347 | M/M |

| | | | | |
|---|---|---|---|---|
| ***Aquilegia coerulea* (6)** | IV | *Aquca_028_00257* | 233 | C/C |
| | V | *Aquca_095_00030* | 280 | -/- |
| | V | *Aquca_039_00101* | 407 | M/M |
| | III | *Aquca_003_00654* | 274 | M/M |
| | II | *Aquca_081_00020* | 456 | C/C |
| | II | *Aquca_004_00329* | 499 | C/M |
| ***Vitis vinifera* (7)** | I | *GSVIVT01028387001* | 145 | -/- |
| | III | *GSVIVT01035101001* | 260 | M/M |
| | II | *GSVIVT01016740001* | 212 | M/M |
| | I | *GSVIVT01027872001* | 116 | -/- |
| | IV | *GSVIVT01014547001* | 127 | -/- |
| | II | *GSVIVT01014223001* | 229 | -/- |
| | V | *GSVIVT01023346001* | 258 | -/- |
| ***Arabidopsis lyrata* (11)** | IV | *Al_471280* [c] | 232 | C/C |
| | I | *Al_473448* | 229 | C/M |
| | I | *Al_482286* | 219 | C/C |
| | I | *Al_902623* | 232 | C/M |
| | I | *Al_921093* | 232 | C/M |
| | III | *Al_477997* | 240 | C/M |
| | II | *Al_478926* | 396 | C/M |
| | II | *Al_892358* | 410 | C/M |
| | V | *Al_492871* | 391 | M/M |
| | II | *Al_877351* | 188 | M/M |
| | V | *Al_494504* | 751 | M/M |
| ***Arabidopsis thaliana* (9)** | IV | *AT1G11430* | 232 | C/C |
| | I | *AT1G32580* | 229 | C/M |
| | II | *AT1G72530* | 188 | M/M |
| | I | *AT2G33430* | 219 | M/C |
| | I | *AT2G35240* | 232 | C/M |
| | III | *AT3G06790* | 244 | C/M |
| | II | *AT3G15000* | 395 | C/C |
| | V | *AT4G20020* | 419 | M/M |
| | V | *AT5G44780* | 723 | M/M |

| | | | | |
|---|---|---|---|---|
| ***Populus trichocarpa* (9)** | IV | *Potri.011G032900.1* | 229 | C/C |
| | I | *Potri.008G169900.1* | 245 | M/M |
| | I | *Potri.010G068300.1* | 241 | M/M |
| | III | *Potri.010G007200.1* | 261 | M/M |
| | II | *Potri.011G112200.1* | 413 | C/M |
| | II | *Potri.001G393400.1* | 421 | C/C |
| | V | *Potri.003G015100.1* | 470 | M/M |
| | V | *Potri.004G218800.1* | 374 | -/- |
| | V | *Potri.004G218600.1* | 408 | M/M |

[a] Subcellular localization of plant DAL proteins were predicted within TargetP (http://www.-cbs.dtu.dk/services/TargetP/)[48] and (/) Predotar (https://urgi.versailles.inra.fr/predotar/predotar.html)[47] programs. C, chloroplast; M, mitochondria; -, none.

[b] All of DAL protein sequences were from Phytozome v9.1 (www.phytozome.net/) except those of *Picea abies* which were from ConGenIE database (http://congenie.org/).

[c] The names of *Arabidopsis lyrata DAL* genes were modified with the '*Al_*' as a prefix.

**Supplementary Table S4. Peptidase inhibitor I9-containing subtilase proteins homologous to DAL proteins in plants.**

| Species | Gene[a] | Domain[b] |
|---|---|---|
| *Physcomitrella patens* (1) | *Pp1s121_135V6* | I9 |
| *Selaginella moellendorffii* (6) | *Sm_412516* | I9; S8; PA |
| | *Sm_234928* | I9; S8; PA |
| | *Sm_437460* | I9; S8; PA |
| | *Sm_407291* | I9; S8; PA |
| | *Sm_235873* | I9; S8; PA |
| | *Sm_125066* | I9; S8; PA |
| *Picea abies* (9) | *MA_10430416g0010*† | I9; S8; PA |
| | *MA_58143g0010*† | I9; S8; PA |
| | *MA_10436904g0020* | I9; S8 |
| | *MA_47270g0010* | I9; S8; PA |
| | *MA_38801g0010* | I9; S8; PA |
| | *MA_161971g0010* | I9; S8; PA |
| | *MA_90908g0010*† | I9 |
| | *MA_106581g0010* | I9; S8; PA |
| | *MA_358621g0010* | I9 |
| *Brachypodium distachyon* (4) | *Bradi3g10030* | I9; S8 |
| | *Bradi2g24260* | I9 |
| | *Bradi3g20580* | I9; S8; PA |
| | *Bradi3g10037* | I9; S8 |
| *Oryza sativa* spp. japonica (9) | *LOC_Os04g51290* | - |
| | *LOC_Os02g17000* | I9; S8; PA |
| | *LOC_Os01g58240* | I9; S8; PA |
| | *LOC_Os02g17090* | I9; S8; PA |
| | *LOC_Os05g35740* | I9 |
| | *LOC_Os02g17080* | I9; S8; PA |
| | *LOC_Os04g03796* | I9; S8; PA |
| | *LOC_Os01g58280* | I9; S8; PA |
| | *LOC_Os01g58290* | I9; S8; PA |

| | | |
|---|---|---|
| *Zea mays* L. (4) | *GRMZM2G353990_P01* | - |
| | *GRMZM2G367107_P01* | - |
| | *GRMZM2G345622_P01* | I9; S8; PA |
| | *GRMZM2G057159_P04* | I9; S8; PA |
| *Sorghum bicolor* (3) | *Sobic.004G123700.1.p* | I9; S8 |
| | *Sobic.005G165400.1.p* | I9; S8 |
| | *Sobic.003G323700.1.p* | I9; S8; PA |
| *Aquilegia coerulea* (2) | *Aquca_013_00466.1* | I9; S8; PA |
| | *Aquca_013_00542.1* | I9; S8; PA |
| *Vitis vinifera* (5) | *GSVIVT01019686001*‡ | I9; S8; PA; Ank_2 |
| | *GSVIVT01038641001*‡ | I9; S8; PA; mTERF |
| | *GSVIVT01012792001* | I9 |
| | *GSVIVT01024195001* | I9; S8; PA |
| | *GSVIVT01019687001* | I9; S8; PA |
| *Arabidopsis lyrata* (9) | *Al_911359* | I9; S8; PA |
| | *Al_863678* | I9; S8; PA |
| | *Al_329472* | I9; S8; PA; ComA |
| | *Al_489899* | I9; S8; PA |
| | *Al_881603* | I9; S8; PA |
| | *Al_890879* | I9; S8; PA |
| | *Al_350386* | I9; S8; PA |
| | *Al_316165* | I9 |
| | *Al_473476* | I9; S8; PA |
| *Arabidopsis thaliana* (7) | *AT4G10550* | I9; S8; PA |
| | *AT4G10510* | I9; S8; PA |
| | *AT4G10540* | I9; S8; PA |
| | *AT1G32950* | I9; S8; PA |
| | *AT1G32960* | I9; S8; PA |
| | *AT1G32940* | I9; S8; PA |
| | *AT1G71950* | I9; S8; PA |
| *Populus trichocarpa* (5) | *Potri.013G112800* | I9 |
| | *Potri.019G083300* | I9 |
| | *Potri.011G151200* | I9; S8; PA |

| | |
|---|---|
| *Potri.011G050000* | I9; S8; PA |
| *Potri.011G050200* | I9; S8; PA |

[a] All of I9-containing proteins shown here homologous to the DAL domain were identified using HMMER package[31] with the dal.hmm as a query and the E-value < 1e-4. The proteins were derived from Phytozome v9.0 (http://www.phytozome.net) except for those of *Picea abies* which were from ConGenIE (http://congenie.org/). The gene names were modified with the species abbreviation as prefixes, such as SM for *Selaginella moellendorffii* and Al for *Arabidopsis lyrata*.

[b] The conserved domains or motifs in these proteins were investigated by screening the Pfam database (http://pfam.sanger.ac.uk). I9, Inhibitor_I9 (PF05922); S8, Peptidase_S8 (Subtilase family, PF00082); PA, Protease associated (PF02225); Ank_2 (Ankyrin repeats, PF12796); mTERF (PF02536); ComA, (2R)-phospho-3-sulfolactate synthase (PF02679).

† The gene models encoding I9-containing proteins span the genomic gaps.

‡ Putatively mis-annotated gene models.

**Supplementary Table S5. Functional divergence between intragroup DAL pairwise comparisons.**

| Comparison | $\theta$ | $\theta_{SE}$ | z-Score[a] | *P*-value[b] |
|---|---|---|---|---|
| Type I (Gu99) | $\theta_{I}$ | | | |
| Sub.Ia vs. Sub.Ib | -1.124 | -[c] | - | - |
| Sub.IIa vs. Sub.IIb | 0.0010 | 0.0224 | 0.0446 | 0.9644 |
| Sub.IIIa vs. Sub.IIIb | 0.5208 | 0.3113 | 1.6730 | 0.0943 |
| Sub.IVa vs. Sub.IVb | 0.0010 | 0.0224 | 0.0446 | 0.9644 |
| Sub.Va vs. Sub.Vb | 0.0010 | 0.0224 | 0.0446 | 0.9644 |
| Type II | $\theta_{II}$ | | | |
| Sub.Ia vs. Sub.Ib | 0.0018 | 0.0685 | 0.0263 | 0.9790 |
| Sub.IIa vs. Sub.IIb | -0.0771 | 0.0843 | -0.9146 | 0.3604 |
| Sub.IIIa vs. Sub.IIIb | -0.0146 | 0.0778 | -0.1877 | 0.8511 |
| Sub.IVa vs. Sub.IVb | 0.0109 | 0.0365 | 0.2986 | 0.7652 |
| Sub.Va vs. Sub.Vb | 0.0291 | 0.1519 | 0.1916 | 0.8481 |

[a] z-score is the ratio of ThetaML ($\theta$) to SE Theta ($\theta_{SE}$).

[b] *P*-value is evaluated based on the normal distribution test of absolute value of z-score.

[c] $\theta_{SE}$, z-score and *P*-value were not shown because the value of $\theta_{I}$ between Subfamily Ia and Subfamily Ib was much less than zero.

## Supplementary Data Files

## Supplemental Data File S1 dal.hmm

HMMER3/b [3.0 | March 2010]

NAME dal

LENG 109

ALPH amino

RF no

CS no

MAP yes

DATE Fri Aug 23 00:02:52 2013

NSEQ 17

EFFN 0.643311

CKSUM 970760748

STATS LOCAL MSV -9.5656 0.71563

STATS LOCAL VITERBI -10.3500 0.71563

STATS LOCAL FORWARD -4.0145 0.71563

HMM A C D E F G H I K L

M N P Q R S T V W Y

m->m m->i m->d i->m i->i d->m d->d

COMPO 2.59942 4.07575 2.87211 2.59917 3.24770 2.84350 3.74137 2.77958 2.69365 2.48927

3.62061 3.15359 3.40286 3.15557 3.15037 2.68996 2.86299 2.58033 4.41656 3.13926

2.68618 4.42225 2.77519 2.73123 3.46354 2.40513 3.72494 3.29354 2.67741 2.69355

4.24690 2.90346 2.73739 3.18146 2.89801 2.37887 2.77519 2.98518 4.58477 3.61503

0.07072 3.97375 3.00620 0.61958 0.77255 0.00000 *

1 2.59154 4.72742 2.64151 2.00998 3.94966 3.38710 3.63607 3.29295 2.46115 2.95442

3.01156 2.93664 3.80376 2.80471 2.91331 2.64051 2.82929 2.77438 5.25635 3.93018 1 - -

2.68618 4.42225 2.77519 2.73123 3.46354 2.40513 3.72494 3.29354 2.67741 2.69355

4.24690 2.90346 2.73739 3.18146 2.89801 2.37887 2.77519 2.98518 4.58477 3.61503

0.02954 3.93257 4.65492 0.61958 0.77255 0.52573 0.89435

2 2.05877 4.43010 3.05601 2.52066 3.96994 3.20526 3.81572 3.30964 2.68795 3.00889

3.86525 3.07027 3.16762 3.03114 3.08313 2.56386 1.94537 2.96615 5.30852 4.03325 2 - -

2.68618 4.42225 2.77519 2.73123 3.46354 2.40513 3.72494 3.29354 2.67741 2.69355

4.24690 2.90346 2.73739 3.18146 2.89801 2.37887 2.77519 2.98518 4.58477 3.61503

0.02954 3.93257 4.65492 0.61958 0.77255 0.52573 0.89435

3 2.52277 4.23152 3.55896 3.04415 3.48654 3.45986 3.94269 1.94457 2.96768 2.47851

3.44444 3.38194 2.89866 3.28893 3.28527 2.54116 2.81411 2.25813 4.98337 3.75092 3 - -

2.68618 4.42225 2.77519 2.73123 3.46354 2.40513 3.72494 3.29354 2.67741 2.69355

4.24690 2.90346 2.73739 3.18146 2.89801 2.37887 2.77519 2.98518 4.58477 3.61503

0.02954 3.93257 4.65492 0.61958 0.77255 0.52573 0.89435

4 3.21575 4.62920 4.52340 4.00262 3.05586 4.33657 4.63741 2.18201 3.77070 0.92128

2.63403 4.29837 4.60107 4.03903 3.94991 3.70815 3.45740 2.27495 5.08692 3.91298 4 - -
2.68618 4.42225 2.77519 2.73123 3.46354 2.40513 3.72494 3.29354 2.67741 2.69355
4.24690 2.90346 2.73739 3.18146 2.89801 2.37887 2.77519 2.98518 4.58477 3.61503
0.02954 3.93257 4.65492 0.61958 0.77255 0.52573 0.89435
5 3.10591 4.49268 4.53861 4.00124 2.03380 4.25533 4.39105 2.21082 3.87619 1.26426
2.96646 4.21618 4.52402 4.02673 4.03232 3.58859 3.33889 2.05915 4.76230 3.41213 5 - -
2.68618 4.42225 2.77519 2.73123 3.46354 2.40513 3.72494 3.29354 2.67741 2.69355
4.24690 2.90346 2.73739 3.18146 2.89801 2.37887 2.77519 2.98518 4.58477 3.61503
0.02954 3.93257 4.65492 0.61958 0.77255 0.46156 0.99506
6 2.79695 5.26941 1.91786 1.74160 4.57896 3.25196 3.69144 4.06221 2.60564 3.58288
4.39921 2.73060 2.45920 2.84078 3.14673 2.72574 3.06604 3.65503 5.75491 4.30802 6 - -
2.68618 4.42225 2.77519 2.73123 3.46354 2.40513 3.72494 3.29354 2.67741 2.69355
4.24690 2.90346 2.73739 3.18146 2.89801 2.37887 2.77519 2.98518 4.58477 3.61503
0.02833 3.97375 4.69610 0.61958 0.77255 0.48576 0.95510
7 2.79479 4.55947 3.48924 3.40188 4.54097 0.58863 4.51533 4.16583 3.59688 3.80708
4.78415 3.65362 3.95383 3.91308 3.82119 2.96972 3.28392 3.67461 5.60873 4.64652 7 - -
2.68618 4.42225 2.77519 2.73123 3.46354 2.40513 3.72494 3.29354 2.67741 2.69355
4.24690 2.90346 2.73739 3.18146 2.89801 2.37887 2.77519 2.98518 4.58477 3.61503
0.02833 3.97375 4.69610 0.61958 0.77255 0.48576 0.95510
8 2.70552 0.75628 4.24727 4.01051 3.97936 3.30647 4.63539 3.14066 3.83821 3.05129
4.17615 3.97054 4.02136 4.18021 3.94441 2.97726 3.18086 2.87825 5.37338 4.26615 8 - -
2.68618 4.42225 2.77519 2.73123 3.46354 2.40513 3.72494 3.29354 2.67741 2.69355
4.24690 2.90346 2.73739 3.18146 2.89801 2.37887 2.77519 2.98518 4.58477 3.61503
0.02833 3.97375 4.69610 0.61958 0.77255 0.48576 0.95510
9 3.10050 5.24170 0.78299 2.40253 4.63282 3.28927 4.02330 4.24449 3.12706 3.83512
4.81618 2.96369 3.94931 3.26316 3.65644 3.05197 3.44151 3.87315 5.78156 4.48997 9 - -
2.68618 4.42225 2.77519 2.73123 3.46354 2.40513 3.72494 3.29354 2.67741 2.69355
4.24690 2.90346 2.73739 3.18146 2.89801 2.37887 2.77519 2.98518 4.58477 3.61503
0.02833 3.97375 4.69610 0.61958 0.77255 0.48576 0.95510
10 3.54204 4.82674 4.48796 4.15033 1.54680 4.30274 3.46262 3.26153 4.00714 2.61411
3.87012 3.99289 4.62409 4.03006 4.08365 3.66846 3.76868 3.18040 3.62236 1.05597 10 - -
2.68618 4.42225 2.77519 2.73123 3.46354 2.40513 3.72494 3.29354 2.67741 2.69355
4.24690 2.90346 2.73739 3.18146 2.89801 2.37887 2.77519 2.98518 4.58477 3.61503
0.02833 3.97375 4.69610 0.61958 0.77255 0.48576 0.95510
11 2.82136 5.24866 2.41136 1.62015 4.56079 3.29910 3.67326 4.02674 2.26313 3.54004
4.36320 2.09452 3.83122 2.82069 2.92095 2.75761 3.07652 3.63596 5.69375 4.28748 11 - -
2.68618 4.42225 2.77519 2.73123 3.46354 2.40513 3.72494 3.29354 2.67741 2.69355
4.24690 2.90346 2.73739 3.18146 2.89801 2.37887 2.77519 2.98518 4.58477 3.61503
0.02833 3.97375 4.69610 0.61958 0.77255 0.48576 0.95510
12 3.11032 4.90855 3.22884 2.99973 3.35891 3.53592 0.94564 3.82887 2.84017 3.30981
4.36125 3.40466 4.09442 3.36749 3.09943 3.18030 3.41744 3.56211 4.82249 3.30708 12 - -
2.68618 4.42225 2.77519 2.73123 3.46354 2.40513 3.72494 3.29354 2.67741 2.69355
4.24690 2.90346 2.73739 3.18146 2.89801 2.37887 2.77519 2.98518 4.58477 3.61503
0.02833 3.97375 4.69610 0.61958 0.77255 0.48576 0.95510

13 3.50824 4.84616 4.24887 3.98048 2.77581 3.84597 4.02899 3.53683 3.70084 2.91393
4.16584 4.10924 4.37231 4.08323 3.81115 3.71094 3.81392 3.42996 0.70365 2.77273 13 - -
2.68618 4.42225 2.77519 2.73123 3.46354 2.40513 3.72494 3.29354 2.67741 2.69355
4.24690 2.90346 2.73739 3.18146 2.89801 2.37887 2.77519 2.98518 4.58477 3.61503
0.02833 3.97375 4.69610 0.61958 0.77255 0.48576 0.95510
14 3.26996 4.70264 4.35181 3.99288 3.17634 4.11019 4.61688 2.38676 3.75962 0.75990
3.16905 4.25850 4.52863 4.10370 3.91882 3.69398 3.55989 2.45024 5.09160 3.83805 14 - -
2.68618 4.42225 2.77519 2.73123 3.46354 2.40513 3.72494 3.29354 2.67741 2.69355
4.24690 2.90346 2.73739 3.18146 2.89801 2.37887 2.77519 2.98518 4.58477 3.61503
0.02833 3.97375 4.69610 0.61958 0.77255 0.48576 0.95510
15 3.07918 4.41326 4.73039 4.23969 3.51307 4.38709 4.95930 1.17049 4.11692 2.00915
3.32712 4.46609 4.71230 4.38705 4.31816 3.77735 3.34912 1.38373 5.48379 4.27922 15 - -
2.68618 4.42225 2.77519 2.73123 3.46354 2.40513 3.72494 3.29354 2.67741 2.69355
4.24690 2.90346 2.73739 3.18146 2.89801 2.37887 2.77519 2.98518 4.58477 3.61503
0.02833 3.97375 4.69610 0.61958 0.77255 0.48576 0.95510
16 2.83669 4.30029 4.21015 3.66885 3.47666 3.99480 4.44083 1.85246 3.56269 2.02360
3.34604 3.95639 4.36776 3.85349 3.82098 3.32046 2.48561 1.36400 5.20736 4.00120 16 - -
2.68618 4.42225 2.77519 2.73123 3.46354 2.40513 3.72494 3.29354 2.67741 2.69355
4.24690 2.90346 2.73739 3.18146 2.89801 2.37887 2.77519 2.98518 4.58477 3.61503
0.02833 3.97375 4.69610 0.61958 0.77255 0.48576 0.95510
17 2.98205 4.49933 4.26806 3.78030 3.21065 4.00533 4.49323 2.24867 3.57408 1.72493
1.47445 4.04546 4.40612 3.88965 3.78386 3.38836 3.26863 2.11549 5.12211 3.92641 17 - -
2.68618 4.42225 2.77519 2.73123 3.46354 2.40513 3.72494 3.29354 2.67741 2.69355
4.24690 2.90346 2.73739 3.18146 2.89801 2.37887 2.77519 2.98518 4.58477 3.61503
0.02833 3.97375 4.69610 0.61958 0.77255 0.48576 0.95510
18 2.83944 5.39330 1.80387 1.61545 4.68566 3.24583 3.68841 4.18860 2.36643 3.67677
4.48931 2.48474 3.81045 2.83442 3.15538 2.74507 3.10556 3.76297 5.83172 4.35893 18 - -
2.68618 4.42225 2.77519 2.73123 3.46354 2.40513 3.72494 3.29354 2.67741 2.69355
4.24690 2.90346 2.73739 3.18146 2.89801 2.37887 2.77519 2.98518 4.58477 3.61503
0.02833 3.97375 4.69610 0.61958 0.77255 0.48576 0.95510
19 2.61412 4.47148 3.23300 2.73560 2.37997 3.47690 3.73899 3.01240 2.26798 2.67456
3.59503 3.17132 2.85419 3.02663 2.99801 2.76329 2.86306 2.77446 4.94310 3.60791 19 - -
2.68618 4.42225 2.77519 2.73123 3.46354 2.40513 3.72494 3.29354 2.67741 2.69355
4.24690 2.90346 2.73739 3.18146 2.89801 2.37887 2.77519 2.98518 4.58477 3.61503
0.02833 3.97375 4.69610 0.61958 0.77255 0.48576 0.95510
20 2.32600 4.28713 3.17545 2.91859 4.21163 3.02337 4.07115 3.59349 2.97188 3.29874
4.16579 3.19697 1.48574 3.32251 3.29931 2.27713 2.56059 3.13263 5.55445 4.29699 20 - -
2.68618 4.42225 2.77519 2.73123 3.46354 2.40513 3.72494 3.29354 2.67741 2.69355
4.24690 2.90346 2.73739 3.18146 2.89801 2.37887 2.77519 2.98518 4.58477 3.61503
0.08578 3.97375 2.75822 0.61958 0.77255 0.48576 0.95510
21 2.63283 4.91236 2.76508 2.34698 4.24668 2.70095 3.60499 3.67753 1.99934 3.23364
4.04055 2.63849 3.35908 2.60326 2.75786 2.63413 2.76999 3.31509 5.42535 4.07954 21 - -
2.68618 4.42225 2.77519 2.73123 3.46354 2.40513 3.72494 3.29354 2.67741 2.69355
4.24690 2.90346 2.73739 3.18146 2.89801 2.37887 2.77519 2.98518 4.58477 3.61503

0.02998 3.91795 4.64030 0.61958 0.77255 0.45377 1.00849
22 2.83806 5.26757 1.69128 2.02465 4.63704 1.93170 3.75434 4.12641 2.73736 3.66264
4.50346 2.72557 3.81999 2.92195 3.29890 2.76718 3.13433 3.71229 5.83814 4.38464 22 - -
2.68618 4.42225 2.77519 2.73123 3.46354 2.40513 3.72494 3.29354 2.67741 2.69355
4.24690 2.90346 2.73739 3.18146 2.89801 2.37887 2.77519 2.98518 4.58477 3.61503
0.02833 3.97375 4.69610 0.61958 0.77255 0.48576 0.95510
23 2.65641 5.01956 2.47397 1.87620 4.29106 3.32421 3.61444 3.74844 2.43321 3.29909
4.09699 2.63995 2.81797 2.75139 2.92581 2.63376 2.89935 3.37775 5.49125 3.48563 23 - -
2.68618 4.42225 2.77519 2.73123 3.46354 2.40513 3.72494 3.29354 2.67741 2.69355
4.24690 2.90346 2.73739 3.18146 2.89801 2.37887 2.77519 2.98518 4.58477 3.61503
0.02833 3.97375 4.69610 0.61958 0.77255 0.48576 0.95510
24 2.28019 4.69111 2.96876 2.45943 3.99875 2.94715 3.64462 3.40614 2.24131 2.82957
3.84754 2.78693 3.12585 2.80954 2.88233 2.60849 2.66874 3.08051 5.27546 3.95767 24 - -
2.68618 4.42225 2.77519 2.73123 3.46354 2.40513 3.72494 3.29354 2.67741 2.69355
4.24690 2.90346 2.73739 3.18146 2.89801 2.37887 2.77519 2.98518 4.58477 3.61503
0.11715 3.97375 2.38876 0.61958 0.77255 0.48576 0.95510
25 1.81232 4.20746 3.19668 2.88999 4.14032 2.97100 4.01908 3.51084 2.94655 3.21646
4.06570 3.15888 1.98524 3.26748 3.29065 2.18992 2.69489 3.04941 5.48423 4.23920 25 - -
2.68621 4.42228 2.77513 2.73126 3.46357 2.40515 3.72497 3.29341 2.67736 2.69358
4.24692 2.90338 2.73733 3.18149 2.89803 2.37890 2.77522 2.98521 4.58480 3.61506
0.11971 2.27433 4.60988 0.77594 0.61669 0.43870 1.03528
26 2.38930 4.35967 3.14901 2.79514 4.06834 3.12512 3.92977 3.45289 2.79751 3.13460
3.99222 3.14435 3.75604 2.97734 3.15397 1.88925 1.72838 3.05752 5.40641 4.14049 30 - -
2.68618 4.42225 2.77519 2.73123 3.46354 2.40513 3.72494 3.29354 2.67741 2.69355
4.24690 2.90346 2.73739 3.18146 2.89801 2.37887 2.77519 2.98518 4.58477 3.61503
0.02833 3.97375 4.69610 0.61958 0.77255 0.48576 0.95510
27 2.82544 5.07053 3.08547 2.34481 4.44393 3.51130 3.58587 3.83296 1.80357 3.32541
4.15053 3.01475 3.43981 2.71558 1.71775 2.81495 3.02631 3.48650 5.43890 4.17620 31 - -
2.68618 4.42225 2.77519 2.73123 3.46354 2.40513 3.72494 3.29354 2.67741 2.69355
4.24690 2.90346 2.73739 3.18146 2.89801 2.37887 2.77519 2.98518 4.58477 3.61503
0.02833 3.97375 4.69610 0.61958 0.77255 0.48576 0.95510
28 2.81397 5.35420 1.99326 1.65859 4.64832 3.25500 3.67473 4.14833 2.57254 3.63813
4.44395 2.45857 3.80508 2.29501 3.11273 2.72702 3.07601 3.72507 5.79243 4.32931 32 - -
2.68618 4.42225 2.77519 2.73123 3.46354 2.40513 3.72494 3.29354 2.67741 2.69355
4.24690 2.90346 2.73739 3.18146 2.89801 2.37887 2.77519 2.98518 4.58477 3.61503
0.02833 3.97375 4.69610 0.61958 0.77255 0.48576 0.95510
29 2.77967 5.15838 2.57372 1.71342 4.42996 3.34947 3.12871 3.91441 2.35752 3.42625
4.24371 2.83040 3.83308 2.13340 2.77192 2.73557 3.01841 3.53966 5.56516 4.18423 33 - -
2.68618 4.42225 2.77519 2.73123 3.46354 2.40513 3.72494 3.29354 2.67741 2.69355
4.24690 2.90346 2.73739 3.18146 2.89801 2.37887 2.77519 2.98518 4.58477 3.61503
0.02833 3.97375 4.69610 0.61958 0.77255 0.48576 0.95510
30 3.09946 4.50131 4.52644 3.98740 3.17083 4.27439 4.63437 1.73441 3.81794 1.62670
1.69279 4.26030 4.55871 4.05406 4.00257 3.62253 3.34262 2.09371 5.13721 3.99386 34 - -
2.68618 4.42225 2.77519 2.73123 3.46354 2.40513 3.72494 3.29354 2.67741 2.69355

4.24690 2.90346 2.73739 3.18146 2.89801 2.37887 2.77519 2.98518 4.58477 3.61503
0.02833 3.97375 4.69610 0.61958 0.77255 0.48576 0.95510
31 3.07986 4.41706 4.72294 4.23413 3.50600 4.38074 4.95376 1.14940 4.10853 2.00069
3.32198 4.46092 4.70856 4.38021 4.30957 3.77227 3.35074 1.42002 5.47839 4.27317 35 - -
2.68618 4.42225 2.77519 2.73123 3.46354 2.40513 3.72494 3.29354 2.67741 2.69355
4.24690 2.90346 2.73739 3.18146 2.89801 2.37887 2.77519 2.98518 4.58477 3.61503
0.02833 3.97375 4.69610 0.61958 0.77255 0.48576 0.95510
32 2.47888 5.21773 1.72975 2.09367 4.52016 3.26670 3.66598 3.99811 2.55271 3.51886
4.32451 2.59909 3.79702 2.54902 3.08315 2.50129 3.01920 3.59577 5.69553 4.26035 36 - -
2.68618 4.42225 2.77519 2.73123 3.46354 2.40513 3.72494 3.29354 2.67741 2.69355
4.24690 2.90346 2.73739 3.18146 2.89801 2.37887 2.77519 2.98518 4.58477 3.61503
0.02833 3.97375 4.69610 0.61958 0.77255 0.48576 0.95510
33 2.16478 2.87622 3.65350 3.13556 3.59063 3.25593 3.99610 2.61705 3.06567 2.65344
3.55297 3.38040 3.82749 3.35456 3.36932 2.03476 2.34134 2.57488 5.04503 3.83275 37 - -
2.68618 4.42225 2.77519 2.73123 3.46354 2.40513 3.72494 3.29354 2.67741 2.69355
4.24690 2.90346 2.73739 3.18146 2.89801 2.37887 2.77519 2.98518 4.58477 3.61503
0.02833 3.97375 4.69610 0.61958 0.77255 0.48576 0.95510
34 3.53046 4.82686 4.45953 4.11921 1.62515 4.28914 3.46671 3.25902 3.97766 2.61588
3.87119 3.98096 4.61472 4.01448 4.06412 3.65599 3.75884 3.17650 3.63139 1.01943 38 - -
2.68618 4.42225 2.77519 2.73123 3.46354 2.40513 3.72494 3.29354 2.67741 2.69355
4.24690 2.90346 2.73739 3.18146 2.89801 2.37887 2.77519 2.98518 4.58477 3.61503
0.02833 3.97375 4.69610 0.61958 0.77255 0.48576 0.95510
35 2.75761 4.29615 3.88503 2.80989 3.40939 3.85290 4.16499 1.82249 3.24093 1.99078
3.31220 3.68544 4.20804 3.54709 3.53441 3.13901 2.99599 1.71933 5.04324 3.83069 39 - -
2.68618 4.42225 2.77519 2.73123 3.46354 2.40513 3.72494 3.29354 2.67741 2.69355
4.24690 2.90346 2.73739 3.18146 2.89801 2.37887 2.77519 2.98518 4.58477 3.61503
0.02833 3.97375 4.69610 0.61958 0.77255 0.48576 0.95510
36 2.77510 5.16108 2.68692 2.07424 4.49802 3.38432 3.60780 3.93802 1.92211 3.42989
4.23470 2.58597 3.83831 2.09956 2.63045 2.73278 3.00257 3.55502 5.56037 4.21032 40 - -
2.68618 4.42225 2.77519 2.73123 3.46354 2.40513 3.72494 3.29354 2.67741 2.69355
4.24690 2.90346 2.73739 3.18146 2.89801 2.37887 2.77519 2.98518 4.58477 3.61503
0.02833 3.97375 4.69610 0.61958 0.77255 0.48576 0.95510
37 2.46938 4.31437 3.52024 3.27794 4.06049 3.15005 4.28724 3.20004 3.22291 3.05992
4.10460 3.47043 3.86209 3.61285 3.47620 2.67187 1.01114 2.88411 5.48938 4.27737 41 - -
2.68618 4.42225 2.77519 2.73123 3.46354 2.40513 3.72494 3.29354 2.67741 2.69355
4.24690 2.90346 2.73739 3.18146 2.89801 2.37887 2.77519 2.98518 4.58477 3.61503
0.02833 3.97375 4.69610 0.61958 0.77255 0.48576 0.95510
38 2.82838 3.04128 4.31314 3.80436 3.24730 3.84575 4.40519 2.24826 3.61177 1.16995
3.20239 3.98865 4.29362 3.89426 3.79622 3.23880 3.12775 2.16842 5.02429 3.79873 42 - -
2.68618 4.42225 2.77519 2.73123 3.46354 2.40513 3.72494 3.29354 2.67741 2.69355
4.24690 2.90346 2.73739 3.18146 2.89801 2.37887 2.77519 2.98518 4.58477 3.61503
0.02833 3.97375 4.69610 0.61958 0.77255 0.48576 0.95510
39 1.05307 4.15624 3.53700 3.28060 4.15251 2.99876 4.29563 3.22931 3.27702 3.13973
4.10116 3.39473 3.74773 3.60144 3.54271 2.47223 2.52233 2.84428 5.59283 4.38371 43 - -

2.68618 4.42225 2.77519 2.73123 3.46354 2.40513 3.72494 3.29354 2.67741 2.69355
4.24690 2.90346 2.73739 3.18146 2.89801 2.37887 2.77519 2.98518 4.58477 3.61503
0.02833 3.97375 4.69610 0.61958 0.77255 0.48576 0.95510
40 2.54708 4.78924 3.01139 2.46278 4.05721 3.42061 3.60865 3.45669 1.97623 3.05435
3.40238 2.96516 3.81792 2.35379 2.70866 2.56125 2.63467 3.13851 5.28532 3.97513 44 - -
2.68618 4.42225 2.77519 2.73123 3.46354 2.40513 3.72494 3.29354 2.67741 2.69355
4.24690 2.90346 2.73739 3.18146 2.89801 2.37887 2.77519 2.98518 4.58477 3.61503
0.02833 3.97375 4.69610 0.61958 0.77255 0.48576 0.95510
41 2.36616 4.20198 3.80868 3.29469 3.50697 2.89706 4.12381 2.10002 3.21741 2.43837
3.44301 3.57684 4.04413 3.51359 3.50409 2.89856 2.87605 1.58899 5.05751 3.84005 45 - -
2.68618 4.42225 2.77519 2.73123 3.46354 2.40513 3.72494 3.29354 2.67741 2.69355
4.24690 2.90346 2.73739 3.18146 2.89801 2.37887 2.77519 2.98518 4.58477 3.61503
0.02833 3.97375 4.69610 0.61958 0.77255 0.48576 0.95510
42 3.11302 4.48419 4.61845 4.08427 3.25857 4.34779 4.74979 1.90327 3.93894 1.22566
3.08655 4.34998 4.62889 4.16968 4.13005 3.70213 3.36150 1.54188 5.24099 4.08054 46 - -
2.68618 4.42225 2.77519 2.73123 3.46354 2.40513 3.72494 3.29354 2.67741 2.69355
4.24690 2.90346 2.73739 3.18146 2.89801 2.37887 2.77519 2.98518 4.58477 3.61503
0.02833 3.97375 4.69610 0.61958 0.77255 0.48576 0.95510
43 2.06378 4.24838 3.24651 3.09691 4.37578 1.10936 4.26886 3.76224 3.26900 3.50000
4.38010 3.29933 3.73068 3.56175 3.56379 2.48971 2.81617 3.23487 5.69100 4.49357 47 - -
2.68619 4.42226 2.77520 2.73124 3.46355 2.40514 3.72495 3.29325 2.67742 2.69356
4.24691 2.90348 2.73740 3.18147 2.89802 2.37888 2.77520 2.98519 4.58478 3.61504
0.10905 2.36252 4.69610 0.40503 1.09949 0.48576 0.95510
44 2.53063 4.40009 3.37180 2.93961 3.44502 3.30610 3.85618 3.24904 2.82726 2.89273
3.82485 3.27298 3.87020 3.21792 3.14776 1.36534 2.87851 2.94845 3.91361 3.49589 49 - -
2.68618 4.42225 2.77519 2.73123 3.46354 2.40513 3.72494 3.29354 2.67741 2.69355
4.24690 2.90346 2.73739 3.18146 2.89801 2.37887 2.77519 2.98518 4.58477 3.61503
0.02833 3.97375 4.69610 0.61958 0.77255 0.48576 0.95510
45 2.58717 4.60987 3.08445 1.97532 3.77471 3.44828 3.66017 3.15304 2.49052 2.64431
3.25990 3.03032 3.83773 2.74814 2.90956 2.67544 2.81784 2.75076 5.12943 3.57429 50 - -
2.68618 4.42225 2.77519 2.73123 3.46354 2.40513 3.72494 3.29354 2.67741 2.69355
4.24690 2.90346 2.73739 3.18146 2.89801 2.37887 2.77519 2.98518 4.58477 3.61503
0.02833 3.97375 4.69610 0.61958 0.77255 0.48576 0.95510
46 2.92647 5.35652 2.09130 1.14056 4.66194 3.24435 3.77839 4.14713 2.74573 3.70004
4.57211 2.74523 3.85382 2.95335 3.28132 2.84028 3.21555 3.75719 5.84678 4.40597 51 - -
2.68618 4.42225 2.77519 2.73123 3.46354 2.40513 3.72494 3.29354 2.67741 2.69355
4.24690 2.90346 2.73739 3.18146 2.89801 2.37887 2.77519 2.98518 4.58477 3.61503
0.02833 3.97375 4.69610 0.61958 0.77255 0.48576 0.95510
47 3.04173 5.17114 2.53371 0.88956 4.53041 3.34102 3.93759 4.00452 2.83102 3.60573
4.57608 2.99373 3.95065 3.15164 3.24977 3.01432 3.34700 3.67821 5.68294 4.40615 52 - -
2.68618 4.42225 2.77519 2.73123 3.46354 2.40513 3.72494 3.29354 2.67741 2.69355
4.24690 2.90346 2.73739 3.18146 2.89801 2.37887 2.77519 2.98518 4.58477 3.61503
0.02833 3.97375 4.69610 0.61958 0.77255 0.48576 0.95510
48 0.87712 4.24472 3.56722 3.36407 4.15493 3.06637 4.37391 3.29575 3.38297 3.17260

4.19398 3.48751 3.81525 3.71502 3.62601 2.59448 2.87889 2.93261 5.57198 4.38372 53 - -
2.68618 4.42225 2.77519 2.73123 3.46354 2.40513 3.72494 3.29354 2.67741 2.69355
4.24690 2.90346 2.73739 3.18146 2.89801 2.37887 2.77519 2.98518 4.58477 3.61503
0.02833 3.97375 4.69610 0.61958 0.77255 0.48576 0.95510
49 3.02629 5.13136 3.39804 2.77289 4.57088 3.63502 3.61469 3.93004 1.20565 3.38619
4.26178 3.18015 4.01777 2.75383 1.82594 3.02595 3.20207 3.61341 5.43671 4.26028 54 - -
2.68618 4.42225 2.77519 2.73123 3.46354 2.40513 3.72494 3.29354 2.67741 2.69355
4.24690 2.90346 2.73739 3.18146 2.89801 2.37887 2.77519 2.98518 4.58477 3.61503
0.02833 3.97375 4.69610 0.61958 0.77255 0.48576 0.95510
50 2.81532 5.00238 3.21147 2.59208 4.34147 3.54177 3.58472 3.72420 1.50725 3.23697
3.58173 3.05594 3.91305 2.40002 2.16171 2.82302 3.01117 3.39957 5.37540 4.12474 55 - -
2.68618 4.42225 2.77519 2.73123 3.46354 2.40513 3.72494 3.29354 2.67741 2.69355
4.24690 2.90346 2.73739 3.18146 2.89801 2.37887 2.77519 2.98518 4.58477 3.61503
0.02833 3.97375 4.69610 0.61958 0.77255 0.48576 0.95510
51 2.79338 5.03633 3.05257 2.52133 4.41732 3.48305 3.59421 3.81810 1.59342 3.31949
4.14168 2.68861 3.88734 2.72597 2.08487 2.54780 3.00546 3.46557 5.43925 4.16550 56 - -
2.68618 4.42225 2.77519 2.73123 3.46354 2.40513 3.72494 3.29354 2.67741 2.69355
4.24690 2.90346 2.73739 3.18146 2.89801 2.37887 2.77519 2.98518 4.58477 3.61503
0.02833 3.97375 4.69610 0.61958 0.77255 0.48576 0.95510
52 3.11005 4.47149 4.63736 4.10294 3.24918 4.34960 4.74209 1.28136 3.94362 1.70872
2.34112 4.35831 4.62501 4.17458 4.12085 3.70557 3.35475 1.91854 5.22222 4.07262 57 - -
2.68618 4.42225 2.77519 2.73123 3.46354 2.40513 3.72494 3.29354 2.67741 2.69355
4.24690 2.90346 2.73739 3.18146 2.89801 2.37887 2.77519 2.98518 4.58477 3.61503
0.02833 3.97375 4.69610 0.61958 0.77255 0.48576 0.95510
53 3.39568 4.84357 3.98872 3.73778 2.30216 3.94887 3.66942 3.39098 3.59241 2.81861
4.05566 3.84973 4.42068 3.87405 3.75508 3.52203 3.68303 3.26642 3.94956 0.81268 58 - -
2.68618 4.42225 2.77519 2.73123 3.46354 2.40513 3.72494 3.29354 2.67741 2.69355
4.24690 2.90346 2.73739 3.18146 2.89801 2.37887 2.77519 2.98518 4.58477 3.61503
0.02833 3.97375 4.69610 0.61958 0.77255 0.48576 0.95510
54 1.79425 4.39567 3.01752 2.70972 4.22462 3.07406 3.93222 3.64462 2.82762 3.29726
4.12411 2.35098 3.72560 3.14269 3.22446 1.81451 2.77765 3.18755 5.52842 4.24304 59 - -
2.68618 4.42225 2.77519 2.73123 3.46354 2.40513 3.72494 3.29354 2.67741 2.69355
4.24690 2.90346 2.73739 3.18146 2.89801 2.37887 2.77519 2.98518 4.58477 3.61503
0.02833 3.97375 4.69610 0.61958 0.77255 0.48576 0.95510
55 2.81611 3.59445 4.42927 3.87040 2.70029 4.01664 4.40679 1.88393 3.75427 2.06049
3.21692 4.05804 4.36455 3.96621 3.92463 3.34201 3.06483 1.35093 4.98590 3.76868 60 - -
2.68618 4.42225 2.77519 2.73123 3.46354 2.40513 3.72494 3.29354 2.67741 2.69355
4.24690 2.90346 2.73739 3.18146 2.89801 2.37887 2.77519 2.98518 4.58477 3.61503
0.02833 3.97375 4.69610 0.61958 0.77255 0.48576 0.95510
56 2.20298 2.67020 3.76562 3.39011 4.00624 2.96706 4.25594 3.34438 3.30020 3.13088
4.01429 3.42587 3.70766 3.60089 3.55229 1.23541 2.68454 2.90975 5.42672 4.21089 61 - -
2.68618 4.42225 2.77519 2.73123 3.46354 2.40513 3.72494 3.29354 2.67741 2.69355
4.24690 2.90346 2.73739 3.18146 2.89801 2.37887 2.77519 2.98518 4.58477 3.61503
0.02833 3.97375 4.69610 0.61958 0.77255 0.48576 0.95510

57 2.29634 2.60149 3.88273 3.46575 3.83718 3.10362 4.26948 2.91266 3.33552 2.84526
3.80688 3.51999 3.80266 3.64427 3.57167 2.54116 1.41663 2.60208 5.32086 4.12269 62 - -
2.68618 4.42225 2.77519 2.73123 3.46354 2.40513 3.72494 3.29354 2.67741 2.69355
4.24690 2.90346 2.73739 3.18146 2.89801 2.37887 2.77519 2.98518 4.58477 3.61503
0.02833 3.97375 4.69610 0.61958 0.77255 0.48576 0.95510
58 2.64861 4.90171 2.89007 2.12515 4.20170 3.39545 3.60152 3.61926 2.16917 3.18352
3.99407 2.91794 3.81140 2.74128 2.43705 2.48276 2.31591 3.27420 5.38052 4.05120 63 - -
2.68618 4.42225 2.77519 2.73123 3.46354 2.40513 3.72494 3.29354 2.67741 2.69355
4.24690 2.90346 2.73739 3.18146 2.89801 2.37887 2.77519 2.98518 4.58477 3.61503
0.02833 3.97375 4.69610 0.61958 0.77255 0.48576 0.95510
59 2.56121 3.95369 3.18953 2.62713 3.66829 3.45332 3.35754 3.05813 2.54971 2.72971
3.59377 3.09123 3.84645 2.91675 2.54826 2.55452 2.29441 2.79649 5.03910 3.34805 64 - -
2.68618 4.42225 2.77519 2.73123 3.46354 2.40513 3.72494 3.29354 2.67741 2.69355
4.24690 2.90346 2.73739 3.18146 2.89801 2.37887 2.77519 2.98518 4.58477 3.61503
0.02833 3.97375 4.69610 0.61958 0.77255 0.48576 0.95510
60 3.39568 4.84357 3.98872 3.73778 2.30216 3.94887 3.66942 3.39098 3.59241 2.81861
4.05566 3.84973 4.42068 3.87405 3.75508 3.52203 3.68303 3.26642 3.94956 0.81268 65 - -
2.68618 4.42225 2.77519 2.73123 3.46354 2.40513 3.72494 3.29354 2.67741 2.69355
4.24690 2.90346 2.73739 3.18146 2.89801 2.37887 2.77519 2.98518 4.58477 3.61503
0.02833 3.97375 4.69610 0.61958 0.77255 0.48576 0.95510
61 2.58508 4.25739 3.52491 2.96336 2.60730 3.57323 3.79521 2.71582 2.89305 2.35455
3.34099 3.33946 3.95152 2.79840 3.21897 2.83037 2.43989 2.42826 4.73037 2.91104 66 - -
2.68618 4.42225 2.77519 2.73123 3.46354 2.40513 3.72494 3.29354 2.67741 2.69355
4.24690 2.90346 2.73739 3.18146 2.89801 2.37887 2.77519 2.98518 4.58477 3.61503
0.02833 3.97375 4.69610 0.61958 0.77255 0.48576 0.95510
62 1.84551 4.20814 3.28718 3.10636 4.35637 1.24974 4.25466 3.72364 3.25538 3.46442
4.32810 3.29078 3.70829 3.54080 3.55572 2.44745 2.76985 3.19196 5.68495 4.48373 67 - -
2.68618 4.42225 2.77519 2.73123 3.46354 2.40513 3.72494 3.29354 2.67741 2.69355
4.24690 2.90346 2.73739 3.18146 2.89801 2.37887 2.77519 2.98518 4.58477 3.61503
0.02833 3.97375 4.69610 0.61958 0.77255 0.48576 0.95510
63 3.38912 4.76145 4.37752 4.11195 0.85951 4.05377 3.92510 2.90111 4.01660 2.24819
3.61677 4.13423 4.51063 4.15687 4.11129 3.65338 3.69058 2.89912 4.14548 2.50035 68 - -
2.68618 4.42225 2.77519 2.73123 3.46354 2.40513 3.72494 3.29354 2.67741 2.69355
4.24690 2.90346 2.73739 3.18146 2.89801 2.37887 2.77519 2.98518 4.58477 3.61503
0.02833 3.97375 4.69610 0.61958 0.77255 0.48576 0.95510
64 2.66221 4.76039 2.75906 2.52652 4.29462 1.71736 3.82587 3.78703 2.60855 3.37192
4.23809 2.99839 3.83391 2.05807 2.98013 2.71549 2.99850 3.39607 5.52511 4.21250 69 - -
2.68618 4.42225 2.77519 2.73123 3.46354 2.40513 3.72494 3.29354 2.67741 2.69355
4.24690 2.90346 2.73739 3.18146 2.89801 2.37887 2.77519 2.98518 4.58477 3.61503
0.02833 3.97375 4.69610 0.61958 0.77255 0.48576 0.95510
65 1.43853 1.96426 3.99097 3.61709 3.89974 3.03758 4.36856 2.96324 3.49726 2.91529
3.87275 3.56405 3.77399 3.77152 3.69856 2.49312 2.71951 2.62799 5.38694 4.20539 70 - -
2.68618 4.42225 2.77519 2.73123 3.46354 2.40513 3.72494 3.29354 2.67741 2.69355
4.24690 2.90346 2.73739 3.18146 2.89801 2.37887 2.77519 2.98518 4.58477 3.61503

0.02833 3.97375 4.69610 0.61958 0.77255 0.48576 0.95510
66 2.58104 4.49112 3.20248 2.33353 3.66774 3.47897 3.70493 2.82553 2.56607 2.32396
3.57858 3.11036 3.86658 2.93527 2.74755 2.71445 2.48686 2.64024 5.05255 3.78415 71 - -
2.68618 4.42225 2.77519 2.73123 3.46354 2.40513 3.72494 3.29354 2.67741 2.69355
4.24690 2.90346 2.73739 3.18146 2.89801 2.37887 2.77519 2.98518 4.58477 3.61503
0.02833 3.97375 4.69610 0.61958 0.77255 0.48576 0.95510
67 3.05767 4.39853 4.67547 4.12762 3.32725 4.34993 4.76284 1.40657 4.01172 1.81882
2.46296 4.36724 4.62308 4.21793 4.18979 3.69342 3.29993 1.55228 5.25909 4.11562 72 - -
2.68618 4.42225 2.77519 2.73123 3.46354 2.40513 3.72494 3.29354 2.67741 2.69355
4.24690 2.90346 2.73739 3.18146 2.89801 2.37887 2.77519 2.98518 4.58477 3.61503
0.02833 3.97375 4.69610 0.61958 0.77255 0.48576 0.95510
68 2.62686 4.91128 2.17906 2.31268 4.23830 3.30181 3.19189 3.68897 2.48213 3.26193
4.07059 2.84738 3.78248 2.79752 2.96501 2.21233 2.38419 3.32115 5.46774 4.09378 73 - -
2.68618 4.42225 2.77519 2.73123 3.46354 2.40513 3.72494 3.29354 2.67741 2.69355
4.24690 2.90346 2.73739 3.18146 2.89801 2.37887 2.77519 2.98518 4.58477 3.61503
0.02833 3.97375 4.69610 0.61958 0.77255 0.48576 0.95510
69 2.76560 4.89003 2.73413 1.41145 4.08539 3.39343 3.76439 2.94738 2.56421 3.04442
4.01512 2.97530 3.89560 2.94852 2.97275 2.80106 3.03117 3.06162 5.43079 4.07762 74 - -
2.68618 4.42225 2.77519 2.73123 3.46354 2.40513 3.72494 3.29354 2.67741 2.69355
4.24690 2.90346 2.73739 3.18146 2.89801 2.37887 2.77519 2.98518 4.58477 3.61503
0.02833 3.97375 4.69610 0.61958 0.77255 0.48576 0.95510
70 2.75381 5.20867 2.27696 1.55575 4.51109 2.95309 3.65985 3.98701 2.52986 3.50640
4.31071 2.75037 3.21692 2.61512 3.04958 2.69427 3.01208 3.58619 5.68057 4.25118 75 - -
2.68618 4.42225 2.77519 2.73123 3.46354 2.40513 3.72494 3.29354 2.67741 2.69355
4.24690 2.90346 2.73739 3.18146 2.89801 2.37887 2.77519 2.98518 4.58477 3.61503
0.02833 3.97375 4.69610 0.61958 0.77255 0.48576 0.95510
71 2.61425 4.42197 3.32420 2.49863 3.56479 3.53473 3.80173 2.77472 2.70419 2.05403
3.27125 3.22800 3.93181 3.06933 3.06686 2.79767 2.28295 2.56989 5.02300 3.77350 76 - -
2.68618 4.42225 2.77519 2.73123 3.46354 2.40513 3.72494 3.29354 2.67741 2.69355
4.24690 2.90346 2.73739 3.18146 2.89801 2.37887 2.77519 2.98518 4.58477 3.61503
0.02833 3.97375 4.69610 0.61958 0.77255 0.48576 0.95510
72 2.27994 4.19638 3.44937 3.07484 4.05781 3.03893 4.10339 3.31761 3.04284 3.11387
4.00149 3.28949 3.73193 3.37515 3.36229 1.34132 2.39740 2.59411 5.45087 4.21272 77 - -
2.68618 4.42225 2.77519 2.73123 3.46354 2.40513 3.72494 3.29354 2.67741 2.69355
4.24690 2.90346 2.73739 3.18146 2.89801 2.37887 2.77519 2.98518 4.58477 3.61503
0.02833 3.97375 4.69610 0.61958 0.77255 0.48576 0.95510
73 2.57983 4.16145 2.97684 2.09809 3.89450 3.40650 3.37002 3.32466 2.43989 2.94945
3.78228 2.62330 3.80590 2.79844 2.88080 2.63510 2.74705 3.02320 5.19898 3.05768 78 - -
2.68618 4.42225 2.77519 2.73123 3.46354 2.40513 3.72494 3.29354 2.67741 2.69355
4.24690 2.90346 2.73739 3.18146 2.89801 2.37887 2.77519 2.98518 4.58477 3.61503
0.02833 3.97375 4.69610 0.61958 0.77255 0.48576 0.95510
74 3.06627 5.03236 3.24889 2.85242 4.44195 3.54396 3.81802 3.87776 0.90773 3.42116
4.37793 3.27214 4.03779 3.00431 2.45464 3.10207 3.31240 3.58010 5.45561 4.28327 79 - -
2.68618 4.42225 2.77519 2.73123 3.46354 2.40513 3.72494 3.29354 2.67741 2.69355

4.24690 2.90346 2.73739 3.18146 2.89801 2.37887 2.77519 2.98518 4.58477 3.61503
0.02833 3.97375 4.69610 0.61958 0.77255 0.48576 0.95510
75 3.10925 4.46565 4.66733 4.10467 2.16401 4.32446 4.56026 1.97374 3.98591 1.25764
2.93704 4.32226 4.56661 4.11220 4.12010 3.65238 3.33524 1.99883 4.94120 3.72544 80 - -
2.68618 4.42225 2.77519 2.73123 3.46354 2.40513 3.72494 3.29354 2.67741 2.69355
4.24690 2.90346 2.73739 3.18146 2.89801 2.37887 2.77519 2.98518 4.58477 3.61503
0.02833 3.97375 4.69610 0.61958 0.77255 0.48576 0.95510
76 2.87339 5.13835 3.06309 2.14534 4.53163 3.52307 3.58659 3.91749 1.56773 3.38610
4.21420 3.01648 3.91902 2.71386 1.95001 2.85050 3.06878 3.56534 5.47550 4.21991 81 - -
2.68618 4.42225 2.77519 2.73123 3.46354 2.40513 3.72494 3.29354 2.67741 2.69355
4.24690 2.90346 2.73739 3.18146 2.89801 2.37887 2.77519 2.98518 4.58477 3.61503
0.02833 3.97375 4.69610 0.61958 0.77255 0.48576 0.95510
77 2.57202 5.08438 2.14491 1.99414 4.45274 2.15038 3.69420 3.91578 2.59542 3.46462
4.27889 2.77343 3.79310 2.84711 3.11882 2.44275 2.99539 3.51828 5.66039 4.24630 82 - -
2.68618 4.42225 2.77519 2.73123 3.46354 2.40513 3.72494 3.29354 2.67741 2.69355
4.24690 2.90346 2.73739 3.18146 2.89801 2.37887 2.77519 2.98518 4.58477 3.61503
0.02833 3.97375 4.69610 0.61958 0.77255 0.48576 0.95510
78 3.24928 4.63318 4.62121 4.11796 3.10057 4.40263 4.72987 1.97277 3.90241 0.90973
2.96896 4.39569 4.66725 4.15329 4.07124 3.78890 3.49593 2.17665 5.14001 3.94822 83 - -
2.68618 4.42225 2.77519 2.73123 3.46354 2.40513 3.72494 3.29354 2.67741 2.69355
4.24690 2.90346 2.73739 3.18146 2.89801 2.37887 2.77519 2.98518 4.58477 3.61503
0.02833 3.97375 4.69610 0.61958 0.77255 0.48576 0.95510
79 2.40092 4.34749 3.14476 2.94543 4.20091 3.05282 4.11394 3.67596 3.02867 3.36392
4.26340 3.23189 1.28272 3.38872 3.34046 2.31699 2.85833 3.21709 5.54251 4.27019 84 - -
2.68618 4.42225 2.77519 2.73123 3.46354 2.40513 3.72494 3.29354 2.67741 2.69355
4.24690 2.90346 2.73739 3.18146 2.89801 2.37887 2.77519 2.98518 4.58477 3.61503
0.02833 3.97375 4.69610 0.61958 0.77255 0.48576 0.95510
80 2.73060 5.04106 2.22100 2.25040 4.43844 1.80535 3.72502 3.90023 2.41603 3.46172
4.29145 2.60075 3.80727 2.88979 3.11105 2.70720 3.02156 3.50889 5.65712 4.25895 85 - -
2.68618 4.42225 2.77519 2.73123 3.46354 2.40513 3.72494 3.29354 2.67741 2.69355
4.24690 2.90346 2.73739 3.18146 2.89801 2.37887 2.77519 2.98518 4.58477 3.61503
0.02833 3.97375 4.69610 0.61958 0.77255 0.48576 0.95510
81 2.88836 4.40378 4.26472 3.93173 3.62546 3.82775 4.71637 2.04952 3.80104 2.29541
3.57597 4.10508 4.38316 4.15388 4.00366 3.33823 3.24779 0.90630 5.42675 4.16432 86 - -
2.68618 4.42225 2.77519 2.73123 3.46354 2.40513 3.72494 3.29354 2.67741 2.69355
4.24690 2.90346 2.73739 3.18146 2.89801 2.37887 2.77519 2.98518 4.58477 3.61503
0.02833 3.97375 4.69610 0.61958 0.77255 0.48576 0.95510
82 2.65087 4.52954 3.30908 2.72399 3.70104 3.52816 3.69779 3.05341 2.27836 2.07990
3.60956 3.16332 3.90747 2.73827 2.46403 2.78734 2.87254 2.56286 5.05345 3.80719 87 - -
2.68618 4.42225 2.77519 2.73123 3.46354 2.40513 3.72494 3.29354 2.67741 2.69355
4.24690 2.90346 2.73739 3.18146 2.89801 2.37887 2.77519 2.98518 4.58477 3.61503
0.02833 3.97375 4.69610 0.61958 0.77255 0.48576 0.95510
83 3.50585 4.75593 4.61507 4.25774 1.39911 4.31755 3.44653 3.24753 4.08692 2.61876
3.83019 4.02117 4.62047 4.06335 4.11782 3.66479 3.72239 3.14823 1.80989 1.72923 88 - -

2.68618 4.42225 2.77519 2.73123 3.46354 2.40513 3.72494 3.29354 2.67741 2.69355
4.24690 2.90346 2.73739 3.18146 2.89801 2.37887 2.77519 2.98518 4.58477 3.61503
0.02833 3.97375 4.69610 0.61958 0.77255 0.48576 0.95510
84 3.05113 4.39625 4.68617 4.19510 3.54449 4.34648 4.92915 1.48313 4.07596 2.06624
3.36458 4.42339 4.68640 4.35629 4.28695 3.73427 3.32408 1.09026 5.48932 4.27375 89 - -
2.68618 4.42225 2.77519 2.73123 3.46354 2.40513 3.72494 3.29354 2.67741 2.69355
4.24690 2.90346 2.73739 3.18146 2.89801 2.37887 2.77519 2.98518 4.58477 3.61503
0.02833 3.97375 4.69610 0.61958 0.77255 0.48576 0.95510
85 3.24924 4.63332 4.62072 4.11761 3.10045 4.40224 4.72951 1.97367 3.90190 0.90946
2.96904 4.39533 4.66705 4.15299 4.07075 3.78861 3.49595 2.17707 5.13978 3.94777 90 - -
2.68618 4.42225 2.77519 2.73123 3.46354 2.40513 3.72494 3.29354 2.67741 2.69355
4.24690 2.90346 2.73739 3.18146 2.89801 2.37887 2.77519 2.98518 4.58477 3.61503
0.02833 3.97375 4.69610 0.61958 0.77255 0.48576 0.95510
86 2.89008 4.62983 3.50832 3.36250 4.34781 3.31164 4.43108 3.91993 3.42859 3.53554
4.59019 3.65867 0.65914 3.81430 3.66208 3.06090 3.33977 3.55365 5.50596 4.47009 91 - -
2.68618 4.42225 2.77519 2.73123 3.46354 2.40513 3.72494 3.29354 2.67741 2.69355
4.24690 2.90346 2.73739 3.18146 2.89801 2.37887 2.77519 2.98518 4.58477 3.61503
0.02833 3.97375 4.69610 0.61958 0.77255 0.48576 0.95510
87 3.10050 5.24170 0.78299 2.40253 4.63282 3.28927 4.02330 4.24449 3.12706 3.83512
4.81618 2.96369 3.94931 3.26316 3.65644 3.05197 3.44151 3.87315 5.78156 4.48997 92 - -
2.68618 4.42225 2.77519 2.73123 3.46354 2.40513 3.72494 3.29354 2.67741 2.69355
4.24690 2.90346 2.73739 3.18146 2.89801 2.37887 2.77519 2.98518 4.58477 3.61503
0.02833 3.97375 4.69610 0.61958 0.77255 0.48576 0.95510
88 2.36689 4.28823 3.29660 3.11780 4.17815 3.01633 4.22450 3.71480 3.19840 3.42748
4.34560 3.32586 3.76365 3.53994 3.48081 0.94800 2.85474 3.22768 5.54269 4.26240 93 - -
2.68618 4.42225 2.77519 2.73123 3.46354 2.40513 3.72494 3.29354 2.67741 2.69355
4.24690 2.90346 2.73739 3.18146 2.89801 2.37887 2.77519 2.98518 4.58477 3.61503
0.02833 3.97375 4.69610 0.61958 0.77255 0.48576 0.95510
89 3.49391 4.82482 4.37713 4.03259 1.78619 4.24578 3.48168 3.24921 3.89339 2.61995
3.87303 3.94684 4.58564 3.97079 4.00566 3.61866 3.72829 3.16245 3.66183 0.96654 94 - -
2.68618 4.42225 2.77519 2.73123 3.46354 2.40513 3.72494 3.29354 2.67741 2.69355
4.24690 2.90346 2.73739 3.18146 2.89801 2.37887 2.77519 2.98518 4.58477 3.61503
0.02833 3.97375 4.69610 0.61958 0.77255 0.48576 0.95510
90 3.12834 4.44628 4.77057 4.23252 3.34047 4.45695 4.88190 1.44607 4.11478 1.46715
3.14347 4.47823 4.70886 4.31942 4.29117 3.81328 3.37148 1.52537 5.33272 4.18606 95 - -
2.68618 4.42225 2.77519 2.73123 3.46354 2.40513 3.72494 3.29354 2.67741 2.69355
4.24690 2.90346 2.73739 3.18146 2.89801 2.37887 2.77519 2.98518 4.58477 3.61503
0.02833 3.97375 4.69610 0.61958 0.77255 0.48576 0.95510
91 2.69454 4.87494 1.63874 2.37565 3.95889 3.34547 3.70398 3.44205 2.60173 3.11205
4.01167 2.90434 3.84191 2.89865 3.07539 2.71589 2.95969 2.81022 5.32693 3.52798 96 - -
2.68618 4.42225 2.77519 2.73123 3.46354 2.40513 3.72494 3.29354 2.67741 2.69355
4.24690 2.90346 2.73739 3.18146 2.89801 2.37887 2.77519 2.98518 4.58477 3.61503
0.02833 3.97375 4.69610 0.61958 0.77255 0.48576 0.95510
92 2.43710 4.53460 2.75908 2.57143 3.81283 3.36930 3.73089 3.14396 2.61941 2.85057

3.71943 3.04432 2.89783 2.84945 3.04170 2.66315 2.82149 2.12189 5.17630 3.88782 97 - -
2.68618 4.42225 2.77519 2.73123 3.46354 2.40513 3.72494 3.29354 2.67741 2.69355
4.24690 2.90346 2.73739 3.18146 2.89801 2.37887 2.77519 2.98518 4.58477 3.61503
0.02833 3.97375 4.69610 0.61958 0.77255 0.48576 0.95510
93 2.66958 5.04935 2.75975 1.92356 4.36588 3.03416 3.58415 3.81121 2.05670 3.32872
4.11654 2.85467 3.32317 2.57245 2.58489 2.64430 2.90029 3.42855 5.48757 4.12301 98 - -
2.68618 4.42225 2.77519 2.73123 3.46354 2.40513 3.72494 3.29354 2.67741 2.69355
4.24690 2.90346 2.73739 3.18146 2.89801 2.37887 2.77519 2.98518 4.58477 3.61503
0.02833 3.97375 4.69610 0.61958 0.77255 0.48576 0.95510
94 2.61648 4.80714 2.60370 2.38458 3.72367 3.36497 3.63334 3.47122 2.47455 3.08160
3.91305 1.98450 3.80348 2.80622 2.93816 2.64405 2.74198 3.14973 5.27105 3.51646 99 - -
2.68618 4.42225 2.77519 2.73123 3.46354 2.40513 3.72494 3.29354 2.67741 2.69355
4.24690 2.90346 2.73739 3.18146 2.89801 2.37887 2.77519 2.98518 4.58477 3.61503
0.02833 3.97375 4.69610 0.61958 0.77255 0.48576 0.95510
95 2.89185 5.05767 2.94012 2.57017 4.45490 3.45866 3.67197 3.89302 1.24530 3.41330
4.27918 2.65963 3.93463 2.82774 2.41178 2.89125 3.12956 3.54664 5.48943 4.21876 100 - -
2.68618 4.42225 2.77519 2.73123 3.46354 2.40513 3.72494 3.29354 2.67741 2.69355
4.24690 2.90346 2.73739 3.18146 2.89801 2.37887 2.77519 2.98518 4.58477 3.61503
0.02833 3.97375 4.69610 0.61958 0.77255 0.48576 0.95510
96 2.69336 5.07092 1.79496 2.08395 4.36125 3.31314 3.63378 3.80485 2.45205 3.35822
4.16430 2.80444 3.79385 2.77292 2.68086 2.66198 2.94180 3.10120 5.54930 4.15397 101 - -
2.68618 4.42225 2.77519 2.73123 3.46354 2.40513 3.72494 3.29354 2.67741 2.69355
4.24690 2.90346 2.73739 3.18146 2.89801 2.37887 2.77519 2.98518 4.58477 3.61503
0.02833 3.97375 4.69610 0.61958 0.77255 0.48576 0.95510
97 3.39568 4.84357 3.98872 3.73778 2.30216 3.94887 3.66942 3.39098 3.59241 2.81861
4.05566 3.84973 4.42068 3.87405 3.75508 3.52203 3.68303 3.26642 3.94956 0.81268 102 - -
2.68618 4.42225 2.77519 2.73123 3.46354 2.40513 3.72494 3.29354 2.67741 2.69355
4.24690 2.90346 2.73739 3.18146 2.89801 2.37887 2.77519 2.98518 4.58477 3.61503
0.02833 3.97375 4.69610 0.61958 0.77255 0.48576 0.95510
98 2.79479 4.55947 3.48924 3.40188 4.54097 0.58863 4.51533 4.16583 3.59688 3.80708
4.78415 3.65362 3.95383 3.91308 3.82119 2.96972 3.28392 3.67461 5.60873 4.64652 103 - -
2.68618 4.42225 2.77519 2.73123 3.46354 2.40513 3.72494 3.29354 2.67741 2.69355
4.24690 2.90346 2.73739 3.18146 2.89801 2.37887 2.77519 2.98518 4.58477 3.61503
0.02833 3.97375 4.69610 0.61958 0.77255 0.48576 0.95510
99 1.75090 4.19705 3.30519 3.11154 4.34643 1.31881 4.24966 3.71044 3.25045 3.45080
4.30997 3.29043 3.70268 3.53418 3.55251 2.43677 2.75720 3.17887 5.67840 4.47673 104 - -
2.68618 4.42225 2.77519 2.73123 3.46354 2.40513 3.72494 3.29354 2.67741 2.69355
4.24690 2.90346 2.73739 3.18146 2.89801 2.37887 2.77519 2.98518 4.58477 3.61503
0.02833 3.97375 4.69610 0.61958 0.77255 0.48576 0.95510
100 2.94160 5.50168 1.52468 1.44879 4.78774 3.21575 3.76036 4.30289 2.77640 3.80996
4.66629 2.68165 3.83733 2.92783 3.36891 2.82573 3.23034 3.88001 5.96170 4.46237 105 - -
2.68618 4.42225 2.77519 2.73123 3.46354 2.40513 3.72494 3.29354 2.67741 2.69355
4.24690 2.90346 2.73739 3.18146 2.89801 2.37887 2.77519 2.98518 4.58477 3.61503
0.02833 3.97375 4.69610 0.61958 0.77255 0.48576 0.95510

101 2.66412 4.65992 3.14209 2.62696 3.87924 3.46541 3.69264 3.23036 1.96386 2.25421
3.77406 3.08959 2.83336 2.88917 2.72538 2.75474 2.90366 2.96852 5.19366 3.91616 106 - -
2.68618 4.42225 2.77519 2.73123 3.46354 2.40513 3.72494 3.29354 2.67741 2.69355
4.24690 2.90346 2.73739 3.18146 2.89801 2.37887 2.77519 2.98518 4.58477 3.61503
0.02833 3.97375 4.69610 0.61958 0.77255 0.48576 0.95510
102 3.46142 4.75394 4.51897 4.14470 1.29494 4.28942 3.49878 3.13328 3.99361 2.38048
3.74548 4.00089 4.59863 4.01876 4.06801 3.63616 3.68337 3.06056 3.66598 1.38683 107 - -
2.68618 4.42225 2.77519 2.73123 3.46354 2.40513 3.72494 3.29354 2.67741 2.69355
4.24690 2.90346 2.73739 3.18146 2.89801 2.37887 2.77519 2.98518 4.58477 3.61503
0.02833 3.97375 4.69610 0.61958 0.77255 0.48576 0.95510
103 2.63345 4.37898 3.15761 2.54006 3.56495 3.58198 3.87570 2.26374 2.84120 2.53513
3.49240 3.29035 3.98081 3.16553 3.21243 2.85668 2.87499 1.80711 5.04367 3.79099 108 - -
2.68618 4.42225 2.77519 2.73123 3.46354 2.40513 3.72494 3.29354 2.67741 2.69355
4.24690 2.90346 2.73739 3.18146 2.89801 2.37887 2.77519 2.98518 4.58477 3.61503
0.02833 3.97375 4.69610 0.61958 0.77255 0.48576 0.95510
104 2.87993 5.38492 1.84759 2.02119 4.68270 3.22380 3.73622 4.19272 2.71146 3.70908
4.55008 1.59430 3.82552 2.90003 3.27531 2.78609 3.16451 3.77782 5.87183 4.39198 109 - -
2.68618 4.42225 2.77519 2.73123 3.46354 2.40513 3.72494 3.29354 2.67741 2.69355
4.24690 2.90346 2.73739 3.18146 2.89801 2.37887 2.77519 2.98518 4.58477 3.61503
0.02833 3.97375 4.69610 0.61958 0.77255 0.48576 0.95510
105 2.79479 4.55947 3.48924 3.40188 4.54097 0.58863 4.51533 4.16583 3.59688 3.80708
4.78415 3.65362 3.95383 3.91308 3.82119 2.96972 3.28392 3.67461 5.60873 4.64652 110 - -
2.68618 4.42225 2.77519 2.73123 3.46354 2.40513 3.72494 3.29354 2.67741 2.69355
4.24690 2.90346 2.73739 3.18146 2.89801 2.37887 2.77519 2.98518 4.58477 3.61503
0.02833 3.97375 4.69610 0.61958 0.77255 0.48576 0.95510
106 2.67543 4.91621 2.82581 1.88484 4.18980 3.39543 3.61698 3.59137 2.11253 3.17379
3.99856 2.91089 3.82291 2.59471 2.73301 2.68329 2.90677 2.69411 5.38781 4.05601 111 - -
2.68618 4.42225 2.77519 2.73123 3.46354 2.40513 3.72494 3.29354 2.67741 2.69355
4.24690 2.90346 2.73739 3.18146 2.89801 2.37887 2.77519 2.98518 4.58477 3.61503
0.02833 3.97375 4.69610 0.61958 0.77255 0.48576 0.95510
107 2.46397 4.31281 4.41932 3.90849 3.54917 4.09178 4.65935 1.35736 3.80725 2.18166
3.39061 4.14783 4.48179 4.08738 4.04110 3.45198 3.17112 1.45782 5.35309 4.14882 112 - -
2.68618 4.42225 2.77519 2.73123 3.46354 2.40513 3.72494 3.29354 2.67741 2.69355
4.24690 2.90346 2.73739 3.18146 2.89801 2.37887 2.77519 2.98518 4.58477 3.61503
0.02833 3.97375 4.69610 0.61958 0.77255 0.48576 0.95510
108 2.83429 4.29243 4.27561 3.75330 3.53692 3.99117 4.52363 1.66616 3.64947 2.22790
3.39772 4.01340 4.39217 3.94084 3.89972 3.33765 2.47342 1.33356 5.28095 4.07174 113 - -
2.68618 4.42225 2.77519 2.73123 3.46354 2.40513 3.72494 3.29354 2.67741 2.69355
4.24690 2.90346 2.73739 3.18146 2.89801 2.37887 2.77519 2.98518 4.58477 3.61503
0.02833 3.97375 4.69610 0.61958 0.77255 0.48576 0.95510
109 2.70544 4.87248 2.81174 2.44544 4.09218 3.37751 3.09504 3.61660 2.38361 3.18855
4.03682 2.94887 2.10138 2.42315 2.75867 2.72839 2.95757 3.28662 5.34254 3.99027 114 - -
2.68618 4.42225 2.77519 2.73123 3.46354 2.40513 3.72494 3.29354 2.67741 2.69355
4.24690 2.90346 2.73739 3.18146 2.89801 2.37887 2.77519 2.98518 4.58477 3.61503

0.01916 3.96458 * 0.61958 0.77255 0.00000 *

//

**Supplemental Data File S2. The DAL domain alignment of 17 plant DAL proteins.sto**

```
# STOCKHOLM 1.0

AT1G72530   VPSLVEGCDYKHWLVLMKPPNGYP.....TRNHIVQSFVETLAMALG.SEEEAKRSIYSV
AT4G20020   DTVLFEGCDYNHWLITMDFSKEETPK...SPEEMVAAYEETCAQGLGISVEEAKQRMYAC
AT5G44780   .....EGCDFNHWLITMNFPKDNLP....SREEMISIFEQTCAKGLAISLEEAKKKIYAI
AT1G32580   MAPLFPGCDYEHWLIVMDKPGGENA....TKQQMIDCYVQTLAKIIG.SEEEAKKKIYNV
AT2G35240   MAPLFPGCDYEHWLIVMEKPGGENA....QKQQMIDCYVQTLAKIVG.SEEEARKKIYNV
AT2G33430   MAPLFPGCDYEHWLIVMDKPGGEGA....TKQQMIDCYIQTLAKVVG.SEEEAKKRIYNV
ZmDAL1      MAPLFPGCDYEHWLIVMDKPGGEGA....TKQQMIDCYIQTLAQVVG.SEEEAKKRIYNV
ZmDAL3      MAPLFPGCDYEHWLIVMDKPGGEGA....TKQQMIDCYIQTLAKVLG.SEEEAKKKIYNV
ZmDAL4      MAPLFPGCDYEHWLIVMDKPGGEGA....SKQQMIDCYIQTLAKVLG.SEEEAKKKIYNV
ZmDAL7      ETILLDGCDYEHWLIVMEFPTDPKP....SEEEMVAAYVKTLAAVLG.SEEEAKKKIYSV
AT3G06790   ETILLDGCDYEHWLIVMEFT.DPKP....TEEEMINSYVKTLTSVLG.WQEEAKKKIYSV
ZmDAL5      ETILLDGCDFEHWLVIMEPPPGDASNPDITRDEIIDSYIKTLAQVVG.SEEEARQKIYSV
AT3G15000   ETILLDGCDFEHWLVVVEPPQGEP.....TRDEIIDSYIKTLAQIVG.SEDEARMKIYSV
ZmDAL2      DEILFEGCDYNHWLITMDFP.DPKP....SREEMIETYLQTLAKVVG.SYEEAKKRMYAF
ZmDAL6      ETILLPGCDYNHWLIVMEFPKDPAP....TREQMIDTYLNTLATVLG.SMEEAKKNMYAF
DAG     ETIMLPGCDYNHWLIVMEFPKDPAP....TREQMIDTYLNTLATVLG.SMEEAKKNMYAF
AT1G11430   ETIMLPGCDYNHWLIVMEFPKDPAP....SRDQMIDTYLNTLATVLG.SMEEAKKNMYAF


AT1G72530   STKYYYAFGCRIHEPLTYKIRSLPDVKWVLPDSFIVDGDNRYGGEPFVDGEVVP
AT4G20020   STTTYQGFQAIMTEQESEKFKDLPGVVFILPDSYIDPQNKEYGGDKYENGVITH
AT5G44780   CTTSYQGFQATMTIGEVEKFRDLPGVQYIIPDSYIDVENKVYGGDKYENGVITP
AT1G32580   SCERYFGFGCEIDEETSNKLEGLPGVLFILPDSYVDQENKDYGAELFVNGEIVQ
AT2G35240   SCERYFGFGCEIDEETSNKLEGLPGVLFVLPDSYVDPEFKDYGAELFVNGEVVP
AT2G33430   SCERYLGFGCEIDEETSTKLEGLPGVLFVLPDSYVDPENKDYGAELFVNGEIVQ
ZmDAL1      SCERYFGFGCEIDEETSNKLEGLPGVLFVLPDSYVDAENKDYGAELFVNGEIVQ
ZmDAL3      SCERYFGFGCEIDEETSNKLEGLPGVLFVLPDSYVDPEYKDYGAELLVNGEIVQ
ZmDAL4      SCERYFGFGCEIDEETSNKLEGLPGVLFVLPDSYVDAEYKDYGAELFVNGEIVQ
ZmDAL7      CTSTYTGFGALISEELSYKVKGLPGVLWVLPDSYLDVPNKDYGGDLFVDGKVIH
AT3G06790   CTSTYTGFGALISEELSCKVKALPGVLWVLPDSYLDVPNKDYGGDLYVEGKVIP
ZmDAL5      STRHYFGFGALVSEELSYKLKEIPKVRWVLPDSYLDVKNKDYGGEPFINGQAVP
AT3G15000   STRCYYAFGALVSEDLSHKLKELSNVRWVLPDSYLDVRNKDYGGEPFIDGKAVP
ZmDAL2      STTTYVGFQAVMTEEMSEKFRGLPGVVFILPDSYLYPETKEYGGDKYDNGVITP
ZmDAL6      STTTYTGFQCTVDEETSEKFKGLPGVLWVLPDSYIDVKNKDYGGDKYVNGEIIP
DAG     STTTYTGFQCTVTEETSEKFKGLPGVLWVLPDSYIDVKNKDYGGDKYVNGEIIP
AT1G11430   STTTYTGFQCTIDEETSEKFKGLPGVLWVLPDSYIDVKNKDYGGDKYINGEIIP
//
```

**Supplemental Data File S3. Alignment of 79 plant DAL proteins for NJ tree construction.fas**

>MA_140268p0010
PWRHLLVRCDCKHWLITLDFPKDPRPTREEMIDTYVKTLAAVLGSEEEAKKKIYALSTTVYTGFQCNIDEATSERLKE
QPLVNWVLPDGYGDPELGIFAGDRYNNGVITPDPNPRPPRKETICREIIDPGRWKEGVIGQWKDETLR
>Al_494504
SFMPDNEGCDFNHWLITMNFPKDNVPSREEMISIFEQTCAKGLDSLEEAKKKIYAICTTSYQGFQATMTIGEVEKFRD
LPGVQYIIPDSYADVENKVYGGDKYENGVIIPVPTKKESKPEQEEAQIIQTPQTPPDQRFDQRQETRR
>AT5G44780
SFMPDNEGCDFNHWLITMNFPKDNLPSREEMISIFEQTCAKGLASLEEAKKKIYAICTTSYQGFQATMTIGEVEKFRD
LPGVQYIIPDSYIDVENKVYGGDKYENGVIVPVPTKGFDSLKKESKEIILTPLTLPDQRVKQRQEMGQ
>GSVIVT01016740001
RLPTILDGCDYEHWLVVMEAP-QRYPLRDEIVRGYIRTLAMVLRSEEEAKKSIYSVSTKYYYAFGCKIAENLAHQIKS
LPNVKWVLPDSYLCHGGNGYGGEPFVNGEVVPYDEKDKSDDKCRNKTSSKKARRKRKNRFSKDQDV--
>Al_877351
RVSSLVEGCDYKHWLVLMKPP-NRYPTRNHIVQRFVETLAMALGSEEEAKKSIYSVSTKYYYAFGCRVHEPLTYKIRS
LPDVKWVLPDSYIVDGDNRYGGEPFVDGEVVPYDEKDQTDDDANNRVVKKKPRRKTLI----------
>AT1G72530
---SLVEGCDYKHWLVLMKPPNG-YPTRNHIVQSFVETLAMALGSEEEAKRSIYSVSTKYYYAFGCRIHEPLTYKIRSLP
DVKWVLPDSFIVDGDNRYGGEPFVDGEVVPYDEKDQTDEDA----------KSGVVKKKHRRKRKK
>MA_48983p0010
RRESLFPGCDYEHWLVTMEFP-DPQTTREQKIDTFVKTLANVVGSEEEAKKRIYALSTTTYTGFMCEISEELSEKIKKE
PGVEWVLPDSYGDPIKKEYGGDKYINGVIIPYNRPNRRRDSPIERRDVQTSRDDQRDFPTESRDSGQ
>MA_15760p0010
RREPLFPGCDYEHWLVTMEFP-EPQPSREEKIDTFVKTLANIVGGIDEAKKRIYALSTSTYTGFMCEISEELSEKIKKEP
GVVWVLPDSYADPLKKEYGGDKYINGVIIPYNRPRRRDSMPLERRDVQIPRDQGVQRYRPPMDGQD
>Potri.004G218800.1
ENKTLFEGCAYNYWLVTVDFPKEEPKSPREMIAAYERICAQGLNSIEEAKKRIYACSTTTFQGFQVLMTEQESEKFRD
VPRVVFVLPDS---PGNKEYEGDEYEHRMITPGPTTRDQRIPPFDQRDSPIPQNSQQGRYGSQQNGPP
>Al_492871
EDTVLFEGCDYNHWLITMDFSKEETRSPEEMVSAYEETCALGLGSVEEAKKRMYACSTTTYQGFQAIMTEQESEKF
KDLPGVVFILPDSYIDPQNKEYGGDKYENGVITHRPPPQSGRTRPRPRGGGGGFQNFQRNTYGQQPPMGG
>AT4G20020
EDTVLFEGCDYNHWLITMDFSKEETPSPEEMVAAYEETCAQGLGSVEEAKQRMYACSTTTYQGFQAIMTEQESEKF
KDLPGVVFILPDSYIDPQNKEYGGDKYENGVITHRPPPQSGRARPRPRGGSGGPQNFQRNTYGQQPPMGG
>Potri.004G218600.1
KNTILFEGNEYIHWLVTVDFPKEPKPSPEEMVAAFERICAQGLNSIEEAKKRMYACSTTIYQGFQVSITHQEAEKFRG
RPGAVFVSPDSRVKKEN---GGDKYKNAVITPRPPPRDPRIPPFDQPESPIPNHQGPQPYSQQGHMGS
>Potri.003G015100.1
EDTILFPGCDYNHWLITVDFPKDPKPSPEEMVATYERICAQGLNSIEEAKKKIYACSTTTYQGFQALMSEQESEKFKD
VPGVVFVLPDSYIDPVNKEYGGDKYENGVITPRPPPGERRYERFNQQGGPMPNHQGPPPHGQQGHMGS
>MA_489006p0010

DTTILFEGCDYEHWLITMEFP-DPQPTREEKIDTFIKTLAKVVGSEDEAKKRIYALSTTTYTGFQAQISEELSEKMKGL
PGVVWVLPDSYIDPVNKEYGGDKYINGVIIPRPSGNRMRRDPVERPIDGRVGMQGDGRFRPPMEGMP
>Bradi4g22160.1
PDEILFEGCDYNHWLITMEFP-DPKPSREEMIETFLQTLAQVVGSYEEAKKRMYALSTTTYVGFQAEITEEMSEKFRG
MPGVVFILPDSYLYPETKEYGGDKYDNGVITPRPPPKPQRTD-RNRNYQNSPQNSPPPPYSAHQDRAP
>LOC_Os11g11020
PDEILFEGCDYNHWLITMEFP-DPKPTREEMIETYLQTLAKVVGSYEEAKKRMYAFSTTTYVGFQAVMTEEMSEKFR
GLPGVVFILPDSYLYPETKEYGGDKYENGVITPRPPPKPSRTD-RNRNYQNGPNYQNSPPYGSQQDGAP
>ZmDAL2
PDEILFEGCDYNHWLITMDFP-DPKPSREEMIETYLQTLAKVVGSYEEAKKRMYAFSTTTYVGFQAVMTEEMSEKFR
GLPGVVFILPDSYLYPETKEYGGDKYDNGVITPRPPPRPSRTD-RNRNYQDGPGYQNNPPYRSQQDGAP
>Sobic.005G100900
PDEILFEGCDYNHWLITMEFP-DPKPSREEMIETFLQTLAKVVGSYEEAKKRMYAFSTTTYVGFQAVMTEEMSEKFK
GLPGVVFILPDSYLYPETKEYGGDKYDNGVITPRPPPRTDRNRNYRGNYQDGPQNNPPQQFQTNRSQRG
>GSVIVT01023346001
-----------------MDFPKDPKPTPEEMVETYVQTLAKGLNSVEEAKLKMYACSTTTYTGFQAVMTEEESEKFRGLPGVVFI
LPDSYINPATKEYGGDKYINGTIIPRPPPQYGRTGG---RYGDRNRNTERPRYDRQGELRT
>Aquca_095_00030
-----------------MDFPKDPKPTPEQMVETYVHTLAQVVGSVEEAKKKMYACSTTTYQGFQAEITEEESEKFRGLPGVVFI
LPDSYVDPVNKEYGGDKYINGTIIPRPPPRQGRYNDRNRTGYDRPRPSDDRRFGQGGERGP
>Aquca_039_00101
PDTILFEGCDYKHWLITMDFPKDPAPSPEQMVETYVNTLAQVVGSVEEAKKKMYACSTTTYHGFQAEISEEESEKFK
GLPGVVFILPDSYIDTVNKEYGGDKYINGTIIPRPPPQRGRYGDRNRTGYDRPRPSDDRQFGQEGERGP
>Al_902623
EMAPLFPGCDYEHWLIVMEKPGGENAQKQQMIDCYVQTLAKIVGSEEEAKKKIYNVSCERYFGFGCEIDEETSNKL
EGLPGVLFVLPDSYVDPEFKDYGAELFENGEVVPRPPERQRRMVETTQRGSDKPKYHDRTRNVRRRENMR
>Al_921093
EMAPLFPGCDYEHWLIVMEKPGGENAQKQQMIDCYVQTLAKIVGSEEEAKKKIYNVSCERYFGFGCEIDEETSNKL
EGLPGVLFVLPDSYVDPEFKDYGAELFENGEVVPRPPERQRRMVETTQRGSDKPKYHDRTRNVRRRENMR
>AT2G35240
EMAPLFPGCDYEHWLIVMEKPGGENAQKQQMIDCYVQTLAKIVGSEEEARKKIYNVSCERYFGFGCEIDEETSNKL
EGLPGVLFVLPDSYVDPEFKDYGAELFVNGEVVPRPPERQRRMVETNQRGSDKPKYHDRIRNVRRRENMR
>Al_473448
EMAPLFPGCDYEHWLIVMDKPGGENATKQQMIDCYVQTLAKILGSEEEAKKKIYNVSCERYFGFGCEIDEETSNKFE
GLPGVLFVLPDSYVDQENKDYGAELFVNGEIVQRPPERQRKIIETTQRSNDKPKYHDKTRYVRRRENMR
>AT1G32580
EMAPLFPGCDYEHWLIVMDKPGGENATKQQMIDCYVQTLAKIIGSEEEAKKKIYNVSCERYFGFGCEIDEETSNKLE
GLPGVLFILPDSYVDQENKDYGAELFVNGEIVQRPPERQRKIIETTQRTNDKPKYHDKTRYVRRRENMR
>Bradi1g50640.2
EMAPLFPGCDYEHWLIVMDKPGGEGATKQQMIDCYIQTLAKILGSEEEAKKKIYNVSCERYFGFGCEIDEETSNKLE
GIPGVLFVLPDSYVDPENKDYGAELFVNGEIVQRSPERQRRVEPVPQRASNRPRYNDRTRYARRMENQR
>Bradi1g42860.2
EMAPLFPGCDYEHWLIVMDKPGGEGATKQQMIDCYIQTLAKILGSEEEAKKKIYNVSCEQYFGFGCEIDEETSNKLE
GIPGVLFVLPDSYVDPENKDYGAELFVNGEIVQRSPERQRRVEPVPQRASDRPRYNDRTRYAWRRENQR

>Bradi3g00440.1
EMAPLFPGCDYEHWLIVMDKPGGEGATKQQMIDCYIQTLAKILGSEEEAKKKIYNVSCERYFGFGCEIDEETSNKLE
GIPGVLFVLPDSYVDPENKDYGAELFVNGEIVQRSPERQRRVEPVPQRASDRPRYNDRTRYARRRENQR
>GSVIVT01028387001
-MAPLFPGCDYEHWLIVMDKPGGEGATKHQMIDCYIQTLAKVVGSEEEAKKKIYNVSCERYFGFGCEIDEETSNKLE
DLPGVLFVLPDSYVDPENKDYGAELFVNGEIVQRSPEQQRRVEPQPQTGRDRPKYNDRTRYVRRRENMR
>Sobic.010G013900
DMAPLFPGCDYEHWLIVMDKPGGEGANKQQMIDCYIQTLAKVLGSEEEAKRKIYNVSCERYFGFGCEIDEETSNKL
EGLPGVLFVLPDSYVDPEYKDYGAELFVNGEIVQRPPERQRRVEPVPQRSADRPRYNDRTRYARRRENQR
>ZmDAL3
EMAPLFPGCDYEHWLIVMDKPGGEGATKQQMIDCYIQTLAKVLGSEEEAKKKIYNVSCERYFGFGCEIDEETSNKLE
GLPGVLFVLPDSYVDPEYKDYGAELLVNGEIVQRPPERQRRVEPVPQRAADRPRYNDRTRYARRRENQR
>ZmDAL4
EMAPLFPGCDYEHWLIVMDKPGGEGASKQQMIDCYIQTLAKVLGSEEEAKKKIYNVSCERYFGFGCEIDEETSNKLE
GLPGVLFVLPDSYVDAEYKDYGAELFVNGEIVQRTPERQRRVEPVPQRAADRPRYNDRTRYARRRENQR
>Al_482286
EMAPLFPGCDYEHWLIVMDKPGGEGATKQQMIDCYIQTLAKVVGSEEEAKKRIYNVSCERYLGFGCEIDEETSTKLE
GLPGVLFVLPDSYVDPENKDYGAELFVNGEIVQRSPERQRRVEPQPQRAQDRPRYNDRTRYSRRRENTR
>AT2G33430
EMAPLFPGCDYEHWLIVMDKPGGEGATKQQMIDCYIQTLAKVVGSEEEAKKRIYNVSCERYLGFGCEIDEETSTKLE
GLPGVLFVLPDSYVDPENKDYGAELFVNGEIVQRSPERQRRVEPQPQRAQDRPRYNDRTRYSRRRENTR
>ZmDAL1
EMAPLFPGCDYEHWLIVMDKPGGEGATKQQMIDCYIQTLAQVVGSEEEAKKRIYNVSCERYFGFGCEIDEETSNKLE
GLPGVLFVLPDSYVDAENKDYGAELFVNGEIVQRSPERQRRVEPVPQRAQDRPRYSDRTRYVKRRENQR
>Sobic.006G204100
EMAPLFPGCDYEHWLIVMDKPGGEGATKQQMIDCYIQTLAQVVGSEEEAKKRIYNVSCERYFGFGCEIDEETSNKLE
GLPGVLFVLPDSYVDAENKDYGAELFVNGEIVQRSPERQRRVEPVPQRAQDRPRYSDRTRYVKRRENQR
>Bradi5g20660.1
EMAPLFPGCDYEHWLIVMDKPGGEGATKQQMIDCYIQTLAKVVGSEEEAKKKIYNVSCERYFGFGCEIDEETSNKL
EGLPGVLFVLPDSYVDAENKDYGAELFVNGEIVQRSPERQRRVEPVPQRAQDRPRYSDRTRYVKRRENQR
>LOC_Os04g51280
EMAPLFPGCDYEHWLIVMDKPGGEGATKQQMIDCYIQTLAKVVGSEEEAKKKIYNVSCERYFGFGCEIDEETSNKL
EGLPGVLFVLPDSYVDAENKDYGAELFVNGEIVQRSPERQRRVEPVPQRAQDRPRYSDRTRYVKRRENQR
>Potri.010G068300.1
EMAPLFPGCDYEHWLIVMDKPGGEGATKQQMIDCYIQTLSKVVGSEEEAKNKIYNVSCERYFGFGCEIDEETSNKLE
GLPGVLFVLPDSYVDPEYKDYGAELFVNGEIVQRPPERQKRVEPQPQRANDRPRYNDRTRYVRRRENMR
>Potri.008G169900.1
EMAPLFPGCDYEHWLIVMDKPGGEGATKQQMIDCYIETLAKVVGSEEEAKTKIYNVSCERYFGFGCEIDEETSNKLE
GLPGVLFVLPDSYVDPEYKDYGAELFVNGEIVQRPPERQRRVEPQPQRANDRPRYNDRTRYVRRRENMR
>LOC_Os06g02600
EMAPLFPGCDYEHWLIVMDKPGGEGATKQQMIDCYIQTLAKVLGSEEEAKKKIYNVSCERYFGFGCEIDEETSNKLE
GLPGVLFVLPDSYVDPEYKDYGAELFVNGEIVQRSPERQRRVEPVPQRASDRPRYNDRTRYARRRENQR
>GSVIVT01027872001
------------------------------MIDCYIQTLAKVVGSEEEAKKKIYNVSCERYFGFGCEIDEETSNKLEGLPGVLFVLPDSYVD

PEYKDYGAELFVNGEIVQRSPERQRRVEPAPQRAQDRPRYNDKTRYVRRRENMR
>MA_10293670p0010
KEFVLFEGCDYQHWLIVMDAPVGQ-VSREDLIAKYVRTLAIVMGSEEEAKKAIYSVSTRHYFAFGCKISEELSEKLKP
LPGVRFVLPDSYLDPRTKSYGGEPFINGEAVPYDE----KYH----------------AFNKRNH---
>Aquca_004_00329
KETILLDGCDFEHWLIVVENPADK-LTRDEIIDSYIKKLAQVFGNEEEARQKIYSVSTRHYFAFGCIVPEEISYKIKELPG
VRWVLPDSYLDVKNKDYGGEPFINGQAVPYDPKNKIDSENRGRRNKDRPRNFDRSRFERRRENIQ
>MA_10430373p0010
KETILLDGCDYEHWLIVLEPPEGN-PTRDEIIDSYIKTLSQVVGSEEEARMKIYSVSTKHYFAFGCLISEELSYKLKPMK
NVRWVLPDSYLDPRTKSYGGEPFINGQAVPYDPKNNARCN--ERRSNDRPRNFDRSRFERRREMAR
>Al_892358
KETILLDGCDFEHWLVVMEKPEGD-LTRDEIIDYYIKTLAQVVGSEEEARMKIYSVSHKCYFAFGALVSEDLSYKIKE
LPKVRWVLPDSYLDVKSKNYGGEPFIDGKAVPYDPKNNDSSNSRTR----RPRTLSGTRFERRRENVR
>LOC_Os09g04670
KETILLDGCDFEHWLVVMDPPPGDPSTRDEIIDGYIKTLAQIVGSEDEARHKIYSVSTRHYFAFGALVSEELSYKLKEL
PKVRWVLPDSYLDVRNKDYGGEPFINGEAVPYDPKNNARANERTRRN-DRPRNFDRSRFERRRENMH
>LOC_Os09g33480
KETILLDGCDFEHWLVVVEPPPGDPSTRDEIIDGYIKTLAQVVGSEEEARHKIYSVSTRHYFAFGALVSEELSYKLKEL
PKVRWVLPDSYLDVRNKDYGGEPFINGEAVPYDPKNNARANERSRRN-DRPRNFDRSRFERRRENMQ
>Sobic.001G485700
KEMILLDGCDFEHWLVVMEPPPGDPSPRDEIIDSYIKTLAQVVGSEEEARQKIYSVSTRHYFAFGALVPEEVSYKLKE
MPKVRWVLPDSYLNVQTKDYGGEPFVNGEAVPYDPKNNARANERSRRN-DRPRNFDRSRFERRRGNMQ
>Bradi3g41430.1
KETILLDGCDFEHWLVVMEPPPGDASTRDEIIDSYIKTLAQIVGSEEEAKQKIYSVSTRHYFAFGALVSEELSYKLKEL
PKVRWVLPDSYLDVRNKDYGGEPFINGEAVPYDPKNNARANERSRRT-DRPRNFDRSRFERRRENQQ
>ZmDAL5
KETILLDGCDFEHWLVIMEPPPGDASTRDEIIDSYIKTLAQVVGSEEEARQKIYSVSTRHYFGFGALVSEELSYKLKEIP
KVRWVLPDSYLDVKNKDYGGEPFINGQAVPYDPKNNARANDRNRRN-DRPRNFDRSRFDRRRENMQ
>Sobic.001G485600
KETILLDGCDFEHWLVVMEPPPGDASTRDEIIDSYIKTLAQIVGSEEEARQKIYSVSTRHYFAFGALVSEELSYKLKEM
PKVRWVLPDSYLDVKNKDYGGEPFINGEAVPYDPKNNARANERSRRN-DRPRNFDRSRFERRRENMQ
>Aquca_081_00020
KETILLDGCDFEHWLIVLEKPEGD-PTRDEIIDSYIKTLALVVGSEEEARMKIYSVSTRHYYAFGALVPEELSYKIKELP
RVRWVLPDSYLDVRNKDYGGEPFINGQAVPYDPKNNARAQDRSRRN-DRPRNFDRSRFERRRENMQ
>Al_478926
KETILLDGCDFEHWLVVVNPPEGD-PTRDDIIDSYIKTLAQIVGSEDEARMKIYSVSTRCYYAFGALVSEDLSHKLKEL
PNVRWVLPDSYLDVRNKDYGGEPFIDGKAVPYDPKNNARANERNRRN-DRPRNFDRTRFERRRENMA
>AT3G15000
KETILLDGCDFEHWLVVVEPPQGE-PTRDEIIDSYIKTLAQIVGSEDEARMKIYSVSTRCYYAFGALVSEDLSHKLKEL
SNVRWVLPDSYLDVRNKDYGGEPFIDGKAVPYDPKNNARANERNRRN-DRPRNNDRSRFERRRENMA
>Potri.001G393400.1
KETILLDGCDFEHWLVVMEKPEGD-PTRDEIIDSYIKTLAQVVGSEEEARRKIYSVSTRCYYAFGALVPEEVSYKIKEL
KNVRWVLPDSYLDVKNKDYGGEPFIDGKAVPYDPKNNARANERNRRN-DRPRNVDRSRFDRRMENMQ
>GSVIVT01014223001

-----------------MEKPEGD-PTRDEIIDSYIKTLAMIVGSEEEARMKIYSVSTRCYFAFGALVSEELSLKIKELPRVRWVLPD
SYLDVKNKDYGGEPFIDGKAVPYDPKNNARANERNRRN-DRPRNFDRSRFERRRENMQ
>Potri.011G112200.1
KETILLDGCDFEHWLVVMDKPEGD-PTRDEIIDSYIKTLAEVVGSEEEARKKIYSVSTRCYFAFGALVSEEVSYKIKEL
KNVRWVLPDSYLDVKNKDYGGEPFIDGKAVPYDPKNNARANERNRRN-DRPRNVDRSRFDRRRENMQ
>Aquca_003_00654
KETILLDGCDYEHWLVVMEFPQN--LSEDEMVSSYVNTLASVVGSVEEAKQKIYSVCTSTYTGFGALISEELSYKLKG
LPGVLWVLPDSYLDVPNKDYGGDLFVDGKVIPRP---QYRIDQRQQNRGNRP----RPRYDRRRETEK
>MA_5791p0010
KETILLDGCDYEHWLIVMEFSKDPKPPEEEMIAAYIKTLASVVGSEEEAKKKIYSVSTHTYTGFGALISEELSYKVKG
LPGVLWVLPDSYIDVPNKDYGGDLFVDGKVIPRP---QFRYPERQQGRNDRP----RPRYDRRRDSER
>Al_477997
KETILLDGCDYEHWLIVMEFT-DPKPTEEEMINSYVKTLTSVLGSEEEAKKKIYSVSTSTYTGFGALISEELSCKVKEL
PGVLWVLPDSYLDVPNKDYGGDLYIEGEVIPRP---QYRFTE---QRQTRNRY--RPRYDRRRETER
>AT3G06790
KETILLDGCDYEHWLIVMEFT-DPKPTEEEMINSYVKTLTSVLGWQEEAKKKIYSVCTSTYTGFGALISEELSCKVKA
LPGVLWVLPDSYLDVPNKDYGGDLYVEGKVIPRP---QYRFTE-QRHTRPRPR-----PYDRRRETER
>Potri.010G007200.1
KETILLDGCDYNHWLIVMEFPNDPKPTEEEMINAYVKTLSSVLGSEEEAKKSIYSVSTTTYTGFGALISEELSYKVKA
LPGVLWVLPDSYLDVPNKDYGGDLYEDGKVIHRP---QYRYNERQQ----QTRNRPRPRYDRRRETER
>GSVIVT01035101001
KETILLDGCDYEHWLIVMEFPNDSKPSEDEMIAAYVKTLAAVVGSEEEAKKKIYSVCTTTYTGFGALISEELSYKVKE
LPGVLWVLPDSYLDVPNKDYGGDLFIDGKVIHRP---QYRYNERQP-----TRSRPRPRYDRRRETMQ
>ZmDAL7
KETILLDGCDYEHWLIVMEFPTDPKPSEEEMVAAYVKTLAAVLGSEEEAKKKIYSVCTSTYTGFGALISEELSYKVKG
LPGVLWVLPDSYLDVPNKDYGGDLFVDGKVIHRP---QFRFNERQQ-----VRSRPRPRYDRRREIEP
>Sobic.006G216000
KETILLDGCDYEHWLIVMEFPTDPKPSEEEMVGAYVKTLAAVLGSEEEAKKKIYSVCTSTYTGFGALISEELSYKVK
GLPGVLWVLPDSYLDVPNKDYGGDLFVDGKVIHRP---QFRFNERQQ-----VRSKPRPRYDRRREVVQ
>Bradi2g31140.1
KETILLDGCDYEHWLIVMEFPTDPKPSEEEMVAAYVKTLTAVIGSEEEAKKKIYSVCTTTYTGFGALISEELSYKVKG
LPGVLWVLPDSYLDVPNKDYGGDLFIDGKVIHRP---QFQFTERQQ-----VRSRPRPRYDKRRETDR
>LOC_Os03g38490
KETILLDGCDYEHWLIVMEFPTDPKPSEEDMVAAYVKTLAAVVGSEEEAKKKIYSVCTTTYTGFGALISEELSYKVK
GLPGVLWVLPDSYLDVPNKDYGGDLFVDGQVIHRP---QFRFTERQQ-----VRSRPRPRYDRRRVTMQ
>MA_123833p0010
KETILLPGCDYEHWLIVMEFPKDPKPTSEEMVDTYIKTLAKVVGSEEEAKKKIYALSTTTYTGFQANISEELSEKCKG
LPGVLWVLPDSYIDVPNKDYGGDKFVDGKVIPRP---QPRPSERQTRS--SYNRTNRTRYERRRDGPR
>Al_471280
RETIMLPGCDYNHWLIVMEFPKDPAPTREQMIDTYLNTLATVLGSMEEAKKNMYAFSTTTYTGFQCTIDEETSEKFK
GLPGVLWVLPDSYIDVKNKDYGGDKYINGEIIPCT---YPTYQPKQR---NNTKY--QSKYERKRDGPP
>AT1G11430
RETIMLPGCDYNHWLIVMEFPKDPAPSRDQMIDTYLNTLATVLGSMEEAKKNMYAFSTTTYTGFQCTIDEETSEKFK
GLPGVLWVLPDSYIDVKNKDYGGDKYINGEIIPCT---YPTYQPKQRNN-----TKYQSKYERKRDGPP

>Potri.011G032900.1
RETILLPGCDYNHWLIVMEFPKDPAPTREQMIDTYLNTLATVLGSMEEAKKNMYAFSTTTYTGFQCTVDEATSEKFK
GLPGVLWVLPDSYIDVKNKDYGGDKYVNGEIIPCT---YPTYQPKQR---TTSKY-ENRRYERRRDGPP
>Aquca_028_00257
RETILLPGCDYNHWLIVMEFPKDPAPTREQMIDTYLNTLATVVGSMEEAKKNMYAFSTTTYTGFQCTVSEETSEKFK
GLPGVLWVLPDSYIDVKNKDYGGDKYINGEIIPCK---YPTYQPKQR----SGSKYESKRYERRKSGEK
>ZmDAL6
RETILLPGCDYNHWLIVMEFPKDPAPTREQMIDTYLNTLATVLGSMEEAKKNMYAFSTTTYTGFQCTVDEETSEKFK
GLPGVLWVLPDSYIDVKNKDYGGDKYVNGEIIPCT---YPTYQPKER----RTSKYESRRYERRRDGAS
>LOC_Os08g04450
RETILLPGCDYNHWLIVMEFPKDPAPTREQMIDTYLNTLATVLGSMEEAKKNMYAFSTTTYTGFQCTVDEETSEKFK
GLPGVLWVLPDSYIDVKNKDYGGDKYINGEIIPCT---YPTYQPKERRT-----SKYESRYERRRDGPP
>Sobic.007G034500
RETILLPGCDYNHWLIVMEFPKDPAPTREQMIDTYLNTLATVLGSMEEAKKNMYAFSTTTYTGFQCTVDEETSEKFK
GLPGVLWVLPDSYIDVKNKDYGGDKYINGEIIPCT---YPTYQPKERRT-----SKYESRYERRRDGPP
>Bradi3g14650.1
RETILLPGCDYNHWLIVMEFPKDPAPTREQMIDTYLNTLATVLGSMEEAKKNMYAFSTTTYTGFQCTVDEETSEKFK
GLPGVLWVLPDSYIDVKNKDYGGDKYINGEIIPCT---YPTYQPKERRT-----SKYESRYERRRDGPP
>GSVIVT01014547001
------------------------------MIDTYLNTLATVLGSMEEAKKNMYAFSTTTYTGFQCTVSEETSEKFKGLPGVLWVLPDSYI
DVKNKDYGGDKYINGEIIPCT---YPTYQPKQR----RESKYESRRYERRRDGEK

**Supplemental Data File S4. Alignment of complete protein sequences of plant *DAL* genes.fas**

>Al_877351
----------------------------MARIIRRPLNLTAAVRFR--------------
-----------------------LAPLSPFSGNSGSVNSGTTRC---------------S
ELIRVSSLVEGCDYKHWLVLMKPPNRYP------TRNHIVQRFVETLAMAL-GSEEEAKK
SIYSVSTKYYYAFGCRVHEPLTYKIRSL----PDVKWVLPDSYIVDGDNRYGGEPFVDGE
VVPYD-----------------EKYHADWLRDQTDDDAN---------------NRVVKK
KPR--------------R------------------------------------------
---------------------------------------------KTKKKLI--------
------------------------------------------------------------
------------------------------------------------------------
------------------------------------------------------------
------------------------------------------------------------
------------------------------------------------------------
------------------------------------------------------------
-------------------------------------------------------

>AT1G72530
----------------------------MARIIRRPLNLTAAVRFRLSPLS---PFSGNS
GSINSET-----------------------------------------TSWSE-------
-LIRVPSLVEGCDYKHWLVLMKPPNGYP------TRNHIVQSFVETLAMAL-GSEEEAKR
SIYSVSTKYYYAFGCRIHEPLTYKIRSL----PDVKWVLPDSFIVDGDNRYGGEPFVDGE
VVPYD-----------------EKYHADWLRDQTDEDAK---------------SGVVKK
KHR--------------R------------------------------------------
---------------------------------------------KRKKKLI--------
------------------------------------------------------------
------------------------------------------------------------
------------------------------------------------------------
------------------------------------------------------------
------------------------------------------------------------
------------------------------------------------------------
-------------------------------------------------------

>Potri.003G015100.1
----------------------MAL---RLLRLRRTLTPLSSTLQR--PFSIP-VPIAPP
AAQSPTI---------------ISRSRVFTGTRVSMMSTTTARPEKKYKLYED-----GD
EITEDTILFPGCDYNHWLITVDFPKDPK----P-SPEEMVATYERICAQGLNISIEEAKK
KIYACSTTTYQGFQALMSEQESEKFKDV----PGVVFVLPDSYIDPVNKEYGGDKYENGV
ITPRP-----------------PPVHRG-GERRYE--------------------RNR-S
PPR--------------F------------------------------------------
---------------------------------------------NQQGGPMPNHQ----
------------------------------------------------------------
--------------------GPPPQHGQQGHMQGGGSNYGPQQN-YPPQQNRGPPGPGGS

MPMINRDHAPGGRNTNQGQQGNLYPPAQQAYNPGQHGNHYPPGQQGYNQGQQGNLYPPGQ
QGYNQGQHGNHYAPD---------------------------------------------
----QRSFLQGDPRDHGSPGQRDYRGGDRNYSPTHAGNYGQGGNTGIGQRHLGDGQKSAQ
MEQMSTQGEQGNYAPTGQPGWSDQVRQPPVRNYGQGGNTGYGQHNPGDGLRSAQMEQRST
QEEQGNYAPPGKPGWSDQGGY-----------------------------------
>Potri.004G218800.1
------------------------------MTSTAAIAEKQYWVY--------------
------------------------------------------------ENLGE-------
-ITENKTLFEGCAYNYWLVTVDFPKEEP----KPSPREMIAAYERICAQGLNSSIEEAKK
RIYACSTTTFQGFQVLMTEQESEKFRDV----PRVVFVLPDS---PGNKEYEGDEYEHRM
ITPGP-----------------TTVQFQRGGERCR--------------------DQG-R
IPP------------R-F------------------------------------------
---------------------------------------------DQRDSPIPNNQ----
------------------------------------------------------------
--------------------GLQPQNSQQGRMPGGGGNYGSQQN-GPPQQNHGPPGPGGR
MPMNNRDYAPGGRNMY----------------PGQEGNHDPPGQQGYNHGQQRNHYPPGG
GGNHDPPGQQGYNHG---------------------------------------------
-----------QQRNHYPQGQGYNQGQQGNHYAPDQRSFP--------------------
-----------------QGDWRDHGPPGQRDYKGDNWNYSPTHGGNYGQGGNSSYGQRNP
GECQRSAQMEQMGMQGEQGNYAPLGPPGWSNQVRHTPH-----------------
>Potri.004G218600.1
----------------------MAQ---RLLRLRRALTPFSSTRQR-PPLSTP-IAIAPP
AAQTPPI------------ISQWRGFSGTRVSMMSTTGLAEKQY----KVYED-----GE
EIVKNTILFEGNEYIHWLVTVDFPKEPK----P-SPEEMVAAFERICAQGLNISIEEAKK
RMYACSTTIYQGFQVSITHQEAEKFRGR--CVPGAVFVSPDSRVKKEN---GGDKYKNAV
ITPRP-----------------PPVQFQRGGERRR--------------------DPG-R
IPP------------R-F------------------------------------------
---------------------------------------------DQPESPIPNHQ----
------------------------------------------------------------
--------------------GPQPQYSQQGHMQGGGSNYGSQQN-RPPQKNHGPPGLGGT
VPMNNRDYATGGRNTY----------------PGQQGNHDPPGQQGYNQGQQGNHYDPDQ
RSFPQGDWR---------------------------------------------------
-------------RDHGSPGQRDYRGDNWNYSPTHGGN----------------------
-----------------YGQGGNTSYGQRHPGEGQRSAQMELRGMQGEQGNYAPMGQPGW
SNQIIHRWFHSAGEDGEWFTDLICTHDL---------------------------
>Al_492871
----------------------MAM---LSHRIRRALLTATSYVNR-SIGTSI-APASDF
PSVSAAV------------LQRSVLGRSTEVATRAPARLYSTRQY----KLYKE-----GD
EITEDTVLFEGCDYNHWLITMDFSKEET----RKSPEEMVSAYEETCALGLGISVEEAKK
RMYACSTTTYQGFQAIMTEQESEKFKDL----PGVVFILPDSYIDPQNKEYGGDKYENGV
ITHRP-----------------PPIQSGRTRPRPR-------------------------
-----------------F------------------------------------------
---------------------------------------------DRSGGGGGGFQ----
------------------------------------------------------------

------------------NFQRNTQYGQQPPMQGGGGSFGPQQGYGTPGQGQGTQAPPPF
QGGY----------------------------NQGPGSPPPPYQASYNQGQGSPVPPYQG
PQSSYGQGGSGNYSQ---------------------------------------------
-----------------GPQGGYNQGGPRNYSPQGAGNFG--------------------
-----------------PAPGAGNFGPAPGTGNPGYGQGYSGPGQEQNQTFPQANQRNPD
WNNNNPAGQPGSDQFPQGRRY----------------------------------
>AT4G20020
----------------------MAM---ISHRLRRALLTATSYVNRSISSSITPASDFPS
VSAAVLK-------------RSVIGRSTEVATRAPARLFSTRQY----KLYKE-----GD
EITEDTVLFEGCDYNHWLITMDFSKEET----PKSPEEMVAAYEETCAQGLGISVEEAKQ
RMYACSTTTYQGFQAIMTEQESEKFKDL----PGVVFILPDSYIDPQNKEYGGDKYENGV
ITHRP-----------------PPIQSGRARPRPR-------------------------
-----------------F------------------------------------------
---------------------------------------------DRSGGGSGGPQ----
------------------------------------------------------------
------------------NFQRNTQYGQQPPMQGGGGSYGPQQGYATPGQGQGTQAPPPF
QGGYNQGPRSPPPPYQAGY-------------NQGQGSPVPPYQAGYNQVQGSPVPPYQG
TQSSYGQGGSGNYSQ---------------------------------------------
-----------------GPQGGYNQGGPRNYNPQGAGNFG--------------------
-----------------PASGAGNLGPAPGAGNPGYGQGYSGPGQEQNQTFPQADQRNRD
WNNNNPAGQPGSDQVRSRSIMNLASFFFDILIRH---------------------
>Al_494504
----------------------MAM---LSHRLRRVLVAAPSYFQRSFPLSHP-SDFPPV
SSLLPRS----------------VVKQSTEFGKSPARLFSTTQYQYD-PYTGE------D
SFMPDN---EGCDFNHWLITMNFPKDNV----P-SREEMISIFEQTCAKGLDISLEEAKK
KIYAICTTSYQGFQATMTIGEVEKFRDL----PGVQYIIPDSYADVENKVYGGDKYENGV
ITPGPIPVPTKEGFDSLEKESKPEQEEAQIIQTPSDEGKISGQVQDQGSQTPPDQRSVKR
MQGAIDLGQGQGQRSR-MSIPGQGQGEGSRMSIPGQGHSRGQGNRMPFMQGGFKQSQGTL
SIGQGKTQRSEMPSFQGNVKQGEEIPINGQWQAPRSQMPSSQGSFDQRQETPTLRR----
-GHAQGSQIPSFQSGNNQSQGTPIPGQGQGSQIPSNQVGYNQGQGAQTPPYQGLPNNYGQ
GAFVQYNQEPPQGNFIQGTQENYNQMGQRSYTPQSGGNYGPSQGAGSPGFG-HGQGQGGH
LLSPYQGSYNQGQGTPLT--------------GQGQEGQIPSYHMGNSQGLGAPVPPNQV
TPGNYGQWAFVNYNQGPPHGNFLQGPQQNYNQGGQWNYSPHNGGHYGPAQFGQWYHGPPQ
GQGNQWPQYQLSYNQGQGTPFPGQCLCPGCGMPSYQGSYNQGQGTHI--HGQFQGQGCAM
PSYQASYSHSQGVPVPLVPPYHGNYNPGPPSSYGQGTSTNINQGFPVNPANYNMQNGGNY
GPPHELAGNPGFRQGFTGQGQNQTFQQEDQRNVAGGLSNNNPADPTVTRKPNSRT
>AT5G44780
----------------------MAM---FSHRLRRIVVAAPSYFQRFSTLSRP-SDFTPV
PSLLPRS----------------VVKQSTAINRSPARLFSTTQYQYD-PYTGE------D
SFMPDN---EGCDFNHWLITMNFPKDNL----P-SREEMISIFEQTCAKGLAISLEEAKK
KIYAICTTSYQGFQATMTIGEVEKFRDL----PGVQYIIPDSYIDVENKVYGGDKYENGV
ITPGPVPVPTKEGFDSLKKESKPEQEEAEIILTPPDEGKTSGQVQGQGSLTLPDQRSVKE
RQGTLALVQGQGQRSG-MSILGQGQGEGRRMSIPGQWQSRGQGN----------------
-------------SFQGSFKQSQGTLPVRKGQTQISDEIPSFQGNVKQRQEMPIHGQGQA

QRSQMPSSQGTLRQGQAQGSQRPSNQVGYNQGQGAQTPPYHQGQGAQTPPYQESPNNYGQ
GAFVQYNQGPPQGNVVQTTQEKYNQMGQGNYAPQSGGNYSPAQG-AGSPRFGYGQGQGGQ
LLSPYRGNYNQGQGTPLP--------------GQGQEGQ-PSYQMGFSQGLGAPVPPNQV
IPGNYGQWAFVNYNQGPPQGNFLQGPQQNHNQGGQWNYSPQNGGHYGPAQFGQWYPGPPQ
GQGIQWPQYQLSYNQGQGTPFSGQCRCPNCGMTSYQGYNNQGQGTHIPEQW--EGQDYAV
LSYQASYNQAHGAQ---APPYHGNYNQATPGGYGQGTSANFNQRFPVNPANYNMQNGGNY
GPPHGLAGNPGFRQGFSGQGQNQTFQQDDQRNVAGDLRNNNPVDPTETRKPNSRI
>Aquca_003_00654
----------------------------MAFLTRRTLATFITRTLSSPSSSSS-SSSSLV
RSRFSLA-------ILDKQIVPESKVFQTTQLRTFGSGYSPLNDPS--PNWSN-RPP---
---KETILLDGCDYEHWLVVMEFPQNL-------SEDEMVSSYVNTLASVV-GSVEEAKQ
KIYSVCTSTYTGFGALISEELSYKLKGL----PGVLWVLPDSYLDVPNKDYGGDLFVDGK
VIPRP-----------------QYR----IDQRQQ--------------------NRG-N
RPRPR------------Y------------------------------------------
---------------------------------------------DRRRETMQTEK----
------------------------------------------------------------
------------------------------------------------------------
--------------------------------------REPMQRGNWAHDQRGPVSPPTA
------------------------------------------------------------
------------------------------------------------------------
-----------------VTGQNSFNGEGQNSFNSGPRESYPNHGDYQRGGA---------
-------------------------------------------------------
>ZmDAL7
----------------------MAA---ASATRRGLSSLLLSPSSRALRRRLG-PLAAAA
ASPHVAP-------------WPLLAPRGARTASSGGSGHSPLNDPS--PNWSN-RPP---
---KETILLDGCDYEHWLIVMEFPTDPK----P-SEEEMVAAYVKTLAAVL-GSEEEAKK
KIYSVCTSTYTGFGALISEELSYKVKGL----PGVLWVLPDSYLDVPNKDYGGDLFVDGK
VIHRP--------------------QFRFNERQQV--------------------RSR-P
RPR--------------Y------------------------------------------
---------------------------------------------DRRREIAQVEP----
------------------------------------------------------------
------------------------------------------------------------
--------------------------------------RETMQRGPSTTQQQRPPCPQEA
ATQNQEQH----------------------------------------------------
------------------------------------------------------------
------------------------------------------------------------
-------------------------------------------------------
>Sobic.006G216000
-----------------------MA---AASATRRGLSALLLYSSRALPRRLV-PLAASA
ASAHVAP-------------WALLASRGARTASSGGSGYSPLNDPS--PNWSN-RPP---
---KETILLDGCDYEHWLIVMEFPTDPK----P-SEEEMVGAYVKTLAAVL-GSEEEAKK
KIYSVCTSTYTGFGALISEELSYKVKGL----PGVLWVLPDSYLDVPNKDYGGDLFVDGK
VIHRP--------------------QFRFNERQQV--------------------RSK-P
RPR--------------Y------------------------------------------

---------------------------------------------DRRREVVQVER----
------------------------------------------------------------
------------------------------------------------------------
--------------------------------------RETMQRGPSTQQHRPPPFPQQA
TQNQEQH-----------------------------------------------------
------------------------------------------------------------
------------------------------------------------------------
-------------------------------------------------------

>Bradi2g31140.1
---------------------------MAAAGIRRRISELLLSSRAPHRRFLP-LAAAAV
SSAHLAP--------------WAPPSRGAKTALPGKSGYSPLNDPS--PNWSN-RPP---
---KETILLDGCDYEHWLIVMEFPTDPK----P-SEEEMVAAYVKTLTAVI-GSEEEAKK
KIYSVCTTTYTGFGALISEELSYKVKGL----PGVLWVLPDSYLDVPNKDYGGDLFIDGK
VIHRP--------------------QFQFTERQQV--------------------RSR-P
RPR--------------Y------------------------------------------
---------------------------------------------DKRRETMQADR----
------------------------------------------------------------
------------------------------------------------------------
----------------------------------REVMKNGPSIQEQRPPFSGEVAHNPE
QQHAVVLPGGN-------------------------------------------------
------------------------------------------------------------
------------------------------------------------------------
-------------------------------------------------------

>LOC_Os03g38490
------------------------MAAGAAAATRRSLSALLLSSRALQRRFAPLAAAASS
AYLAPWA----------------PPSRGAKTASSGGSGYSPLNDPS--PNWSN-RPP---
---KETILLDGCDYEHWLIVMEFPTDPK----P-SEEDMVAAYVKTLAAVV-GSEEEAKK
KIYSVCTTTYTGFGALISEELSYKVKGL----PGVLWVLPDSYLDVPNKDYGGDLFVDGQ
VIHRP--------------------QFRFTERQQV--------------------RSR-P
RPR--------------Y------------------------------------------
---------------------------------------------DRRRVTMQTEQ----
------------------------------------------------------------
------------------------------------------------------------
------------------------------------------------KEAMQKGPSNLQ
Q-----------------------------------------------------------
------------------------------------------------------------
------------------------------------------------------------
-------------------------------------------------------

>Potri.010G007200.1
-----MAYITARRN--------------LATLLTRALSSSSSRTRFSPALFNK-IQTSLI
PDSVKTL----------------------TRSKTTGSGYSPLNDPS--PNWTN-RPP---
---KETILLDGCDYNHWLIVMEFPNDPK----P-TEEEMINAYVKTLSSVL-GSEEEAKK
SIYSVSTTTYTGFGALISEELSYKVKAL----PGVLWVLPDSYLDVPNKDYGGDLYEDGK
VIHRP-----------------QYR----YNERQQ--------------------QTR-N

RPRPR------------Y------------------------------------------
---------------------------------------------DRRRETMQVER----
------------------------------------------------------------
------------------------------------------------------------
-------RETVQRQNWSQ--------------DPRPPVKQPASDNVQNSTQGGGGEFSMN
QGQFNQST----------------------------------------------------
------------------------------------------------------------
------------------------------------------------------------
-------------------------------------------------------

>Al_477997
-----MALINA-----------RRT---VATLLSKTLSSSSSSSSS-FSTLSS-RSRFAV
PLIEKVS--------------GLGPCYISTRLKTSGSGYSPLNDPS--PNWSN-RPP---
---KETILLDGCDYEHWLIVMEFTDPKP------TEEEMINSYVKTLTSVL-GSEEEAKK
KIYSVSTSTYTGFGALISEELSCKVKEL----PGVLWVLPDSYLDVPNKDYGGDLYIEGE
VIPRP--------------------QYRFTEQRQT--------------------RNR-Y
RPR--------------Y------------------------------------------
---------------------------------------------DRRRETMQVER----
------------------------------------------------------------
------------------------------------------------------------
----------------------------------------REPPMGHQAPAYPGEFNKPS
A-----------------------------------------------------------
------------------------------------------------------------
------------------------------------------------------------
-------------------------------------------------------

>AT3G06790
-----MALIST-----------RRT---LSTLLNKTLSSSTSYSSSFPTLSSRSRFAMPL
IEKVSSS------------RTSLGPCYISTRPKTSGSGYSPLNDPS--PNWSN-RPP---
---KETILLDGCDYEHWLIVMEFTDPKP------TEEEMINSYVKTLTSVL-GWQEEAKK
KIYSVCTSTYTGFGALISEELSCKVKAL----PGVLWVLPDSYLDVPNKDYGGDLYVEGK
VIPRP--------------------QYRFTEQRHT--------------------RPR-P
RPR--------------Y------------------------------------------
---------------------------------------------DRRRETMQVER----
------------------------------------------------------------
------------------------------------------------------------
-------------------------------REPSMGLHSPVNPGEFNKPSA--------
------------------------------------------------------------
------------------------------------------------------------
------------------------------------------------------------
-------------------------------------------------------

>Aquca_004_00329
----------------------MAMAFIARNLIKKPNPNALPSIFCRSFTVKP-NLSISY
GVQRGPL-LVALTTEFHSLTSMVGVGLSKRSLSTHTAATSSLRDPS--PNWNN-RPP---
---KETILLDGCDFEHWLIVVENPADKL------TRDEIIDSYIKKLAQVFGGNEEEARQ
KIYSVSTRHYFAFGCIVPEEISYKIKEL----PGVRWVLPDSYLDVKNKDYGGEPFINGQ

AVPYD-----------------PKYHEQWVRNKIDSENR---------------GRRNKD
RPRNFD-------RSRNF------------------------------------------
---------------------------------------------ERRRENIQNNR----
------------------------------------------------------------
--------------DDRPPADSNRGGMPTPYVQNSGTNNSGMPA-PTANMQNNVPSHGDV
SPAPNVGYSNNMNMRNG---------------VPNNGDVPPRPNVGYSNMNTGNGMPNHG
GMAPNAGANFGYSNNM-------------------------------------------N
TGSGVPNYTGVPHGDAGRNAAYPNNMNMGNNAPNYTGMPPRNTSSSGGYSN--NMNSGGI
PNRDAGHLNNMPPPPNRDFQSWDASNNGGTQIYQNRRGPVPNRGYQQNSYTPPPNGNMGN
VPSGNVYQVRDVPGRDLPPQVPYQNYQ----------------------------
>Aquca_081_00020
MAMSFLGRTLITKQNPNHLIPSIIS---RSFYVKSNSVPRFVCSYSTSSSFLS-RVSRPL
ALLTNTS--------FSDVVVPRFPSMVGLRCLSTRASTSSLNDPT--PNWNN-RPP---
---KETILLDGCDFEHWLIVLEKPEGDP------TRDEIIDSYIKTLALVV-GSEEEARM
KIYSVSTRHYYAFGALVPEELSYKIKEL----PRVRWVLPDSYLDVRNKDYGGEPFINGQ
AVPYD-----------------PKFHEEWVRNNARAQDR---------------SRRN-D
RPRNFD-------RSRNF------------------------------------------
---------------------------------------------ERRRENMQNRD----
------------------------------------------------------------
-------------------------AQPPMQNSGANMGGPPGPNMQQPGLNMQQPGPGMH
HPGPNMQHPGQNMQQP----------------MQSNMGVPPNANMHNNVPNYAGVPSRDA
VPNAGYSNNVNMGNR---------------------------------------------
----------MPNYGPPSAGPTGGYTNNMNMGGMPNRDAGPSGGYPNSM-----------
-----------------PQYRDAPYNGGVQHQSRDGPGPMPNRDYQNSYSPSSNMGGAPS
NNAYQVRDMPGRDLPPMPNQNYQ--------------------------------
>LOC_Os09g04670
------------------------MVSASRFVLLSRLPAAAASSRFLRPLAAA-GSLLPA
ALAPFAP---------------PAAGARCFATQPA---TSSLRDSS--PNWSN-RPP---
---KETILLDGCDFEHWLVVMDPPPGDPSNPEP-TRDEIIDGYIKTLAQIV-GSEDEARH
KIYSVSTRHYFAFGALVSEELSYKLKEL----PKVRWVLPDSYLDVRNKDYGGEPFINGE
AVPYD-----------------PKYHEEWVRNNARANER---------------TRRN-D
RPRNFD-------RSRNF------------------------------------------
---------------------------------------------ERRRENMHNFQ----
------------------------------------------------------------
------------------NRDV------------------------PPGQGFNSPPPPGQ
GPVLPRDAPPMP--------------------------PPPSPPNPGAPPSYQPHAPNPQ
AGYTN-------------------------------------------------------
-----Y-QGGVPGYQGRAPGY---QGGNQEYRGPPPPPPSAYQGNNP-------GYQGGG
PGYHGGN----------PPPYQAGNPP--PYQAGNPVFAGGAPGYQGQGGN-PSYQQGSD
N-YNAGAPAYKRDEPGRNYQ-----------------------------------
>LOC_Os09g33480
----------------------MASASRFLLLSRLPAAAASSTSRLLRPLSAA-GSLLPA
ALAPSAP-------------RAAAAAARCFATQPA---TSSLRDSS--PNWSN-RPP---
---KETILLDGCDFEHWLVVVEPPPGDPSNPEP-TRDEIIDGYIKTLAQVV-GSEEEARH

KIYSVSTRHYFAFGALVSEELSYKLKEL----PKVRWVLPDSYLDVRNKDYGGEPFINGE
AVPYD-----------------PKYHEEWVRNNARANER---------------SRRN-D
RPRNFD-------RSRNF------------------------------------------
---------------------------------------------ERRRENMQNFQ----
------------------------------------------------------------
------------------NRDV------------------------PPGQGFNSPPPPGQ
GPVPPRDAPPMHHAQG------------------NV--PPPPPPNAG-PPNYQPHAPNPQ
-GYTN-------------------------------------------------------
-----YQQGGAPGYQGGPPGY---QGSNQGYQGPPPPPPSAYQGNNP-------GYQGGG
PGYQGGN----------PPPYQGGNP-------------GYAPGYHGQGGN-PSYQQGGD
N-YNAGVPAYERDGQGRNYQ-----------------------------------
>Bradi3g41430.1
-----MAMAARA----------------VLLSRLSPLPAAASRFVLLRPLAAA-ATLLPA
AAASTIP-----------AAAARGAAVRCFATQPA---TSSLRDSS--PNWSN-RPP---
---KETILLDGCDFEHWLVVMEPPPGDASNPEI-TRDEIIDSYIKTLAQIV-GSEEEAKQ
KIYSVSTRHYFAFGALVSEELSYKLKEL----PKVRWVLPDSYLDVRNKDYGGEPFINGE
AVPYD-----------------PKYHEEWVRNNARANER---------------SRRT-D
RPRNFD-------RSRNF------------------------------------------
---------------------------------------------ERRRENQQNFQ----
------------------------------------------------------------
-----------NRDAPPGGFNSPPGPPPPPGSFNSPPPPPGQGFNSPPPPPGQGFNSPPP
PPGQGFNSPPPPPGQ-----------------NRGMPPPPPPLHTAGGPPHYQSHMQNPQ
AAYTPGGSPHMPNPQ--------------------------------AGYTQGSQPGYAP
GGAQNYQQSGAPGYQGGPSGYQGNQGGPPGYQGNQGG-----------------------
-----------------PPGYQGSGNQGGPPGYQGGPGPAHPGSNPGYQGGNTPPHESHG
GRFYGNAPDRYNQQ-----------------------------------------
>Sobic.001G485700
-----MASAS------------------RALHLSRALQGGRRITTLLRPLAAT-ESLLPA
GAGASAP----------------GAGLRCFATQPA---TSSLRDSS--PNWINTRPS---
---KEMILLDGCDFEHWLVVMEPPPGDPSNPDI-PRDEIIDSYIKTLAQVV-GSEEEARQ
KIYSVSTRHYFAFGALVPEEVSYKLKEM----PKVRWVLPDSYLNVQTKDYGGEPFVNGE
AVPYD-----------------PKFHEEWVRNNARANER---------------SRRN-D
RPRNFD-------RSRNF------------------------------------------
---------------------------------------------ERRRGNMQNYQ----
------------------------------------------------------------
------------------NRDG------------------------PLAQGFNGPPPP--
-PGQ--NQMPPHHGQD------------------NMPPPPPPPHAGGGQPNYDSQMPNPQ
AGYNTG-----GAPH---------------------------------------------
-----YQQGGAPGYQGGPPGY---QGGNQGYHGNP--------G--A-------AYQGG-
-----------------PPGYPGGNPA-------------------------PPYPRR--
-------------------------------------------------------
>ZmDAL5
--MASASRALL-----------------LSRALQAGASRRVPALLR-PVAAAA-SLLPAV
AGPAGAA---------------LGARVRCFATQPA---TSSLRDSS--PNWSN-RPP---

---KETILLDGCDFEHWLVIMEPPPGDASNPDI-TRDEIIDSYIKTLAQVV-GSEEEARQ
KIYSVSTRHYFGFGALVSEELSYKLKEI----PKVRWVLPDSYLDVKNKDYGGEPFINGQ
AVPYD-----------------PKYHEEWVRNNARANDR---------------NRRN-D
RPRNFD-------RSRNF------------------------------------------
---------------------------------------------DRRRENMQNYQ----
------------------------------------------------------------
------------------NRDG------------------------PPAQGFNGPPPP--
-PGQ--NQMPSHHSQG------------------NM--SPQPPHAGGGQPNYQPQMQNPQ
TGYNPG-----GAPH---------------------------------------------
-----YQQGAAPGYQGGPPGY---QG---GYQGNP--------G--P-------AYQGGN
TGYQGGNPPPPYQGSNPPPPYQGGSPPGPPPPYRGGSPPGPPPPYRGGNPNVPPYSGGGN
PGYPGGSPGYQGQGGNSNFQ-----------------------------------
>Sobic.001G485600
-----MASASR-----------ALL---LSRALQAGAASRRVPTLL-RPLAAA-ASLLPA
AAAAAAP----------------GAGVRCFATQPA---TSSLRDSS--PNWSN-RPP---
---KETILLDGCDFEHWLVVMEPPPGDASNPDI-TRDEIIDSYIKTLAQIV-GSEEEARQ
KIYSVSTRHYFAFGALVSEELSYKLKEM----PKVRWVLPDSYLDVKNKDYGGEPFINGE
AVPYD-----------------PKYHEEWVRNNARANER---------------SRRN-D
RPRNFD-------RSRNF------------------------------------------
---------------------------------------------ERRRENMQNFQ----
------------------------------------------------------------
------------------NRDG------------------------PPAQGFSGPPPP--
-PGQ--NQMPPQHVQG------------------NM--PPPPPHAGGGQPNYQPQMPNPQ
AGYNPG-----GAPH---------------------------------------------
-----YQQGGAPGYQGGPPGY---QGGNQGYQGNP--------G--P-------AYQGGN
PGYQGG-----------PPGYPGGNPP-------------PPPPYQGGNPNAPPYPGGGN
PGYPGGSPGYQGQGGNSNFQ-----------------------------------
>Al_892358
----------------------MAI---SRSILRRPAKSFSSLFTRSFSSSSP-LANSPA
VSRSASSLLNRSRSLVAGFSALVRAGVSSARCMSTQATSSSLNDPN--PNWSN-KPP---
---KETILLDGCDFEHWLVVMEKPEGDL------TRDEIIDYYIKTLAQVV-GSEEEARM
KIYSVSHKCYFAFGALVSEDLSYKIKEL----PKVRWVLPDSYLDVKSKNYGGEPFIDGK
AVPYD-----------------PKYHEEWIKNNDSSN------------------SRT-R
RPRTLS-------GTRKF------------------------------------------
---------------------------------------------ERRRENVRGNQ----
------------------------------------------------------------
---------------------------------------DTGDRGPPPNQGLGGAPPPPP
HIGNNPNMPPHMPPPTM---------------NQNYRGPPPPNMGQNYQGPPPPNMNQNY
QGPPPPNMGQNY------------------------------------------------
------------------QGPLPPNMNQQNYQEPPPPNM---------------------
-----------------NQSYQGPPPSNMGQNYRGPSPPPPNMSQNYQGPPPPNMNGGWS
GNYQQNGGYQQGQGGGMQQQPYPPNRVQ---------------------------
>Al_478926
MATHTISRSILCRP--------AKS---LSLLFTRSFASSAPLVKIPASSLLS-RSRPLV

AAFSSVF----------------RGGLVSVKGLSTQATSSSLNDPN--PNWSN-RPP---
---KETILLDGCDFEHWLVVVNPPEGDP------TRDDIIDSYIKTLAQIV-GSEDEARM
KIYSVSTRCYYAFGALVSEDLSHKLKEL----PNVRWVLPDSYLDVRNKDYGGEPFIDGK
AVPYD-----------------PKYHEEWIRNNARANER---------------NRRN-D
RPRNFD-------RTRNF-----------------------------------------
---------------------------------------------ERRRENMAGGP----
------------------------------------------------------------
--------------------------PPQRPPMGGPPPPPHMGGSAPPPPHMGGSAPPPP
HMGQNYGGPPPPN-------------------NMGGQRPPPNYGGPPQNNMGGQRPPPNY
GGAPP-------------------------------------------------------
-----------PNYGGAPPANNMGGGPPPNYGGVP-------------------------
---------------------PPNYGAAPPPNYGGAPPQNNNYQQQSGGMQQPQYQNNYP
PNRDGSGNPYQG-------------------------------------------
>AT3G15000
MATHTISRSILCRP--------AKS---LSFLFTRSFASSAPLAKS-PASSLL-SRSRPL
VAAFSSV---------------FRGGLVSVKGLSTQATSSSLNDPN--PNWSN-RPP---
---KETILLDGCDFEHWLVVVEPPQGEP------TRDEIIDSYIKTLAQIV-GSEDEARM
KIYSVSTRCYYAFGALVSEDLSHKLKEL----SNVRWVLPDSYLDVRNKDYGGEPFIDGK
AVPYD-----------------PKYHEEWIRNNARANER---------------NRRN-D
RPRNND-------RSRNF-----------------------------------------
---------------------------------------------ERRRENMAGGP----
------------------------------------------------------------
--------------------------PPQRPPMGGPPPPPHIGGSAPPPPHMGGSAPPPP
HMGQNYGPPPPN--------------------NMGGPRHPPPYGAPPQNNMGGPRPPQNY
GGTPP-------------------------------------------------------
-----------PNYGGAPPANNMGGAPPPNYGGGP-------------------------
---------------------PPQYGAVPPPQYGGAPPQNNNYQQQGSGMQQPQYQNNYP
PNRDGSGNPYQG-------------------------------------------
>Potri.011G112200.1
-----MATSLLTRSLLLQKITSKTI---ISSILSRPFTSLSATSSA-SSTLLR-RALRPL
SAAANIN---------------RSVSRISSRSFSVNPSSSSLNDPS--PNWSN-RPP---
---KETILLDGCDFEHWLVVMDKPEGDP------TRDEIIDSYIKTLAEVV-GSEEEARK
KIYSVSTRCYFAFGALVSEEVSYKIKEL----KNVRWVLPDSYLDVKNKDYGGEPFIDGK
AVPYD-----------------PKYHEEWIRNNARANER---------------NRRN-D
RPRNVD-------RSRNF-----------------------------------------
---------------------------------------------DRRRENMQQRD----
------------------------------------------------------------
-------------GAPPPPMANQAMQNPAPNVAGHPQNMGRQGGLPPSPQNSYRVGPGGP
PPNNYNMGGPP---------------------NIGGPGGPRPNNYAGGQQNNMNRGPQNM
PPQNYMPPQNNMPLQ---------------------------------------------
-----------------------NNMPPQNYVPP--------------------------
-----------------QNNYIPPQNNMPPQNNMGGWSSDMPGNMQHNFQSGPNDGGYQG
GPPNYQNSYPPSRD-----------------------------------------
>Potri.001G393400.1

-----MATSLFTRSLFLQKPTSQTL---ISSLLSRAFTSHSSTSTACSSSLLR-RFLRPL
SSATTIN---------------RSVSRISIRSFSSSQKSSSLNDPS--PNWSN-RPP---
---KETILLDGCDFEHWLVVMEKPEGDP------TRDEIIDSYIKTLAQVV-GSEEEARR
KIYSVSTRCYYAFGALVPEEVSYKIKEL----KNVRWVLPDSYLDVKNKDYGGEPFIDGK
AVPYD-----------------PKYHEEWIRNNARANER---------------NRRN-D
RPRNVD-------RSRNF------------------------------------------
---------------------------------------------DRRMENMQPRG----
------------------------------------------------------------
-------------GAPPPPMANQAMRNPAPNMAGQPQNMGRQGA-PPQPQNNYR-GPGGP
PPNNYNMGGPGGPPPNNY--------------NVGGPGGPPPNNYMGGQQNNMSRVPHNM
PMQNYIPPQNNMPPQN--------------------------------------------
------------------------NMPPQNYTPQ--------------------------
-----------------QHNYIPPQNNMPPHNNTGGWSNDMPGNMQHNIQNEPANGGYQG
GPSNYQNKYPPSQDAV---------------------------------------
>A1_902623
-MAKTLSRSTA-----------SRIANRLFSTSKAAASPSPLPSHLISRRSSP-TIFHAV
GYIPALT---------------RFTTIRTRMDRSGGS-YSPLKSG---SNFSD-RPP--T
EMAP---LFPGCDYEHWLIVMEKPGGEN----A-QKQQMIDCYVQTLAKIV-GSEEEAKK
KIYNVSCERYFGFGCEIDEETSNKLEGL----PGVLFVLPDSYVDPEFKDYGAELFENGE
VVPRP-----------------PERQRRMVELTTQ--------------------RGS-D
KPKYHD-------RTR-N------------------------------------------
---------------------------------------------VRRRENMR-------
------------------------------------------------------------
------------------------------------------------------------
------------------------------------------------------------
------------------------------------------------------------
------------------------------------------------------------
------------------------------------------------------------
-------------------------------------------------------
>A1_921093
-MAKTLSRSTA-----------SRIANRLFSTSKAAASPSPLPSHLISRRSSP-TIFHAV
GYIPALT---------------RFTTIRTRMDRSGGS-YSPLKSG---SNFSD-RPP--T
EMAP---LFPGCDYEHWLIVMEKPGGEN----A-QKQQMIDCYVQTLAKIV-GSEEEAKK
KIYNVSCERYFGFGCEIDEETSNKLEGL----PGVLFVLPDSYVDPEFKDYGAELFENGE
VVPRP-----------------PERQRRMVELTTQ--------------------RGS-D
KPKYHD-------RTR-N------------------------------------------
---------------------------------------------VRRRENMR-------
------------------------------------------------------------
------------------------------------------------------------
------------------------------------------------------------
------------------------------------------------------------
------------------------------------------------------------
------------------------------------------------------------
-------------------------------------------------------

```
>AT2G35240
-MAKTLSRSTA-----------SCVAKRFFSTSNAVASPSPLPSHLISRRFSP-TIFHAV
GYIPALT---------------RFTTIRTRMDRSGGS-YSPLKSG---SNFSD-RPP--T
EMAP---LFPGCDYEHWLIVMEKPGGEN----A-QKQQMIDCYVQTLAKIV-GSEEEARK
KIYNVSCERYFGFGCEIDEETSNKLEGL----PGVLFVLPDSYVDPEFKDYGAELFVNGE
VVPRP-----------------PERQRRMVELTNQ--------------------RGS-D
KPKYHD-------RIR-N------------------------------------------
---------------------------------------------VRRRENMR-------
------------------------------------------------------------
------------------------------------------------------------
------------------------------------------------------------
------------------------------------------------------------
------------------------------------------------------------
------------------------------------------------------------
-------------------------------------------------------
>Al_473448
-MAKTLARSTA-----------SRI---TKRFFSTSGATTPSPSYLLSRRSTP-AISHAV
GFVSSLN---------------RLTTIRTRMDRSGGS-YSPLKSG---SNFSD-RPP--T
EMAP---LFPGCDYEHWLIVMDKPGGEN----A-TKQQMIDCYVQTLAKIL-GSEEEAKK
KIYNVSCERYFGFGCEIDEETSNKFEGL----PGVLFVLPDSYVDQENKDYGAELFVNGE
IVQRP-----------------PERQRKIIELTTQ--------------------RSN-D
KPKYHD-------KTR-Y------------------------------------------
---------------------------------------------VRRRENMR-------
------------------------------------------------------------
------------------------------------------------------------
------------------------------------------------------------
------------------------------------------------------------
------------------------------------------------------------
------------------------------------------------------------
-------------------------------------------------------
>AT1G32580
-MAKTLARSTA-----------SRI---TKRLISTSGATTPSPSYILSRRSTP-VFSHAV
GFISSLN---------------RFTTIRTRMDRSGGS-YSPLKSG---SNFSD-RAP--T
EMAP---LFPGCDYEHWLIVMDKPGGEN----A-TKQQMIDCYVQTLAKII-GSEEEAKK
KIYNVSCERYFGFGCEIDEETSNKLEGL----PGVLFILPDSYVDQENKDYGAELFVNGE
IVQRP-----------------PERQRKIIELTTQ--------------------RTN-D
KPKYHD-------KTR-Y------------------------------------------
---------------------------------------------VRRRENMR-------
------------------------------------------------------------
------------------------------------------------------------
------------------------------------------------------------
------------------------------------------------------------
------------------------------------------------------------
------------------------------------------------------------
```

-------------------------------------------------------
>LOC_Os04g51280
-----MATAAA-----------------AARAVAAAGRPAQGVPLS-RRLTTA-SSSSAR
PLRPRGG--------------RAAGSVRCMARRPE-SSYSPLRS----GQGGD-RAP--T
EMAP---LFPGCDYEHWLIVMDKPGGEG----A-TKQQMIDCYIQTLAKVV-GSEEEAKK
KIYNVSCERYFGFGCEIDEETSNKLEGL----PGVLFVLPDSYVDAENKDYGAELFVNGE
IVQRS-----------------PERQRR-VEPVPQ--------------------RAQ-D
RPRYSD-------RTR-Y------------------------------------------
---------------------------------------------VKRRENQAYQR----
------------------------------------------------------------
------------------------------------------------------------
------------------------------------------------------------
------------------------------------------------------------
------------------------------------------------------------
------------------------------------------------------------
-------------------------------------------------------
>Bradi5g20660.1
----------------------------MATAARAIAAARLAQPLLVSRRLPS-SSARPT
RPRGGGG-----------------SAVRCMARRPDAS-YSPLRS----GQGGD-RAP--T
EMAP---LFPGCDYEHWLIVMDKPGGEG----A-TKQQMIDCYIQTLAKVV-GSEEEAKK
KIYNVSCERYFGFGCEIDEETSNKLEGL----PGVLFVLPDSYVDAENKDYGAELFVNGE
IVQRS-----------------PERQRR-VEPVPQ--------------------RAQ-D
RPRYSD-------RTR-Y------------------------------------------
---------------------------------------------VKRRENQAYQR----
------------------------------------------------------------
------------------------------------------------------------
------------------------------------------------------------
------------------------------------------------------------
------------------------------------------------------------
------------------------------------------------------------
-------------------------------------------------------
>ZmDAL1
----------------------------MATAARALVAARPARPLL-PSRRLP-SSSSIR
PARQRAG----------------VGCVRCMARRPD-STYSPLRS----GQGGD-RAP--T
EMAP---LFPGCDYEHWLIVMDKPGGEG----A-TKQQMIDCYIQTLAQVV-GSEEEAKK
RIYNVSCERYFGFGCEIDEETSNKLEGL----PGVLFVLPDSYVDAENKDYGAELFVNGE
IVQRS-----------------PERQRR-VEPVPQ--------------------RAQ-D
RPRYSD-------RTR-Y------------------------------------------
---------------------------------------------VKRRENQSYQR----
------------------------------------------------------------
------------------------------------------------------------
------------------------------------------------------------
------------------------------------------------------------
------------------------------------------------------------

------------------------------------------------------------
-------------------------------------------------------
>Sobic.006G204100
----------------------------MATAARALVAARPARPLLPSRRLPSSSSIRPP
RQRGGGV-----------------GSVRCMARRPDSS-YSPLRS----GQGGD-RAP--T
EMAP---LFPGCDYEHWLIVMDKPGGEG----A-TKQQMIDCYIQTLAQVV-GSEEEAKK
RIYNVSCERYFGFGCEIDEETSNKLEGL----PGVLFVLPDSYVDAENKDYGAELFVNGE
IVQRS-----------------PERQRR-VEPVPQ--------------------RAQ-D
RPRYSD-------RTR-Y------------------------------------------
---------------------------------------------VKRRENQSYQR----
------------------------------------------------------------
------------------------------------------------------------
------------------------------------------------------------
------------------------------------------------------------
------------------------------------------------------------
------------------------------------------------------------
-------------------------------------------------------
>Bradi3g00440.1
-----MARKLLSSPS-------------RSLLLCRHISSSSSSSSFMRGGGRS-SPLPLM
DLLRPAA-------SSSFFLHRLGGATRGMARRPGGDGYSPARSG---GGGGD-RAP--S
EMAP---LFPGCDYEHWLIVMDKPGGEG----A-TKQQMIDCYIQTLAKIL-GSEEEAKK
KIYNVSCERYFGFGCEIDEETSNKLEGI----PGVLFVLPDSYVDPENKDYGAELFVNGE
IVQRS-----------------PERQRR-VEPVPQ--------------------RAS-D
RPRYND-------RTR-Y------------------------------------------
---------------------------------------------ARRRENQR-------
------------------------------------------------------------
------------------------------------------------------------
------------------------------------------------------------
------------------------------------------------------------
------------------------------------------------------------
------------------------------------------------------------
-------------------------------------------------------
>Bradi1g50640.2
--MAAAAAAGA-----------RRL---LSRRASSSASSISSLLRR--GPCVA-AAVHEP
LLRPAVV-------------APRLGFLRGMARRPGGDGYSPTRSGG--GGGGE-RAP--T
EMAP---LFPGCDYEHWLIVMDKPGGEG----A-TKQQMIDCYIQTLAKIL-GSEEEAKK
KIYNVSCERYFGFGCEIDEETSNKLEGI----PGVLFVLPDSYVDPENKDYGAELFVNGE
IVQRS-----------------PERQRR-VEPVPQ--------------------RAS-N
RPRYND-------RTR-Y------------------------------------------
---------------------------------------------ARRMENQQR------
------------------------------------------------------------
------------------------------------------------------------
------------------------------------------------------------
------------------------------------------------------------

------------------------------------------------------------
------------------------------------------------------------
-------------------------------------------------------

>Bradi1g42860.2
------MAAGA-----------AAG---ARRLLSRRASSSVSALLR-RGPSVA-AAVHEP
LLRPAVV-------------APRLGFLRGMARRPGGDGYSPTRSGG--GGGGE-RAP--T
EMAP---LFPGCDYEHWLIVMDKPGGEG----A-TKQQMIDCYIQTLAKIL-GSEEEAKK
KIYNVSCEQYFGFGCEIDEETSNKLEGI----PGVLFVLPDSYVDPENKDYGAELFVNGE
IVQRS-----------------PERQRR-VEPVPQ--------------------RAS-D
RPRYND-------RTR-Y------------------------------------------
---------------------------------------------AWRRENQQR------
------------------------------------------------------------
------------------------------------------------------------
------------------------------------------------------------
------------------------------------------------------------
------------------------------------------------------------
------------------------------------------------------------
-------------------------------------------------------

>LOC_Os06g02600
---MAAAAAAAA-----------------ARRLLSRRATSFSASALLRRGGPGA-PESLLR
PTVAAVS---------------RVGFLRGFARRPGGDGYSPMRSGGGGGGGGD-RAP--T
EMAP---LFPGCDYEHWLIVMDKPGGEG----A-TKQQMIDCYIQTLAKVL-GSEEEAKK
KIYNVSCERYFGFGCEIDEETSNKLEGL----PGVLFVLPDSYVDPEYKDYGAELFVNGE
IVQRS-----------------PERQRR-VEPVPQ--------------------RAS-D
RPRYND-------RTR-Y------------------------------------------
---------------------------------------------ARRRENQR-------
------------------------------------------------------------
------------------------------------------------------------
------------------------------------------------------------
------------------------------------------------------------
------------------------------------------------------------
------------------------------------------------------------
-------------------------------------------------------

>Sobic.010G013900
-----MAAAAAA-----------------ARRLLSRRASSSSLSALLRRGAAAE-QPLLLR
PAVVAAA--------------SRLGFPRGMARRPGGDGFGPTRP----GAGGD-RAP--S
DMAP---LFPGCDYEHWLIVMDKPGGEG----A-NKQQMIDCYIQTLAKVL-GSEEEAKR
KIYNVSCERYFGFGCEIDEETSNKLEGL----PGVLFVLPDSYVDPEYKDYGAELFVNGE
IVQRP-----------------PERQRR-VEPVPQ--------------------RSA-D
RPRYND-------RTR-Y------------------------------------------
---------------------------------------------ARRRENQR-------
------------------------------------------------------------
------------------------------------------------------------
------------------------------------------------------------

------------------------------------------------------------
------------------------------------------------------------
------------------------------------------------------------
-------------------------------------------------------

>ZmDAL3
-----MAAAAA-----------------AARRLLSRRASSNSLSAFLRRGAAP-EQSLLR
PAVVAAA--------------SRLGFPRGMARRPGGDGYGAGRD----------RAP--T
EMAP---LFPGCDYEHWLIVMDKPGGEG----A-TKQQMIDCYIQTLAKVL-GSEEEAKK
KIYNVSCERYFGFGCEIDEETSNKLEGL----PGVLFVLPDSYVDPEYKDYGAELLVNGE
IVQRP-----------------PERQRR-VEPVPQ--------------------RAA-D
RPRYND-------RTR-Y------------------------------------------
---------------------------------------------ARRRENQR-------
------------------------------------------------------------
------------------------------------------------------------
------------------------------------------------------------
------------------------------------------------------------
------------------------------------------------------------
------------------------------------------------------------
-------------------------------------------------------

>ZmDAL4
-----------------------MAAAAARRLLSRRASSSSLSALLRRGAVPE-QSLVLR
PAVVAAA--------------SRLGFQRGMARRPGGDGYVSTRS----GAGGD-RAPMAT
EMAP---LFPGCDYEHWLIVMDKPGGEG----A-SKQQMIDCYIQTLAKVL-GSEEEAKK
KIYNVSCERYFGFGCEIDEETSNKLEGL----PGVLFVLPDSYVDAEYKDYGAELFVNGE
IVQRT-----------------PERQRR-VEPVPQ--------------------RAA-D
RPRYND-------RTR-Y------------------------------------------
---------------------------------------------ARRRENQR-------
------------------------------------------------------------
------------------------------------------------------------
------------------------------------------------------------
------------------------------------------------------------
------------------------------------------------------------
------------------------------------------------------------
-------------------------------------------------------

>Potri.008G169900.1
-MTPTLARSLTTRHLN------LCVLLPKRLLSTISITHLPSPPTLLCGQSLP-SLSHNL
QSINKTT-----------NPAARFTSIRCRVNRAGNSGYSPLNSG---SNFSD-RPP--N
EMAP---LFPGCDYEHWLIVMDKPGGEG----A-TKQQMIDCYIETLAKVV-GSEEEAKT
KIYNVSCERYFGFGCEIDEETSNKLEGLFRCLPGVLFVLPDSYVDPEYKDYGAELFVNGE
IVQRP-----------------PERQRR-VEPQPQ--------------------RAN-D
RPRYND-------RTR-Y------------------------------------------
---------------------------------------------VRRRENMR-------
------------------------------------------------------------
------------------------------------------------------------

------------------------------------------------------------
------------------------------------------------------------
------------------------------------------------------------
------------------------------------------------------------
-------------------------------------------------------

>Potri.010G068300.1
-MTPTLVRSLATRHLS------LCVLLPKRLLSTFSITHPPSLPTVLPCRQSLPSLSHAL
QSINKTT------------NPTRFTSIRCRVNRAGNSGYSPLNSG---SNFSD-RPP--N
EMAP---LFPGCDYEHWLIVMDKPGGEG----A-TKQQMIDCYIQTLSKVV-GSEEEAKN
KIYNVSCERYFGFGCEIDEETSNKLEGL----PGVLFVLPDSYVDPEYKDYGAELFVNGE
IVQRP-----------------PERQKR-VEPQPQ--------------------RAN-D
RPRYND-------RTR-Y------------------------------------------
---------------------------------------------VRRRENMR-------
------------------------------------------------------------
------------------------------------------------------------
------------------------------------------------------------
------------------------------------------------------------
------------------------------------------------------------
------------------------------------------------------------
-------------------------------------------------------

>Al_482286
----------------------MALPLSGTRHLTRALLSNVTVI---APPRVP-SSVHCG
GSRFGYS--------------TRFFSIRCGANRSGSA-YSPLNSG---SNFSD-RPP--T
EMAP---LFPGCDYEHWLIVMDKPGGEG----A-TKQQMIDCYIQTLAKVV-GSEEEAKK
RIYNVSCERYLGFGCEIDEETSTKLEGL----PGVLFVLPDSYVDPENKDYGAELFVNGE
IVQRS-----------------PERQRR-VEPQPQ--------------------RAQ-D
RPRYND-------RTR-Y------------------------------------------
---------------------------------------------SRRRENTR-------
------------------------------------------------------------
------------------------------------------------------------
------------------------------------------------------------
------------------------------------------------------------
------------------------------------------------------------
------------------------------------------------------------
-------------------------------------------------------

>AT2G33430
----------------------MALPLSGTRHLTRALLSNVTLM---APPRIP-SSVHYG
GSRLGCS--------------TRFFSIRCGANRSGST-YSPLNSG---SNFSD-RPP--T
EMAP---LFPGCDYEHWLIVMDKPGGEG----A-TKQQMIDCYIQTLAKVV-GSEEEAKK
RIYNVSCERYLGFGCEIDEETSTKLEGL----PGVLFVLPDSYVDPENKDYGAELFVNGE
IVQRS-----------------PERQRR-VEPQPQ--------------------RAQ-D
RPRYND-------RTR-Y------------------------------------------
---------------------------------------------SRRRENTR-------
------------------------------------------------------------

------------------------------------------------------------
------------------------------------------------------------
------------------------------------------------------------
------------------------------------------------------------
------------------------------------------------------------
-------------------------------------------------------
>Bradi4g22160.1
----------------------MALALRLRRALAAASTSTSAPLLLRPAVSVA-ASSSRS
LLLPAPF--------------VPPLPRPFLPGAAAAAGFRSTAAAAARGDYG--RGADEN
NIGPDEILFEGCDYNHWLITMEFPDPKP------SREEMIETFLQTLAQVV-GSYEEAKK
RMYALSTTTYVGFQAEITEEMSEKFRGM----PGVVFILPDSYLYPETKEYGGDKYDNGV
ITPRP-----------------PPV----QYSKPQ--------------------RTD-R
NRSYGGNYQNSPPRGN-F------------------------------------------
----------------------QNSPPPRGNYQNSPPPPGNYQNSPPPQGNFQTYS----
------------------------------------------------------------
-----------------AHQDRRGYAPQQNYAQTGQDSRGYGRNDSANQSGYNGPPGGYQ
GQANQAGQGYQ---------------------NPQEHRNFSQGQAGDFRSTGTSAPGNYG
QPSAPGTYGQPSGSATFG------------------------------------------
------------------------------------------------------------
-----------------QPSGPGAYGQPAAPTNPGANQGGPGYGGDNRQGAGPAYGGDNL
QRGSSQYPSPNEGQGNWQGRQ----------------------------------
>LOC_Os11g11020
----------------------MALALRLRRALAAASTSASPFLRPAASASRS-APLAAA
PLPPPHQ--------------VSRPWLPAAGFRSSAAASAAARG----DDYG--RRDVDE
KISPDEILFEGCDYNHWLITMEFPDPKP------TREEMIETYLQTLAKVV-GSYEEAKK
RMYAFSTTTYVGFQAVMTEEMSEKFRGL----PGVVFILPDSYLYPETKEYGGDKYENGV
ITPRP-----------------PPV----HYSKPS--------------------RTD-R
NRNY---------RGN-Y------------------------------------------
-------------------------------------------QNGPPQGNYQNSP----
------------------------------------------------------------
----------------------------PPYGSQQDGRGYAPRQNYADRPGYSGTSGGYQ
SQTTQYQGHANPAGQGQGYY------------NSQERRNFNQGQGGDFRPGGPSAPGTYG
QPPAPG------------------------------------------------------
------------------------------------------------------------
-------------------NYAQPHPPTYPGSNQGAPGVNPGYGGNNRQGPGPAYGGDNW
QGGSNQYPSQSEGQQESWRGRQ---------------------------------
>ZmDAL2
----------------------MAL---ALRLRRVLAAASTAAPLLRPSTSVA-RPCPLV
PLASPVA---------------PLPLPPWRFLPGGGAGFRSTAAAA--ARGGTDYGTDDS
KISPDEILFEGCDYNHWLITMDFPDPKP------SREEMIETYLQTLAKVV-GSYEEAKK
RMYAFSTTTYVGFQAVMTEEMSEKFRGL----PGVVFILPDSYLYPETKEYGGDKYDNGV
ITPRP-----------------PPV----HYSRPS--------------------RTD-R
NRNY---------RGN-Y------------------------------------------
---------------------QDGPPQQGNYQNNRPPPEGGYQNNPPQQGNYQTYR----

------------------------------------------------------------
-----------------SQQDGRGYAPQQNYAQGGQDGRGFGRNDYTDRSGYNGPPDFRS
QTQYQGHVNPAGQGQGYN--------------NPQERSNFSQGQGGGFRPGGPSAPGSYG
QPSAPGSYGQPN------------------------------------------------
------------------------------------------------------------
-----------------TLGNYGQVPPSVNPGGNRVPGVNPSYGGDGRQGAGPAYGGDNW
QRGSGQYPSPGEGQGNWQGRQ----------------------------------
>Sobic.005G100900
--------------------------MALALRFRRVLAAASTSAPLLRPSASV-ARPSPL
AAPASSP--------------VAPLPRAPWRLLPGGAAAGFRSTAAAAARGGADYGASDS
KISPDEILFEGCDYNHWLITMEFPDPKP------SREEMIETFLQTLAKVV-GSYEEAKK
RMYAFSTTTYVGFQAVMTEEMSEKFKGL----PGVVFILPDSYLYPETKEYGGDKYDNGV
ITPRP-----------------PPIH----YSKPS--------------------RTD-R
NRNY---------RGN-Y------------------------------------------
---------------------QDGPQQQGNYQ--NRPQQGGYQNNPPQQGNFQTNR----
------------------------------------------------------------
-----------------SQQDGRGYAPQRNYAQGGQDGRGFGRNDYADRSGYNGPPGGFQ
GQAQ-YQGHVNPAGQDQGYN------------NPQERRNFPQGQGGGYRPGGPSAPGSFG
QQSAPGSFGQQSAPG---------------------------------------------
--SYGRPSAPGSYGQPSTPGSYGQPSTPGSYGQSS-------------------------
-----------------TPGNYGQAPPSANPG-GRVPGANPSYGGDGRQGTGPAYGGDNW
QRDSGQYPSPGEGQGNWQGRQ----------------------------------
>Aquca_095_00030
------------------------------------------------------------
------------------------------------------------------------
--------------------MDFPKDPK----P-TPEQMVETYVHTLAQVV-GSVEEAKK
KMYACSTTTYQGFQAEITEEESEKFRGL----PGVVFILPDSYVDPVNKEYGGDKYINGT
IIPRP-----------------PPQIQRQGRYNDR--------------------NRG-D
RTG--------------Y------------------------------------------
---------------------------------------------DRPRSPMPEQQ----
------------------------------------------------------------
---------------GRPSDDRRGFGQGGERNYRGPQNFGPPQN-FGPS---HGDGRNYV
PQ------QNFGPNPT----------------SQGDGGNFGAQQNYQPQQTYNPIPTGQG
EGRDTVPMGNRDYV----------------------------------------------
------------------------SGGRDNYQGAQRNPMPSYQTNIP-------------
-----------------QEGRREFDPPENRDFRGDGRNFGPSPGGDYRQGAAPS------
-------------------------------------------------------
>Aquca_039_00101
----------------------MAF---SSLVLRRTLTLSLRSLSS-ISSITT-SLQNPR
NYQKPHQ--------------ISNTGISQSIILRNSSSYSSSNNRYRYNNDND-----DE
KISPDTILFEGCDYKHWLITMDFPKDPA----P-SPEQMVETYVNTLAQVV-GSVEEAKK
KMYACSTTTYHGFQAEISEEESEKFKGL----PGVVFILPDSYIDTVNKEYGGDKYINGT
IIPRP-----------------PPVQTQRGRYGDR--------------------NRG-E
RTG--------------Y------------------------------------------

---------------------------------------------DRPRNPMPEQQ----
------------------------------------------------------------
---------------SRPSDDRQGFGQEGERNYRGPQNYGPSQN-FGPSPPPRGDGRNYV
PQQNYPPPQNFGPNPT----------------SQGDRRNFGAQQTYGPSPTGQGERRDSV
PMGPYVPGGRDNYQR---------------------------------------------
----------------------EQQNPMPSYQTNI-------------------------
-----------------PQEGRRDFAPENGDFRGDGRNFGPSAGGDYRQGATPSYGQNYP
AHGEGQRSPQMDQSDGMRMRGEPRNLF----------------------------
>Potri.011G032900.1
----------------------MATFTLSSSLTPKTLTPSLSNLKPTFLTSLK-PQSWTC
SQLISAP---------------KIRYQPLITRAAVGSDYSARRSN---SSNDD-------
---RETILLPGCDYNHWLIVMEFPKDPA----P-TREQMIDTYLNTLATVL-GSMEEAKK
NMYAFSTTTYTGFQCTVDEATSEKFKGL----PGVLWVLPDSYIDVKNKDYGGDKYVNGE
IIPCT-----------------YPT----YQPKQR--------------------TTS-K
YEN-----------RR-Y------------------------------------------
---------------------------------------------ERRRDGPPPDR----
------------------------------------------------------------
------------------------------------------------------------
-----------------------------------------------RRTRQGTTKSEPA
SP----------------------------------------------------------
------------------------------------------------------------
------------------------------------------------------------
-------------------------------------------------------
>LOC_Os08g04450
---------------------------MASFPTTAAAAAAQAFAFALAPKPSS-SAAAPS
ALFPRTA---------------AAAAFPTLAVRGSGKARQPVVAAAAGAGTGS-------
-EQRETILLPGCDYNHWLIVMEFPKDPA----P-TREQMIDTYLNTLATVL-GSMEEAKK
NMYAFSTTTYTGFQCTVDEETSEKFKGL----PGVLWVLPDSYIDVKNKDYGGDKYINGE
IIPCT-----------------YPT----YQPKER--------------------RTS-K
YES-----------RR-Y------------------------------------------
---------------------------------------------ERRRDGPPASR----
------------------------------------------------------------
------------------------------------------------------------
------------------------------------------------RPRPQTAQPESA
SSS---------------------------------------------------------
------------------------------------------------------------
------------------------------------------------------------
-------------------------------------------------------
>Bradi3g14650.1
----------------------------MASLPTAAATARVAATRSFAFPARP-SFSAST
AALPRAS-----------SAGTVFPAIALAAAAAAAGRAMRLTAARAAPGSEQ-------
---RETILLPGCDYNHWLIVMEFPKDPA----P-TREQMIDTYLNTLATVL-GSMEEAKK
NMYAFSTTTYTGFQCTVDEETSEKFKGL----PGVLWVLPDSYIDVKNKDYGGDKYINGE
IIPCT-----------------YPT----YQPKER--------------------RTS-K

YES-----------RR-Y------------------------------------------
---------------------------------------------ERRRDGPPASR----
------------------------------------------------------------
------------------------------------------------------------
------------------------------------------------RPKQQATQPESA
SS----------------------------------------------------------
------------------------------------------------------------
------------------------------------------------------------
-------------------------------------------------------

>ZmDAL6
----------------------------MAASLPTTAARLAAPAFAYRSPNAPSATALPR
AAAFPAI---------------AVAAAPLRPRRAPRPVAAR-------AGGEE-------
---RETILLPGCDYNHWLIVMEFPKDPA----P-TREQMIDTYLNTLATVL-GSMEEAKK
NMYAFSTTTYTGFQCTVDEETSEKFKGL----PGVLWVLPDSYIDVKNKDYGGDKYVNGE
IIPCT-----------------YPT----YQPKER--------------------RTS-K
YES-----------RR-Y------------------------------------------
---------------------------------------------ERRRDGPPAAS-----
------------------------------------------------------------
------------------------------------------------------------
-----------------------------------------------RKPRQQAPAQTQT
ESASS-------------------------------------------------------
------------------------------------------------------------
------------------------------------------------------------
-------------------------------------------------------

>Sobic.007G034500
---------------------------MAASLPTTAAAARLAAQAFAFPSLKP-PSASAF
SALPRAA---------------AFPSFAVAAAAPPRP--RPARPTAARAAGDE-------
---RETILLPGCDYNHWLIVMEFPKDPA----P-TREQMIDTYLNTLATVL-GSMEEAKK
NMYAFSTTTYTGFQCTVDEETSEKFKGL----PGVLWVLPDSYIDVKNKDYGGDKYINGE
IIPCT-----------------YPT----YQPKER--------------------RTS-K
YES------------RRY------------------------------------------
---------------------------------------------ERRRDGPPAAS-----
------------------------------------------------------------
------------------------------------------------------------
-----------------------------------------------RKPRQQAPQTESA
SS----------------------------------------------------------
------------------------------------------------------------
------------------------------------------------------------
-------------------------------------------------------

>Aquca_028_00257
---------------------MATF---NAAIPSKTLIKSFSTSISQTPIISS-FKFNSF
SCFPSSK---------------NISTLSSFSFSRTSSIKAAVD-----SDYSSKRSS--S
NEPRETILLPGCDYNHWLIVMEFPKDPA----P-TREQMIDTYLNTLATVV-GSMEEAKK
NMYAFSTTTYTGFQCTVSEETSEKFKGL----PGVLWVLPDSYIDVKNKDYGGDKYINGE

IIPCK-----------------YPT----YQPKQR--------------------SGS-K
YES-----------KR-Y------------------------------------------
---------------------------------------------ERRKSGPPAEK----
------------------------------------------------------------
------------------------------------------------------------
-----------------------------------------------RRPKQEVTQSDSA
SG----------------------------------------------------------
------------------------------------------------------------
------------------------------------------------------------
-------------------------------------------------------

>Al_471280
-----------------------------MASLTTSSSSSLLPKTLLPVSHLN-RFSTLS
DIRVGDT---------------WTPLLRSISTAGSRRRVAIVKAATVDSDYSSKRSS--S
NEQRETIMLPGCDYNHWLIVMEFPKDPA----P-TREQMIDTYLNTLATVL-GSMEEAKK
NMYAFSTTTYTGFQCTIDEETSEKFKGL----PGVLWVLPDSYIDVKNKDYGGDKYINGE
IIPCT-----------------YPT----YQPKQR--------------------NNT-K
YQS-----------KR-Y------------------------------------------
---------------------------------------------ERKRDGPP-------
------------------------------------------------------------
------------------------------------------------------------
----------------------------------------PPE---QRKPRQEPAASDSS
------------------------------------------------------------
------------------------------------------------------------
------------------------------------------------------------
-------------------------------------------------------

>AT1G11430
-----------------------------MASFTTTSSSSLLLKTLLPVSHLN-RFSTLS
GIRVGDS---------------WTPLLRNISTAGSRRRVAIVKAATVDSDYSSKRSN--S
NEQRETIMLPGCDYNHWLIVMEFPKDPA----P-SRDQMIDTYLNTLATVL-GSMEEAKK
NMYAFSTTTYTGFQCTIDEETSEKFKGL----PGVLWVLPDSYIDVKNKDYGGDKYINGE
IIPCT-----------------YPTY----QPKQR--------------------NNT-K
YQS-----------KR-Y------------------------------------------
---------------------------------------------ERKRDGPPPPE----
------------------------------------------------------------
------------------------------------------------------------
----------------------------------------------QRKPRQEPAASDSS
------------------------------------------------------------
------------------------------------------------------------
------------------------------------------------------------
-------------------------------------------------------
